# Hominoid-Specific *De Novo* Protein-Coding Genes Originating from Long Non-Coding RNAs

**DOI:** 10.1371/journal.pgen.1002942

**Published:** 2012-09-13

**Authors:** Chen Xie, Yong E. Zhang, Jia-Yu Chen, Chu-Jun Liu, Wei-Zhen Zhou, Ying Li, Mao Zhang, Rongli Zhang, Liping Wei, Chuan-Yun Li

**Affiliations:** 1 Center for Bioinformatics, State Key Laboratory of Protein and Plant Gene Research, College of Life Sciences, Peking University, Beijing, China; 2 Key Laboratory of Zoological Systematics and Evolution, Institute of Zoology, Chinese Academy of Sciences, Beijing, China; 3 Institute of Molecular Medicine, Peking University, Beijing, China; University of California Davis, United States of America

## Abstract

Tinkering with pre-existing genes has long been known as a major way to create new genes. Recently, however, motherless protein-coding genes have been found to have emerged *de novo* from ancestral non-coding DNAs. How these genes originated is not well addressed to date. Here we identified 24 hominoid-specific *de novo* protein-coding genes with precise origination timing in vertebrate phylogeny. Strand-specific RNA–Seq analyses were performed in five rhesus macaque tissues (liver, prefrontal cortex, skeletal muscle, adipose, and testis), which were then integrated with public transcriptome data from human, chimpanzee, and rhesus macaque. On the basis of comparing the RNA expression profiles in the three species, we found that most of the hominoid-specific *de novo* protein-coding genes encoded polyadenylated non-coding RNAs in rhesus macaque or chimpanzee with a similar transcript structure and correlated tissue expression profile. According to the rule of parsimony, the majority of these hominoid-specific *de novo* protein-coding genes appear to have acquired a regulated transcript structure and expression profile before acquiring coding potential. Interestingly, although the expression profile was largely correlated, the coding genes in human often showed higher transcriptional abundance than their non-coding counterparts in rhesus macaque. The major findings we report in this manuscript are robust and insensitive to the parameters used in the identification and analysis of *de novo* genes. Our results suggest that at least a portion of long non-coding RNAs, especially those with active and regulated transcription, may serve as a birth pool for protein-coding genes, which are then further optimized at the transcriptional level.

## Author Summary

Ever since the pre-genomic era, people believed that “mother gene”-based mechanisms such as gene duplication were the major means of creating new genes. Recently, we and others reported several “motherless” protein-coding genes in human, challenging the conventional idea in that some protein-coding genes might have emerged *de novo* from ancestral non-coding DNAs. However, how these interesting proteins originated is a question that remained unaddressed. The ancestral non-coding DNA must become transcribed and gain a translatable open reading frame before becoming a protein-coding gene, but either order of these two steps is possible. Here, we performed a comparative transcriptome study in human, chimpanzee, and rhesus macaque to address these fundamental questions. We found that most of the hominoid-specific *de novo* protein-coding genes encoded long non-coding RNAs in rhesus macaque or chimpanzee, with similar transcript structure and correlated tissue expression profile, but the protein-coding genes often had higher transcriptional abundance. According to the rule of parsimony, we conclude that at least a portion of long non-coding RNAs, especially those with active and regulated transcription, may serve as a birth pool for protein-coding genes that are then further optimized at the transcriptional level, a pattern insensitive to the parameters used in the identification and analysis of *de novo* genes.

## Introduction

For decades, people believed that “mother gene”-based mechanisms such as gene duplication or its modified forms such as exon shuffling, gene fusion, gene fission, and retroposition are the major means of creating new genes [1]–[4]. All of these mechanisms modify pre-existing genes or mother genes as the building blocks for new genes. However, recently identified motherless genes or *de novo* genes in primates [5]–[8] and other species [9]–[12] challenged this idea, in that some protein-coding genes might have emerged *de novo* from ancestral non-coding DNAs, providing another explanation for the complex genetic background underlying species- or lineage-specific traits.

In spite of the discovery of dozens of *de novo* protein-coding genes, an issue of great complexity and general interest that remains poorly addressed is how they originated from ancestral non-coding DNAs [13]–[15]. The ancestral non-coding DNA must become transcribed and gain a translatable open reading frame (ORF) before becoming a protein-coding gene, but either order of these two steps seems possible [7]. The “transcription-first” hypothesis was initially raised in [12], [16]. Later on, functional genomics data seemed to favor this hypothesis, given the high transcriptional activity of the genome [13], [14], [17], [18]. A case-study in yeast further supported this model, showing that both coding and non-coding orthologs of BSC4 are transcribed across multiple species [10].

However, even if we assume that the ancestral non-coding locus is firstly transcribed, there are still two possibilities that need to be distinguished. First, such an event may represent some sort of transcriptional leakage or noise, and only after the locus acquires a functional ORF may the transcriptional profile become regulated and optimized. Second, alternatively, the ancestral locus may encode a functional non-coding RNA with a specific transcriptional profile and transcript structure, and after the acquisition of an ORF, the new protein-coding gene largely adopts the pre-existing transcriptional profile. If the latter possibility is true, we would expect to see that the transcriptional structure and profile of a *de novo* protein is correlated with that of an out-group species that has the corresponding DNA but does not encode the ORF. On the other hand, if the initial transcription is promiscuous before translation starts, we would not expect to find a strong correlation in terms of expression pattern and gene structure.

In this work, we identified 24 hominoid-specific *de novo* genes and performed a comparative transcriptome study in human, chimpanzee and rhesus macaque, on the basis of next-generation sequencing technology [19]. We found generally similar transcriptional structures and profiles between *de novo* proteins and corresponding non-coding loci in the chimpanzee or rhesus macaque, suggesting that most *de novo* proteins were born out of non-coding RNAs. More interestingly, although the transcription was largely correlated, the protein-coding version of *de novo* genes tended to show higher abundance than non-coding orthologs, hinting that the transcription of *de novo* genes continued to be optimized. Taken together, our work presents a “semi-product” model of origination and evolution of *de novo* genes.

## Results

### Identification and sequence features of hominoid-specific *de novo* protein-coding genes

**Figure 1:**
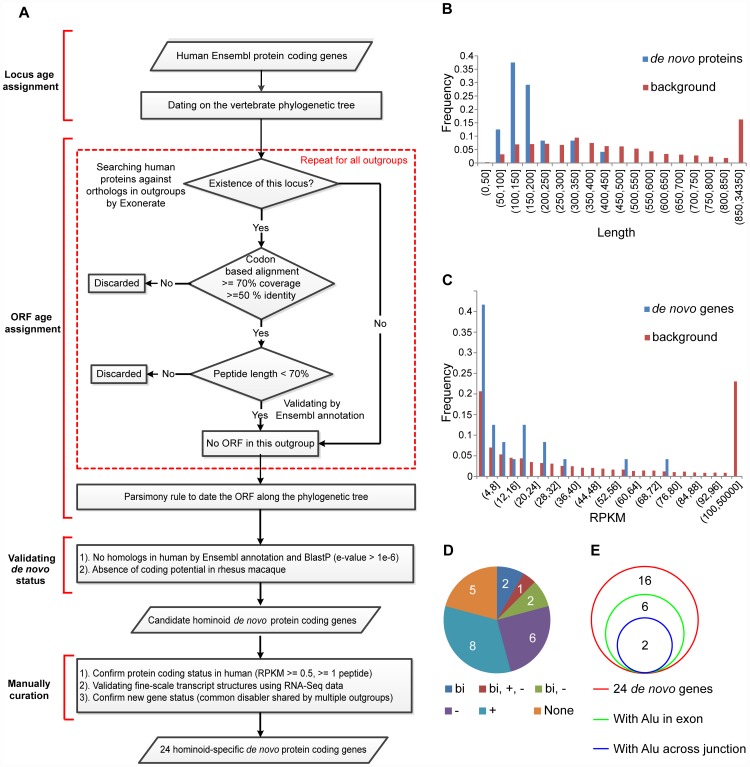
Genome-wide identification of hominoid-specific *de novo* protein-coding genes. (A) On the basis of the gene locus and ORF age assignments, hominoid-specific *de novo* protein-coding genes were identified. Regions within dotted red lines indicate the repeating steps for each out-group species. We further filtered this list using stringent inclusion criteria and generated a smaller convincing list of 24 *de novo* genes. (B) Distribution of protein length for the 24 *de novo* genes, compared with the human genome as background. (C) Distribution of summed RPKM scores of the 24 *de novo* genes in seven human tissues, compared with the human genome as background. (D) Pie chart showing the distribution of the 24 *de novo* protein-coding genes in terms of the reuse of preexisting transcriptional context. Gene numbers in each category are marked. None: no evidence for the reuse of transcriptional context; bi: located downstream of bi-directional promoter; +: overlapping with preexisting genes on the same strand; −: overlapping with preexisting genes on the opposite strand. (E) Venn diagrams showing the contribution of *Alu* sequences to exons and splicing junctions in *de novo* protein-coding genes.

We performed genome-wide identification of hominoid-specific *de novo* genes. On the basis of the locus age assignments, we assigned precise origination timing for the ORFs of these genes, inferred by summing up the information on the presence and absence of orthologs in vertebrate phylogeny with the rule of parsimony (Figure 1A, Materials and Methods). Sequence search in the human genome ensured that the new genes did not originate through other mechanisms such as gene duplication. Hominoid-specific *de novo* genes without coding potential in rhesus macaque were manually curated, using genome-wide expression filters to ensure the in-group transcriptional/translational expression, a newly-assembled transcriptome to validate the transcript structure in out-group species, and the identification of common ancestral disablers to ensure *de novo* origination rather than gene loss (Materials and Methods). In total, 24 hominoid-specific *de novo* protein-coding genes were identified: eleven encode proteins only in human (Class I) and thirteen encode proteins in both human and chimpanzee but not in rhesus macaque (Class II) (Figure 1A; Dataset S1; Tables S1, S2, S3).

We analyzed the characteristics of these *de novo* protein-coding genes. Consistent with previous reports [4], we found that the gene products were smaller, with a median length of 150.5 amino-acids, compared with 416 amino-acids in the human genome, suggesting the difficulty in *de novo* origination of long ORFs (Wilcoxon test, *p-value* = 4.06e-10, Figure 1B). The transcripts were generally expressed at lower levels compared with the human genome background (Wilcoxon test, *p-value* = 0.037, Figure 1C). Nineteen of the 24 *de novo* genes (79.2%) showed evidence to co-opt the transcriptional context, including seventeen (70.8%) that overlapped with another gene (nine anti-sense and nine at the same strand) and five (20.8%) located downstream of bi-directional promoters (Figure 1D, Table S2). Consistent with the case of *FLJ33706* or *C20orf203* [8], *Alu* elements contributed to eight exons in eight genes (33.3%), and to three splicing junction sites in two genes (Figure 1E, Tables S4, S5), further supporting the previous observation that repeat elements might be involved in the origination of some *de novo* genes [11].

### Transcriptomes across human, chimpanzee, and rhesus macaque

To test whether the ancestral non-coding locus of a *de novo* protein-coding gene is already transcribed in a regulated way, we performed comparative transcriptome profiling of 24 *de novo* genes between human and two relatives, the chimpanzee, which did not encode about half of the corresponding human ORFs and the rhesus macaque, which did not encode any.

**Figure 2:**
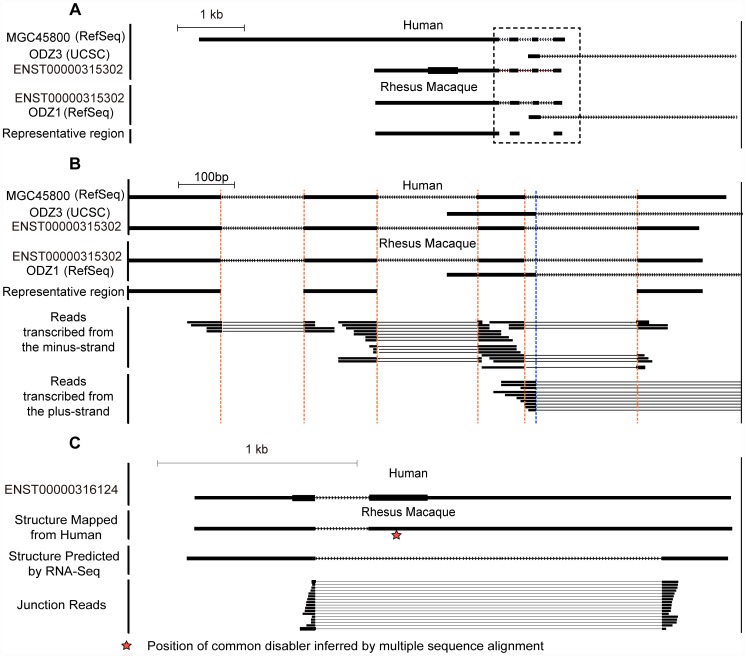
Strand-specific RNA–Seq in five rhesus tissues reveals clear transcript structure for *de novo* genes. (A) An example of *de novo* gene *ENST00000315302* partially overlapped with a pre-existing gene *ODZ3*, transcribed by the other strand of the DNA. The ortholog of *ENST00000315302* in rhesus macaque was aligned according to genome-wide multiple alignments in UCSC. The junction reads generated by strand-specific RNA-Seq assays are highlighted by black bold lines, with fragments of junction reads crossing splicing junctions connected by thinner lines. The mapped reads well supported the transcription of the target *de novo* gene on the reverse strand, as most reads appeared in the track for ‘reads transcribed from the minus-strand’. Regions for all four splicing junctions are highlighted in dotted boxes and expanded in (B), including three in *ENST00000315302* transcribed from the minus strand and one from the other strand. All of these splicing junctions were well supported by the RNA-Seq reads mapped on the corresponding strand of the DNA. Vertical dotted lines in brown or blue highlight the exon boundaries in transcripts on the minus or plus strands, respectively. (C) Demo case for a discarded *de novo* gene in the manual curation process, in which the RNA-Seq data in rhesus macaque were not consistent with the putative splicing pattern predicted on the basis of human gene models. The common disabler is marked with a red star, and this was actually spliced out in rhesus macaque as indicated by the junction reads. Scale bar shown as benchmark for gene size.

Because *de novo* genes tend to co-opt the transcriptional context by overlapping with neighboring and anti-sense genes, it is difficult to accurately define the gene structures, especially the gene borders, and determine the RNA expression using probe-based cDNA microarray technology or strand-non-specific RNA-Seq. We performed high-quality strand-specific RNA-Seq in five rhesus macaque tissues (liver, prefrontal cortex, skeletal muscle, adipose and testis) to identify polyadenylated RNAs (Table 1). A total of 477.7 million 90-bp paired-end reads were uniquely mapped to the rhesus macaque genome, covering over 18,000 polyadenylated RNAs with correct transcript strand information, clear transcript structure and accurate quantification of expression (Figure 2, Figure S1, Table 1). All data have been submitted to NCBI Gene Expression Omnibus (Accession Number: GSE34426).

**Table 1:** Statistics of RNA–Seq data.

Tissue	Description	Total Reads	Unique Genomic Reads		Unique Junction Reads	
Testis	90-bp, paired-end, strand-specific	100.7 M#	63.1 M	62.6%	12.8 M	12.7%
Prefrontal cortex	90-bp, paired-end, strand-specific	142.1 M	88.4 M	62.2%	12.8 M	9.0%
Adipose	90-bp, paired-end, strand-specific	120.4 M	74.8 M	62.1%	13.4 M	11.1%
Liver	90-bp, paired-end, strand-specific	160.4 M	94.4 M	58.8%	29.9 M	18.6%
Skeletal muscle	90-bp, paired-end, strand-specific	120.0 M	68.1 M	56.8%	20.0 M	16.7%
**Summary**		**643.6 M**	**388.8 M**	**60.4%**	**88.9 M**	**13.8%**

# M: million reads.

Based on this RNA-Seq dataset, we selected representative regions for the 24 *de novo* genes that did not overlap with other genes (Table S6, Materials and Methods). We then integrated all publicly available strand-non-specific RNA-Seq data including RNA-Seq data from seven human tissues [20], [21], two additional rhesus macaque tissues [20] and five chimpanzee tissues [20], [22] (Table S7, Materials and Methods). RNA expression levels were calculated in terms of RPKMs (Reads Per Kilobase of exon model per Million mapped reads) of representative regions in all three species. Splicing junctions were assembled. These efforts made it possible to study the transcript structure, transcription activity and tissue expression profile for these *de novo* loci in species with or without protein-coding potential.

### High correlation of non-coding genes with their protein-coding orthologs with respect to gene structure, transcription activity, and tissue expression profile

**Figure 3:**
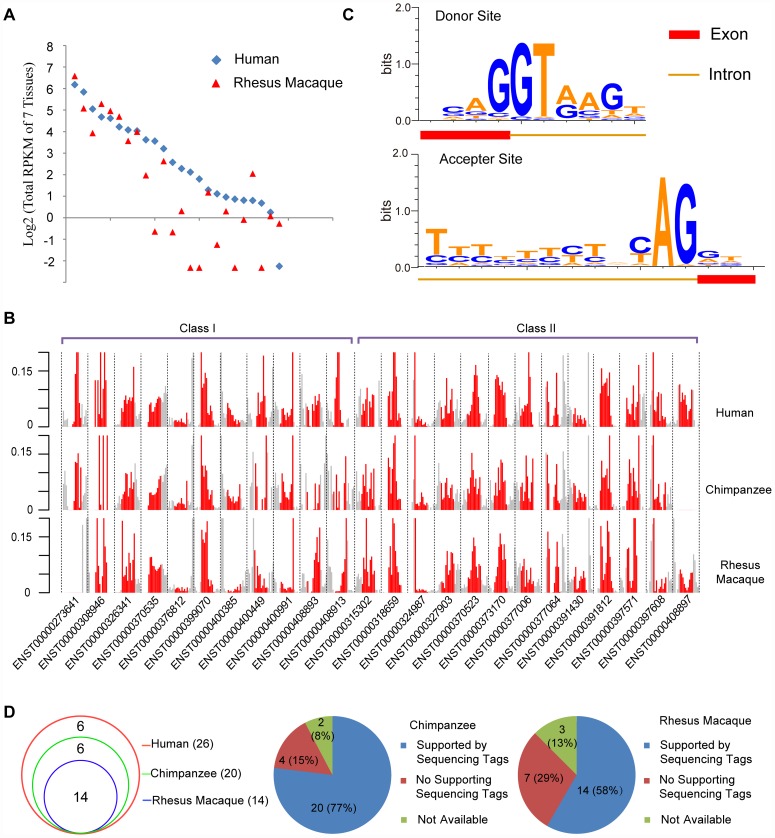
Orthologs of human *de novo* protein-coding genes encode structure-matched non-coding RNAs in rhesus macaque or chimpanzee. (A) Summed RPKM scores (log_2_ transformed) of *de novo* genes in seven tissues from human and rhesus macaque. The human genes were ordered by decreasing expression level as a reference, and the rhesus genes were aligned accordingly. (B) For each *de novo* gene in Classes I and II, the base-level densities of RNA-Seq reads across the transcript (red), as well as the upstream/downstream regions (grey, 50% of the length of the transcript), are shown. The raw density scores computed from RNA-Seq reads coverage were normalized with the total reads across the region. (C) Splicing junctions with the sequence motifs near both the donor site and acceptor site, summarized by all splicing junctions in human *de novo* genes. (D) Venn diagram showing the numbers of human splicing junctions detected also in chimpanzee or rhesus macaque. Pie charts further illustrate the detailed status of human splicing junctions in chimpanzee and rhesus macaque.

We found that 21 out of 24 (87.5%) hominoid-specific *de novo* genes appeared to be transcribed in at least one chimpanzee tissue, and twenty (83.3%) in at least one rhesus tissue as non-coding RNAs (Figure S2, Materials and Methods). However, eighteen showed lower expression levels in rhesus macaque than their protein-coding orthologs in human (Figure 3A, Table S8). Such a difference suggests that the *de novo* origination of protein-coding genes probably happened in a step-by-step fashion, in which the ancestral non-coding DNA may already have been transcribed before it acquired coding potential, while the expression level was further optimized at the transcriptional level after the coding potential was acquired.

We then compared the base-level density of RNA-Seq reads across the transcript with that in the upstream/downstream flanking regions. These *de novo* loci showed significantly higher expression than the corresponding flanking regions (Figure 3B). For both Class I and II genes, the distributions of expression densities were largely comparable among the three species, suggesting that the overall transcription structure of these *de novo* genes had been formed before these species diverged (Figure 3B). We further examined the exon/intron gene structure of these *de novo* transcripts. The 24 *de novo* genes formed 26 splicing junctions in human, each supported by at least one RNA-Seq read (Figure 3D, Table S9). Among these human splicing junctions, 24 in chimpanzee and 23 in rhesus macaque were located in regions with enough read coverage to infer the exon/intron structures (Figure 3D, Tables S10, S11). Among them twenty (20/24, 83.3%) in chimpanzee and fourteen in rhesus macaque (14/23, 60.9%) were supported by reads covering both donor and acceptor sites, forming exons separated by standard introns marked with GT-AG splicing junctions (Figure 3C and 3D, Figure S3). Thus the majority of human splicing junctions were detectable in chimpanzee and rhesus macaque, suggesting that the exon/intron structures of human *de novo* genes were largely shared by their non-coding orthologs.

**Figure 4:**
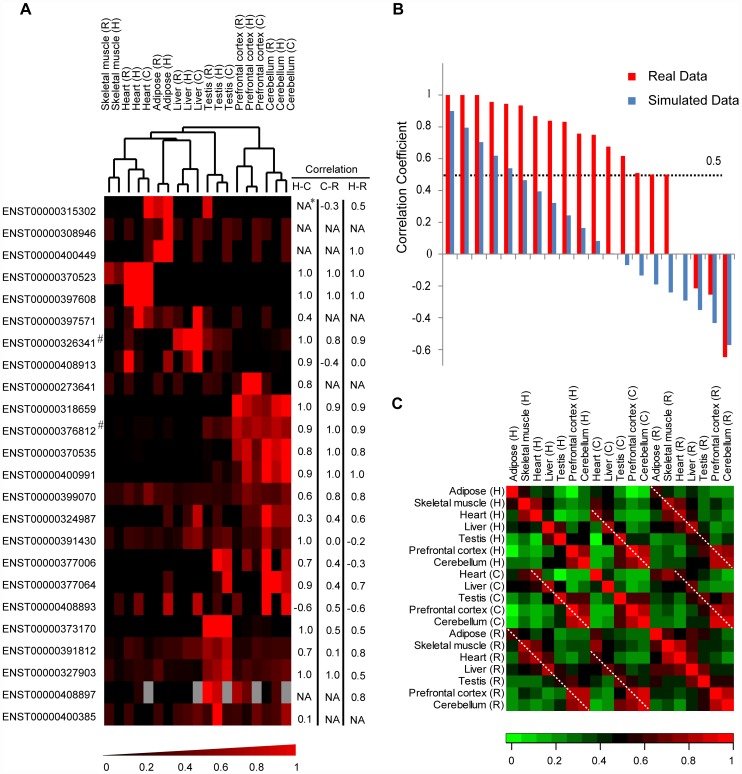
Non-coding orthologs of human *de novo* protein-coding genes in rhesus macaque and chimpanzee show tissue expression profiles similar to human. (A) Hierarchical clustering chart of tissue expression proportions. For each gene in one species, tissue expression proportions were calculated by normalizing RPKM scores with the total expression level of the gene in that species. The scores were then clustered according to similarity using complete linkage hierarchical clustering. For each gene, cross-tissue correlation coefficients between human and chimpanzee (H–C), chimpanzee and rhesus macaque (C–R) and human and rhesus macaque (H–R) are shown. (B) Correlation coefficient scores for tissue expression profiles between human and rhesus macaque. Correlation coefficients for *de novo* genes (brown histograms) are illustrated with background simulated by 10,000 *Monte Carlo* simulations neglecting ortholog relationship for the tissue expression profile (blue histograms, mean scores are shown). (C) For each pair of tissues, Spearman correlation coefficients were computed separately and the extent of tissue-specific differences in *de novo* gene expressions are shown. Dotted lines highlight comparisons between pairs of corresponding tissues in different species. Grey boxes: missing data. ^*^Correlation coefficient not available due to low tissue expressions in one or both species. ^#^Gene reported in previous study as human-specific *de novo* protein-coding gene.

Finally, we analyzed the expression profiles of these newly-originated *de novo* protein-coding genes. The *de novo* genes showed brain-enriched transcriptional expression (Figure 4A, Figure S4), consistent with the recent discovery of accelerated recruitment of new genes into the human brain [23] as well as other independent studies reporting the expression profiles of newly originated *de novo* genes in human [5], [7], [8]. On the basis of cross-species tissue expression profiles, we further investigated the transcriptional correlations of *de novo* genes across different tissues in human, chimpanzee and rhesus macaque. For the twenty *de novo* genes with convincing transcription in both human and rhesus macaque, sixteen in rhesus macaque (80.0%) showed tissue expression profiles similar to human (Figure 4A). Based on 10,000 *Monte Carlo* simulations neglecting ortholog relationships for the tissue expression profile, such an observation represents a statistically significant excess (*p-value*<10^−4^, Figure 4B, Materials and Methods). Transcriptome data from five chimpanzee tissues had similar patterns of correlation with human and rhesus macaque (Figure 4A). Considering these *de novo* genes as a whole, the hierarchical clustering further supported the between-species similarity of tissue expression profiles, in that the same tissues from different species were clustered together (Figure 4A). Spearman correlation coefficients were computed separately for each pair of tissues and the extent of tissue-specific differences in *de novo* gene expression were shown in Figure 4C. Besides the strongest correlations across the diagonal line representing self-comparisons, strong correlations were also detected for pairs of the same tissues in different species (shorter lines parallel to the diagonal line) (Figure 4C). No significant difference was detected for the expression profile correlation between the two categories of *de novo* genes (Figure S5).

### The pattern of correlated expression is insensitive to parameters in *de novo* gene identification

As reviewed recently, the identification of *de novo* genes faces tremendous challenges [14], [24]. We thus investigated whether the major findings we reported here, that *de novo* protein-coding genes appear to have acquired a regulated transcript structure and expression profile before acquiring coding potential, are robust and insensitive to these parameters.

Firstly, all patterns remained unchanged when stricter criteria were considered (Figure S6, S7, S8; Table S4): i) when using a more conservative list of fifteen *de novo* genes where the hominoid ORF should be 50% (instead of 30%) longer than all other out-group species, the conclusion remained the same (Figure S6, Table S4); ii) Although unlikely, it cannot be completely ruled out that a stop codon-containing exon might be spliced out, or an alternative splice site might be used to avoid a stop codon. Thus we repeated our analysis with eighteen *de novo* genes encoding only a single coding exon and found the conclusion unchanged (Figure S7, Table S4); and iii) the conclusion was unchanged when *de novo* genes partially contributed by *Alu*-elements were removed (Figure S8, Table S4) and when the sequence alignment parameters for identifying *de novo* genes were modified (Table S4). Secondly, comparative transcriptome analysis of both previously reported lists of human-specific *de novo* protein supported the main findings in our study. Specifically, among the three human-specific *de novo* genes identified previously by Knowles *et al*, two were supported by convincing RNA-Seq transcription evidence. Both were also transcribed in rhesus macaque with tissue expression profiles similar to human (Spearman correlation coefficient = 0.9 for both genes), although at slightly lower expression levels (Table S8). The recently-identified sixty human-specific *de novo* genes [5] showed a similar result: 22 of 34 genes showed lower expression levels in rhesus macaque compared with human orthologs (Figure S9). Most of these *de novo* genes had similar transcription structure and splicing junction structure in rhesus macaque (Figure S9), and significantly more genes showed correlated tissue expression profiles with human, based on *Monte Carlo* simulations neglecting orthologous relationships (*p-value* = 0.0009, Figure S9, Materials and Methods).

Our data supports that *de novo* origination rather than ORF expansion drove the origination of hominoid-specific ORFs. The ORF expansion model, which believes that a shorter ORF may predate the human-rhesus split, predicts that the longer the ORF in rhesus macaque relative to the ORF in human, the stronger the transcriptional correlation between them. The model also predicts that human *de novo* genes encoding multiple exons are more likely to be transcriptionally correlated between human and rhesus macaque, given that these genes may be generated by exon-level expansion of an ancestral protein-coding gene. However, our data showed that the ratio between the length of a human protein and that of the corresponding truncated protein in rhesus macaque did not have strong predictive power for the expressional correlation between these two species, measured by either relative expression intensity (Figure S10A, Spearman correlation coefficient: −0.16) or cross-tissue transcriptional correlation (Figure S10B, Spearman correlation coefficient: −0.38). In addition, rhesus orthologs of human *de novo* genes with multiple coding exons did not show higher transcription than orthologs of human *de novo* genes with only one exon (one-sided Fisher's exact test, *p*-value = 0.97). Both lines of evidence favor the *de novo* origination model instead of the ORF expansion model.

## Discussion

### Challenges to identify *de novo* protein-coding genes

As reviewed recently, the detection of *de novo* genes faces tremendous challenges [14], [24]. Using different bioinformatics strategies and annotation databases, the number of human-specific *de novo* proteins identified had ranged from three [7] to sixty [5]. Such a remarkable difference is largely due to different strategies used to confirm the in-group transcript structure and coding potential, verify the absence of proteins from out-group species, and confirm that the gene is in the process of *de novo* origination rather than gene loss or other gene origination mechanisms such as gene duplication [14], [24].

Here, to confirm the transcriptional and translational expression of these genes in human, we applied genome-wide filters using genome-wide RNA and protein expression data. We took advantage of the newly-assembled genomes and transcriptomes to validate the fine transcript structure of these genes. Specifically, half of the splicing junctions in these genes (twenty out of forty) were supported by at least two uniquely mapped RNA-Seq reads across the junction in our own RNA-seq data (Figure S1), and three were supported by one RNA-Seq read. When considering more public RNA-Seq datasets, additional evidence was found to support three other splicing junctions (Table S9). Considering that these newly-originated transcripts were generally expressed at low levels (Figure 1C), the likelihood that two high-quality RNA-seq reads spanned the same splicing junction by chance was low. In addition, we also required that at least one mRNA or EST sequence supported the transcript structure (Table S9). Combining evidence from spliced ESTs, mRNAs and RNA-Seq junction reads, all of the forty splicing junctions were well supported. The *de novo* genes we used in the subsequent comparative transcriptome analyses thus had a clear transcript structure with in-group coding potential.

Because it is technically and economically prohibitive to fully profile the proteomes across species, individuals, tissues and developmental stages, conceptual translation of ORFs remains the main strategy in the field to verify the absence of proteins from out-group species. However, it is difficult to conclusively say which parameter is better. The ORF length cutoff in out-group species ranged from 50% to 80% in previous studies [5], [7]. In our study, we chose 70% as the cutoff based on data from previous studies [5], [7]. In fact, 62.5% (fifteen out of 24) of the *de novo* genes conceptually encode a peptide at least 50% shorter in the out-group species than in human, based on phylogenetic distribution of “disabling” mutations.

We introduced three additional filters in our identification pipeline. First, we inferred the locus and ORF ages in the phylogenetic tree and removed genes without a convincing gene locus or ORF age assignment. We used as many high-quality out-group species as possible. For example, a human-specific *de novo* gene was defined as a gene without intact orthologous ORF in multiple out-group species including chimpanzee, orangutan, rhesus macaque, mouse, dog and so on. Second, since unambiguous sequence information in multiple out-group species was needed to trace the detailed origination process, we only retained reliable codon-based alignments with high sequence coverage (≥70%) and DNA sequence identity (≥50%). In this way, rapidly-evolving loci at the DNA level were excluded. Finally, as a stop codon-containing exon could be spliced out, or an alternative splice site could be used to avoid the stop codon, for the *de novo* genes with at least two coding exons, we manually curated the transcript structure based on RNA-Seq data and removed genes with ambiguous transcript structures in out-group species (a demo case is shown in Figure 2C). Furthermore, if the proteins were indeed absent from out-group species, we would expect that their ratio of non-synonymous substitution rate to synonymous substitution rate (dN/dS) should be close to 1 [25]. In order to test this hypothesis, we aligned the truncated forms of the human *de novo* proteins in non-human primates and calculated the merged dN/dS ratio using DnaSP [26]. Indeed, the value did not deviate significantly from 1 (dN/dS = 0.904), suggesting that these truncated forms of ORFs in non-human primates may largely not encode *bona fide* proteins.

The last question was to distinguish *de novo* gene origination from gene loss and other gene-origination mechanisms such as gene duplication. In our study, to verify that these were newly created genes in hominoid instead of dying old genes in out-group species, we manually checked the corresponding ORF regions in multiple primate out-group species. For each *de novo* gene, the phylogenetic distribution of ORF-disabling mutations in multiple primate out-group species as well as the detailed multiple sequence alignments are shown in Dataset S1 and Table S1. The existence of common ancestral disablers shared by multiple out-groups suggested that gene origination rather than gene loss was more likely for these genes (Dataset S1, Table S1). To confirm that they originated through *de novo* evolution instead of by other mechanisms such as gene duplication, we searched these human proteins against all Ensembl-annotated proteins by BLASTP, and a *de novo* gene had to have no significant paralog (BLASTP E-value≤10^−6^) in the human genome. BLASTP E-value cutoffs ranging from 10^−4^ to 10^−10^ had been used in previous identification of *de novo* genes [5], 7,12,27. In our study, we used 10^−6^ as the cutoff in the initial screening followed by two additional checks: first, in order to account for the fact that short proteins generally have a large E-value in the alignment, we also required that a *de novo* gene should not align well (coverage ≥70% and identity ≥50%) with any other human genes; and second, a human *de novo* protein should not have any paralog annotated by the Ensembl pipeline, which has been shown to be quite sensitive [28].

After removing the many possibilities that could introduce false-positives, our strategy may cause some false-negatives. However, our gene list does appear convincing as it did not miss any of the three human-specific genes reported by Knowles *et al* in the genome-wide identification of human *de novo* genes (Figure 1A). Also, the *de novo* gene dataset verified several previously proposed features of *de novo* protein-coding genes such as shorter ORFs and co-option of the neighboring transcriptional context. The main finding we proposed in this study was insensitive to the parameters used in defining *de novo* genes, as discussed in detail in the previous sections (Figures S6, S7, S8).

### Brain and testis may be favorable locations for newly created *de novo* genes

The out-of-testis hypothesis states that the testis is a hotbed of new gene origination, given its permissive chromatin state allowing widespread transcription [13], [29]. We analyzed the expression profiles of the *de novo* protein-coding genes we identified and tested the out-of-testis pattern by following the idea described in [29]. First, we investigated the relative abundance of each *de novo* gene across testis, skeletal muscle, liver, heart, adipose, prefrontal cortex and cerebellum in terms of RNA-Seq read counts. As shown in Figure 4A, the *de novo* genes were most often transcribed in cerebellum. Testis ranked number two or three, depending on the species. Second, we tested whether class I (human-specific) *de novo* genes are more often biased to testis compared to class II (human/chimpanzee shared) *de novo* genes. As discussed in [29], Class I should be more often expressed in testis if the out-of-testis hypothesis stands. As shown in Figure S4, different from this prediction, class II *de novo* genes had a much higher proportion of reads expressed in testis. Thus, *de novo* genes may not generally conform to the out-of-testis hypothesis. Testis-biased expression of *de novo* genes had been reported so far in the fruit fly [9], [12]. By contrast, none of the previously reported human-specific *de novo* genes appeared to have such a pattern: i) the three *de novo* genes identified in [7] are broadly transcribed; ii) higher expression levels of *FLJ33706* [8] were observed in several brain regions such as cerebellum or cerebral cortex compared to testis (relative expression levels, 2.4–4.9 *versus* 1.5); and iii) sixty *de novo* genes identified in [5] are most often transcribed in cerebral cortex with testis following as the second top tissue. In summary, testis may still be the location of some *de novo* genes, but brain appears to be a favorable location for the origination of many human *de novo* genes.

### Long non-coding RNAs may serve as a birth pool of protein-coding genes

Our work demonstrated how origination of *de novo* genes occurred by Darwinian step-by-step evolution as speculated in a previous study [14]. Specifically, our comparative transcriptome analysis suggests that transcription tends to occur first, since the *de novo* locus with coding potential is transcriptionally correlated with its non-coding counterparts in rhesus macaque or chimpanzee with respect to transcript structure and tissue expression profile. Such a result also suggests that the ancestral form was a functional non-coding RNA, given its regulated rather than promiscuous transcription. More interestingly, after acquiring an ORF, the transcript is subject to further transcriptional optimization as supported by higher abundance expression compared to their non-coding orthologs. Thus, a protein-coding gene is not built in a single step but by the gradual acquisition of various elements. Our work sheds new light on the challenging problem of finding the potential functions of long non-coding RNAs [30]. At least a portion of these polyadenylated long non-coding RNAs may represent a “birth pool” of protein-coding genes, especially those with active and regulated transcription. It would be interesting to delineate the functional differences between non-coding RNAs and their orthologous *de novo* protein-coding genes, especially since the origination of protein-coding potential and further optimized transcription might render the protein-coding version more functional.

## Materials and Methods

### Ethics statement

Rhesus macaque tissue samples were obtained from the internationally-accredited (Association for Assessment and Accreditation of Laboratory Animal Care, AAALAC) animal facility of the Institute of Molecular Medicine in Peking University. The present study was approved by the Institutional Animal Care and Use Committee of Peking University. All animals were handled in strict accordance with good animal practice as defined by the relevant national and local animal welfare bodies.

### Dating protein-coding genes in the phylogenetic tree

Genome-alignment-based pipelines were developed to infer the origination time of a given genomic region by modifying a previous gene-alignment-based method [31], [32]. Briefly, UCSC netted chained files [33] were analyzed to verify whether a given locus had a reciprocal syntenic alignment in the out-group genomes. Multiple out-group species were scanned to control the false discovery rate raised by occasional sequencing gaps. A specific branch was then assigned to this locus by following the rule of parsimony. As we investigated whether a best-to-best match could be found between a locus and out-group loci regardless of chromosomal linkage information, orthologous genes could be identified independent of gene annotation of out-groups and robust with different chromosomal locations due to gene fusions or translocations [32].

### Genome-wide identification of hominoid-specific *de novo* protein-coding genes

We developed a sophisticated pipeline to identify hominoid-specific *de novo* protein-coding genes [7], [8]. First, for each locus, the existence of the ORF in multiple out-group species (chimpanzee, orangutan, rhesus macaque, mouse, guinea pig, dog, hedgehog and armadillo) was inferred separately. Specifically, on the basis of the gene locus age assignment described in the previous section, if the locus was absent from one out-group, we directly classified this case as the ORF not existing in this out-group neither. Otherwise, the fine-scale gene structure in this out-group was inferred by Exonerate [34], using human Ensembl proteins [35] as reference. For the codon-based pairwise alignment generated by Exonerate, we retained those that were reliable and had ≥70% coverage and ≥50% identity. If the alignment generated by Exonerate failed to pass these criteria, we classified such a scenario as ambiguous, namely, we did not know whether the orthologous ORF existed or not. Next, if the sequence in this out-group encoded at least one ORF disabler, such as frame-disrupting indels or premature stop codons, and the subsequent maximum continuous peptide was shorter than 70% of the human ORF length, the ORF was inferred to be non-existent in this out-group. To ensure the absence of coding potential in this out-group, we also required that the Ensembl automatic annotation [35] did not identify any homolog in this out-group to avoid potential misalignments by Exonerate [34]. The exhaustive search functionality of Exonerate was enabled to conservatively identify species- or lineage-specific *de novo* genes (Figure 1A).

We then inferred the origination timing of ORFs for these *de novo* genes by summing up the presence or absence information in multiple out-group species, along the phylogenetic tree with the rule of parsimony. As the genomic assembly of non-human species is generally error-prone, the existence information for orthologous ORFs in multiple out-group species was used to avoid the identification of false-positive frame disruption potentially caused by genome sequencing errors, *e.g.* one human-specific ORF was defined as not existing in chimpanzee, orangutan, rhesus macaque, mouse, guinea pig, dog, hedgehog and armadillo. We discarded those cases with contradictory information in several out-group species.

We further searched these human proteins against all Ensembl-annotated proteins by BLASTP to verify that they originated through *de novo* evolution, instead of other gene origination mechanism such as gene duplication. A *de novo* gene was defined only if there were no other hits passing the BLAST E-value cutoff of 10^−6^ in human Ensembl proteins and no annotated paralogs by Ensembl [35]. Finally, only hominoid-specific *de novo* genes without coding potential in rhesus macaque were kept for further manual curation (Figure 1A). The initial gene list covered all four human-specific *de novo* genes reported in previous studies [7], [8], as well as five of the human-specific *de novo* genes reported in a more recent study [5] using a relatively lenient criterion [24]. Ensembl [35] release 51 was downloaded as the basic gene dataset for this analysis. We used MySQL to organize the data, BioPerl [36] and BioEnsembl [37] to fold the pipeline, and R (v2.13.1) to perform all statistical analyses.

### Inclusion criteria for hominoid-specific *de novo* protein-coding genes

We manually curated the initial gene list to identify convincing hominoid-specific *de novo* protein-coding genes for subsequent statistical analyses.

First, we introduced genome-wide filters such as genome-wide RNA and protein expression data to make sure these genes had convincing evidence for transcriptional and translational expression in human. Public RNA-Seq data from nine human tissues were analyzed to estimate the gene expression level of each *de novo* gene, following the protocols in the original report [21]. Peptide evidence from large-scale mass spectrometry studies was further extracted from the proteomics identifications database (PRIDE) [38] and the PeptideAtlas project [39]. Briefly, all human peptide sequence data were downloaded and all-against-all BLAST similarity searches were performed. A peptide was considered to show convincing peptide evidence for a *de novo* gene only if its whole sequence completely and identically matched the CDS region, with the second-best hit in the genome (if existing) including at least two mismatches (Ensembl release 51 annotation). Only genes with i) RNA-Seq RPKM >0.5 in at least one of the nine human tissues, and ii) at least one convincing item of peptide evidence in support, were retained (Figure 1A).

Second, besides the EST and mRNA evidence used in the initial genome-wide pipeline, in the manual curation, we also took advantage of newly-assembled genomes and transcriptomes to validate the fine-scale transcript structure of these genes in human and out-group species (Figure 1A). Genes with the stop codon-containing exon spliced out, or a totally different splicing structure in out-group species were discarded (Figure 2C).

Third, to verify that these genes were newly created instead of old dying genes, we manually checked the corresponding ORF regions in multiple primate out-groups in the context of the revised transcript structure, to ensure the existence of common ancestral disablers shared by multiple out-group species. Genes without common ancestral disablers were not included in the subsequent statistical analyses (Figure 1A). For each *de novo* gene, the phylogenetic distribution of ORF-disabling mutations in multiple primate out-groups, as well as the detailed multiple sequence alignments are shown (Dataset S1, Table S1). Newly-assembled genomes and transcriptomes from the UCSC genome browser annotations [33] were also considered in the manual process to ensure the gene structure, locus age assignment and ORF integrity.

Overall, 24 hominoid-specific *de novo* genes were identified, with unambiguous gene structures and confirmed locus and ORF age assignment (Figure 1A). As no arbitrary criteria were included in the manual curation process, the *de novo* gene dataset is not biased with respect to age dating and transcription profiles.

### Characteristics of *de novo* protein-coding genes

The basic characteristics of the *de novo* genes and background genes were downloaded from UCSC [33], Ensembl [35] and Swiss-Prot [40]. R scripts (v2.13.1) were implemented to perform all statistical analyses and calculate the distributions of these characteristics for *de novo* genes, using the human genome as the background. For each human-specific *de novo* gene, we aligned the truncated form of the human ORF between chimpanzee and orangutan, and calculated the merged dN/dS score with DnaSP [26].

### Library preparation and strand-specific poly (A)–positive RNA–Seq

Total RNA was extracted from five rhesus macaque tissues using the Trizol method and analyzed by an Agilent 2100 bio-analyzer (Agilent Technologies). Poly (A)-positive RNA was purified with the Dynabeads mRNA purification kit (Invitrogen) from 5 µg of total RNA with high quality (RNA integrity number ≥7.5), following the manufacturer's instructions. A strand-specific RNA-Seq library preparation on the basis of the incorporation of deoxy-UTP was performed as reported previously [41]. Briefly, after the first-strand cDNA synthesis, non-incorporated nucleotides were removed and dTTP was substituted by dUTP during the synthesis of the second strand. Then after ligation with a Y-shaped adaptor, the deoxyuridine-containing strand was selectively removed with UNG, leaving only the first cDNA strand [41]. Amplified material was loaded onto a flow-cell and sequencing was carried out on the Illumina HiSeq2000 platform by running 90 cycles (paired-end design) according to the manufacturer's instructions.

### Computational processing of RNA–Seq data

In-house paired-end mRNA sequence tags were mapped to the rhesus macaque genome (rheMac2) by TopHat (v1.2.0) [42]. Multiple alignment reads were discarded. The RPKM scores for each gene were calculated as previous described [43]. FastQC (v0.10.0) and a series of Perl (v5.12.2) and R (v2.13.1) scripts were implemented to evaluate the quality of the RNA-Seq data, including i) the average quality of reads, ii) the evenness of short-read distributions on transcripts, iii) the contrasts of read distributions in exon, intron and intergenic regions, iv) the efficiency of strand-specific strategy, v) mutation rates across the reads, and vi) correlations of RPKM scores with real-time PCR results, by calculating the Spearman correlation coefficient across 877 transcripts as measured by Taqman Gene Expression Assays and by strand-specific RNA-Seq read counts [21]. Representative regions for each *de novo* gene-encoded transcript were selected, which met the criteria of i) being located in the exonic regions of *de novo* genes, ii) having no overlap with other known genes, and iii) accurate identification of syntenic regions in rhesus macaque and chimpanzee genomes based on UCSC genomic alignment. The RPKM scores for each *de novo* gene were calculated for the representative regions. Public RNA-Seq datasets for human [20], [21], rhesus macaque [20] and chimpanzee tissues [20], [22] were downloaded from SRA and mapped to the human (hg18), chimpanzee (panTro2) or rhesus genome (rheMac2) respectively with similar pipelines. Public strand non-specific RNA-Seq data meeting the inclusion criteria were further integrated, in which i) the RNA-Seq study was performed in tissues derived from male individuals; ii) the RNA-Seq library was generated using high-quality RNA samples (RIN >7.0); and iii) the RNA-Seq reads were mapped to the genome with high mapping rates (uniquely mapped reads >40%). Similarly, for the orthologous loci of each *de novo* gene, RPKM scores were calculated for the representative regions as noted above and an RPKM cutoff of 0.2 was set for convincing transcription, a value significantly higher than that in intergenic region (*Monte Carlo p-value*<0.05, Figure S2).

Uniquely mapped RNA-Seq reads covering splicing junctions were extracted by Samtools (v 0.1.16) [44]. The correlation coefficient between the RPKM scores in human tissues and the parallel rhesus macaque tissues were calculated using R scripts (v2.13.1) and a correlation coefficient of 0.5 was used as the cutoff for positive correlations between tissue expression profiles. 10,000 *Monte Carlo* simulations were performed to generate the distribution of correlation coefficient scores, under the assumption of neglecting ortholog relationships between the tissue expression profiles in human and rhesus macaque, while keeping all other parameters identical to those used for the real data. Statistical analyses were further performed using this distribution as background and *Monte Carlo p-values*<0.05 were considered significant.

Our RNA-Seq data was of high quality after extensive evaluation. Specifically, the mean PHRED quality score was >30 across the whole reads (one possible sequencing error per 1000 bases, Figure S1A). The even distribution of short reads on transcripts further revealed well-controlled randomized fragmentation of the transcripts in the RNA-Seq experiments (Figure S1B). The strand-specific strategy worked well since the reads with correct strand information were over 100-fold more than strand-mislabeled reads (Figure S1C). As expected, RNA-Seq read counts showed very sharp peaks at exonic regions, with a read density >100-fold higher than that in introns or intergenic regions, revealing that most reads derived from mature polyadenylated RNAs (Figure S1D). The percentage of mis-mapped reads across the genome was <0.1%, as estimated using a method previously reported [21]. The average mutation rate was as low as 1.51 errors per read, with mutations evenly distributed across the reads (Figure S1E). Two standard human RNA samples, from Brain Reference and Universal Human Reference (UHR) [21], were then quantified using the identical experimental and computational pipelines, and obtained highly consistent results with Taqman-based real-time PCR quantification (Spearman correlation coefficient = 0.94) (Figure S1F). The data also made it possible to accurately assemble the gene structures of *de novo* genes with complex transcriptional contexts and infer the representative regions as described. In summary, high-quality RNA-Seq studies were performed in five rhesus tissues, with accurate strand information and transcript expression quantification (Figure 2, Figure S1).

## Supporting Information

Dataset S1 Multiple sequence alignments for *de novo* genes.
(PDF)

Figure S1 Strand-specific mRNA-Seq analysis in five rhesus tissues reveals comprehensive transcriptome information for rhesus macaque. (A) For each position of the reads, PHRED quality scores across all reads were calculated and summarized. The central red line is the median value, the yellow box represents the inter-quartile range (25–75%), the upper and lower lines represent the 10% and 90% points, and the blue line represents the mean quality. (B) Distribution of short reads on transcripts. The even distribution reveals well-controlled randomized fragmentation of the transcripts in the RNA-Seq experiments. Data are shown as mean ± SD. (C) Evaluation of strand-specific strategy. The reads with correct strand information were >100-fold more than strand-mislabeled reads for all five tissues from rhesus macaque. (D) RPKM scores of exonic regions, intronic regions and intergenic regions, in five rhesus tissues. (E) For each position of the reads, mutation rates were calculated and summarized. The average mutation rate was 1.51 errors per read. Data are shown as mean ± S.D. (F) Scatter plot showing relative expression of 877 transcripts between reference brain (HBRR) and UHR sample (UHRR) as measured by Taqman Gene Expression Assays (UHRR_qPCR/HBRR_qPCR) and by strand-specific RNA-Seq read counts (UHRR_RNA-Seq/HBRR_RNA-Seq). The Spearman correlation coefficient was calculated and is shown with the linear regression curve (R = 0.945).
(PDF)

Figure S2 Estimation of RPKM scores for the genomic background represented by intergenic regions. 10,000 intergenic regions were randomly selected and used to calculate RPKM scores from seven human tissues (adipose, skeletal muscle, prefrontal cortex, cerebellum, heart, liver, testis; S2-1), five chimpanzee tissues (prefrontal cortex, cerebellum, heart, liver, testis; S2-2) and seven rhesus macaque tissues (adipose, skeletal muscle, prefrontal cortex, cerebellum, heart, liver, testis; S2-3). For each tissue type, the distribution of RPKM scores is illustrated and the number of regions with a score >0.2 were counted, which was further used to estimate *p-values* for the genomic background transcription with an RPKM cutoff of 0.2.
(PDF)

Figure S3 Sequence motif flanking the splicing junctions in chimpanzee and rhesus macaque. Sequence motifs near both the donor site and acceptor site, summarized by splicing junctions in *de novo* genes in chimpanzee (S3-1) and rhesus macaque (S3-2).
(PDF)

Figure S4 Tissue-enriched expression of *de novo* genes in human. RNA-Seq read distribution for class I and class II *de novo* genes, using the proportion of the RNA-Seq library of seven tissues as background.
(PDF)

Figure S5 Non-coding orthologs of human *de novo* protein-coding genes show tissue expression profiles similar to human. For each pair of tissues, Spearman correlation coefficients were computed separately and the extent of tissue-specific differences in *de novo* gene expression are shown for Class I (S5-1) and Class II genes (S5-2). Comparisons between pairs of corresponding tissues in different species are highlighted with dotted lines.
(PDF)

Figure S6 *De novo* protein-coding genes with stricter out-group ORF length cutoff encoded non-coding RNAs in the rhesus macaque with a correlated tissue expression profile and lower expression level. (A) Summed RPKM scores (log_2_ transformed) of *de novo* genes in seven tissues from human and rhesus macaque. The human genes are ordered with decreasing expression levels as reference, and genes in rhesus macaque are aligned accordingly. (B) Correlation coefficients for tissue expression profiles between human and rhesus macaque. The real data for *de novo* genes (brown histograms) are illustrated with background simulated by 10,000 *Monte Carlo* simulations neglecting ortholog relationship for the tissue expression profile. (C) For each pair of tissues, Spearman correlation coefficients were computed separately and the extent of tissue-specific differences in *de novo* gene expressions are shown.
(PDF)

Figure S7 *De novo* protein-coding genes with single coding exon encoded non-coding RNAs in the rhesus macaque with a correlated tissue expression profile and lower expression level. (A) Summed RPKM scores (log_2_ transformed) of *de novo* genes in seven tissues from human and rhesus macaque. The human genes are ordered with decreasing expression levels as a reference, and the rhesus genes are aligned accordingly. (B) Correlation coefficients for tissue expression profiles between human and rhesus macaque. The real data for *de novo* genes (brown histograms) are illustrated with background simulated by 10,000 *Monte Carlo* simulations neglecting ortholog relationship for the tissue expression profile. (C) For each pair of tissues, Spearman correlation coefficients were computed separately and the extent of tissue-specific differences in *de novo* gene expression are shown.
(PDF)

Figure S8 *De novo* protein-coding genes without *Alu*-element encoded non-coding RNAs in rhesus macaque with a correlated tissue expression profile and lower expression level. (A) Summed RPKM scores (log_2_ transformed) of *de novo* genes in seven tissues from human and rhesus macaque. The human genes are ordered with decreasing expression levels as a reference, and the rhesus genes are aligned accordingly. (B) Correlation coefficients for tissue expression profiles between human and rhesus macaque. The real data for *de novo* genes (brown histograms) are illustrated with background simulated by 10,000 *Monte Carlo* simulations neglecting ortholog relationship for the tissue expression profile. (C) For each pair of tissues, Spearman correlation coefficients were computed separately and the extent of tissue-specific differences in *de novo* gene expression are shown.
(PDF)

Figure S9 Human-specific *de novo* protein-coding genes (identified by Wu *et al*) encoded non-coding RNAs in rhesus macaque with fixed transcript structure and correlated tissue expression profile. (A) For each *de novo* gene, the base-level density of RNA-Seq reads across the transcript (red), as well as the upstream/downstream regions (grey, 50% of the length of the transcript), are shown. The raw density scores computed from RNA-Seq read coverage were normalized to the total reads across the region. (B) Summed RPKM scores (log_2_ transformed) of 34 *de novo* genes in five tissues from human and rhesus macaque. The human genes are ordered and connected with decreasing expression levels as a reference, and the rhesus macaque genes are aligned accordingly. The thinner lines indicate a two-fold change of expression, compared with the reference expression level. (C) Correlation coefficients for tissue expression profiles between human and rhesus macaque. The real data for the 28 *de novo* genes (brown histograms) are illustrated with background simulated by 10,000 *Monte Carlo* simulations neglecting ortholog relationship for the tissue expression profile (blue histograms).
(PDF)

Figure S10 ORF expansion size has no correlation with ancestral transcription activity and tissue expression profile. For each *de novo* gene, the “Ratio of ORF Length” was calculated by normalizing the size of the truncated ORF in rhesus macaque to the size of the intact ORF in human. The “Relative Expression Level” was further determined as the ratio of summed expression in seven macaque tissues to that of corresponding human tissues. The Spearman correlation coefficient between human and rhesus macaque tissue expression profiles were calculated as before. The correlations between the “Ratio of ORF Length” and “Relative Expression Level” (A), as well as “Expression Profile Correlation Coefficient” (B) are shown in scatter diagrams.
(PDF)

Table S1 The phylogenetic distribution of ORF “disabling” mutations in multiple primate out-groups for *de novo* genes.
(PDF)

Table S2 Basic information for *de novo* genes.
(PDF)

Table S3 Expression evidence for *de novo* genes.
(PDF)

Table S4 *De novo* genes with different parameters in the computational identification.
(PDF)

Table S5 *De novo* genes partially contributed by *Alu* elements.
(PDF)

Table S6 Representative regions selected to calculate the expression levels of *de novo* genes.
(PDF)

Table S7 Statistics of public RNA-Seq data integrated in this study.
(PDF)

Table S8 Expression of *de novo* genes in human and rhesus macaque.
(PDF)

Table S9 Human splicing junctions supported by expression evidence.
(PDF)

Table S10 Chimpanzee splicing junctions supported by RNA-Seq reads.
(PDF)

Table S11 Monkey splicing junctions supported by RNA-Seq reads.
(PDF)
