# Supplementary material for: Hominoid-Specific De Novo Protein-Coding Genes Originating from Long Non-Coding RNAs
Source: PLoS Genet. 2012 Sep 13;8(9):e1002942. doi: 10.1371/journal.pgen.1002942 (PMC3441637; doi:10.1371/journal.pgen.1002942)
Supplement: Dataset S1 — Multiple sequence alignments for de novo genes. (PDF) [file pgen.1002942.s001.pdf]

Dataset S1. Multiple sequence alignments for *de novo* genes. Alignments of 24 *de novo* genes for human, chimp, gorilla, orangutan, rhesus, baboon and marmoset sequences. Common disablers were marked in red rectangles. "-": gap; "N": unknown base with low quality; "IIIII": long insertions with at least 200 base pairs; "-----" in the same position of all species: position of splicing junction.

S1-1: ENSP00000273641 ENST00000273641 ENSG00000145063 H

```
Human      ATGGGATTACTGAGTCAGAGGAAGTGGACATTGAGTGGCTCTCAACAGACAG-GCTGTGTTGCTCTCACAGTGCCCA----G-----CTTCCCATGGGTG
Chimp      ATGGGATTACTGAGTCAGAGGAAGTGGACATTGAGTGGCTCTCAACAGACAG-GCTGTGTTGCTCTCACAGTGCCCA----G-----CTTCCCATGGGTG
Orangutan  ATGGGATTACTGAGTCAGAGGAAGTGGACATTGAGTGGCTGTCAACAGACAG-GC------ACTCTCACAGTGCCCA----G-----CTTCCCATGGGTG
Rhesus     ATGGGATTACTGAGTCAGAGGAAGTGGACGTTGAGTGGCTTTCAACAGACAG-GCTGTGTTGCTCTCATGGTGTCCATGCAG-----CCTCCCATGGGTG
Marmoset   ATGGGATTACTGAGTCAGCGGAAATGGACACTGAGTGCCTTTCAATAGACAGAGTCGTGTTGCTCTCA--GTGCCCATGCAT-----CATCCCACGCGTG

Human      GCCAGCCGAATGCATTATGGGAGAAAGCAGGTCTCGTGGATAATATTTCTGAAGATCGGAGCAGGTTGC---CAGGTCCACGTCGGCCATGACTGCAGCA
Chimp      GCCAGCCGAATGCATTATGGGAGAAAGCAGGTCTCGTGGATAATATTTCTGAAGATCAGAGCAGGTTGTGT-CGGGTCCACGTTGGCCATGACTGCAGCA
Orangutan  GTCAGCCGAATGCATTTTGGGAGAAAGCAGGTCTCGTGGATAATATTTCTGAAGATCAGAGCAGGTTGTGT-CGGGTCAACGTTGGCCACGACTGCAGCA
Rhesus     GGCAGCCGAATGCGTTTTGGGAGAAAGCAGGTCTTGTGGATAATATTTCTGAAGATCGGAGCAGGTTGTGTCCAAGTCCATGTTGGCCATGACTGCAGAA
Marmoset   GGCAGCCGAATGCATTTTGGG-GAAAGCAGGTCTCCTGGGTAATATTTCTGAAGATCGAAGCAGGTTGTATTTAAGTCCACGTTGGCCAAGACTGCAGCA

Human      CGCTCCGGAGGCAGCAGGGGGCACCCTGGAGCTTCGCATCCTCCTTCAGGCCCGCGGCATCTCCCCTGGCTCCACCATCCCCAGGTG-------------
Chimp      TGCTCCGGAGGCAGCAGGGGGCACCCTGGAGCTTCGCATCCTCCCTCAGGCCCGCGGCATCTCCCCTGGCTCCACCATCCCCAGGTG-------------
Orangutan  CGCTCCGGAGGCAGCAGGGGGCACCCTGGAGCTTCGCATCCTCCCTCAGGCCCGCGGCTTCTGCCCTGGCTCCACCATCCCCAGGCG-------------
Rhesus     CGCCCCAGAGGCAGCAGGGGGCACCCTGGAGCTTTGTATCCTCCCTCCGACCCGCAGCATCTCCCCTGGCTCCACCATCCCCAGGTGCCCAGAAGCCGG-
Marmoset   CGCTCCGGAGGCAGCAGAGGGCAGCCTGGAGCTTCGCTTCCTCCCTCAGGCCCGCGGCAGCTCCC-TGGCTTCACCATCCCCAGGTGCAGACAAGCCGGT

Human      --------TCTCAG-----GCCT--GTTTCCTCCTCATGAGAGGTGGAGCGGTGGTTCCCAAACACGATGTGGGAAGTGTGTTAAAATGCAGAT-CCTGG
Chimp      --------TCTCAG-----GCCT--GTTTCCTCCTCATGAGAGGTGGAGCAGTGGTTCCCAAACATGATGTGGGAAGTGTGTTAAAATGCAGAT-CCTGG
Orangutan  --------TCTCAG-----GCCTCAGTTTCCTCCTCATGAGAGGTGGAGCAGTGGTTCCCAAACATGACATTGAAAGTGTG-----------AT-CCTGG
Rhesus     GT-----GTCTCAG-----GCCTCAGTTTCCTCCTCATGAGAGGTGGAGCAGTGGTTCCCAAACATGATGTTGGAAGTGTGTTAAAATGCAGATTCCTGG
Marmoset   GTCGAAGGTCTGAG-----GCCTCAGTTTCCTCCTCATGAGAGGTGGAGCAGTGGCTCCCAAATCTCACACTGGAAGTGTGTTAAAATTCAGATTCCTGG

Human      GCTCGACCCTGAAGCTCTTCCGGCATCCACCTTCCCAGGTGACCAGACTCTGGGGAAGGCATCACCTCAAGACTCCTGCACCCTTCCTCCAGAGTCCTGG
Chimp      GCTCGACTCTGAAGCTCTCCCAGCATCCACCTTCCCAGGTGACCAGACTCTTGGGAAGGCATCACCTCAAGACTCCTGCACCCTTCCTCCAGAGTCCTGG
Orangutan  GCCTGACTCTGAAGCTCTTCCAGCATCCACCTTCCCAAGTGACCAGACTCTTGGGAAGGCATCACCTCAAGATCTCTGCACCCTTCCTCCAGAGTCCTGG
Rhesus     GCTCGACTCTGAAGCTCTTCCAGCAGCCACCTTCCCAGGTGACCAGACCCTTGGGAAGGCATCACCTCAAGACCCCTGCAGAATTCCTCCAGAGTTCTGG
Marmoset   ACTCGACTCTGAAGCTCCTCCAGCATCTACCTCCCCAGGTGACCAGACCCATGGGAAGGCATCACCTCAAGACCCCTGCACCCTTCCTCCAGAGTCCTGG

Human      GATTCAGCTAAATCCTGGGAAG-----GTGCCT-----------GCCTCCCTGCTGCGCCTGGCCACGTGGAAACCCCTGTAG
Chimp      GATTAAGCTAAATCCTGGGAAG-----GTGCCTGCCTCCCTTGGGCCTCCCTGCTGCACCTGGCCACGTGGAAACCCCTGTAG
Orangutan  GGTTAAGCTAAGTTCTGGGAAG-----GTGCCTGCCTCCCTTGGGCCTCCGTGCTGCGCCTGGCCATGTGGAAACCCCTGTAG
Rhesus     GATTAAGCTAAGTCCTGGGAAG-----GTGCCTGCCTGCCTTGAGCCTCCCTGCTGCACCTGGCCATGTGGAAACCCCTGTAG
Marmoset   GATTAAGCTGAGTCCTGGGAAG----------------------GCCTCCCTGCTGCGCCCGGCCACATGGAAACCCCTGTAG
```

S1-2: ENSP00000308330 ENST00000308946 ENSG00000172927 H

```
Human        ATGGCCCTCAGAATCTGCGTCACATACACCCCAGCTCT-CCCGATAGGTCTCTGCACTCGCTGTTGCCTCTGCCTGGAACAGTCTCCCTCCTGGTGTCAT
Chimp        GTGGCCCTCAGAATCTGCGTCACATACACCCCAGCTCT-CCCGATAGGTCTCTGCACTCGCTGTTGCCTCTGCCTGGAACAGTCTCCCTCCTGGTGTCAT
Orangutan    GTGGCCCTTAGGATCTGCGTCACACACACCCCAGCTCT-CCCGATGGGTCTCTGCACTCGCTGTTGCCTCTGCCTGGAACAGTCTCCCTCCTGGTGTCAT
Rhesus       GTGGCCCTCAGGGTCCGCGTCACACACACCCCAGCTCT-CGTGACGGGTCTCTGCACTCGCTGTTGCCTCCGCCTGGAACAGTCTTCCTCCTGGTGTCAT
Marmoset     ATGGCCCTCAAGGTCTGCGTCACCCACACCCCAGCTCTCCCCGCTGGGTCTC-ACACTCGCTGGTCCCTCTGCCTGGGACCGTCTCCCTCCTGGTGTCAT

Human        TGTCTCCGTGGTGTGTCCTTCCTGACCTTCCACCTCCACCAG-----TCTGTCCCCCTTGGGGACAGGGACTCGTTGCTCATGTTCACCCGGCAGGCTGG
Chimp        TGTCTCCGTGGTGTGTCCTTCCTGACCTTCCACCTCCACCAG-----TCTGTCCCCCTTGGGGACAGGGACTCGTTGCTCATGTTCACCCGGCAGGCTGG
Orangutan    CGTCTCCATGGTGTGTCCTTCCTGACTTTCCACCTCCACCAG-----TCTGTTCCCCTTGGGGACAGGAACTGGTCACTCATGTTCATCCGGCAGGCTGG
Rhesus       CATCTCCGTGGTGCGTCCTTCCTGACCTTCCACCTCCACCAG-----TCTGACCCCACCGGGGACAGGGACTGGTCACTCATGTTCATCCGGCAGACCAG
Marmoset     C-TCTCCGTGGTGCGTCCTTCCTGGCTCTGCA-CTGCACCAG-----TCTGTCCCCTCTGAATTCAGGAACTGGTCAC-CGTTTTCACCTGGTAGGCTGG

Human        ACACTTCGTGGAGGGCTCCAAAGCCGGCAGATCCCGGGGCCGCCTCTGTCTCTCCCAGGCCCTGCGTGTTGCGGTGAGAGGAGCATTTGTGTCTCTGTGG
Chimp        ACGCTTCATGGAGGGCTCCAAAGCTGGCAGATCCCGGGGCCGCCTCTGTCTCTCCCAGGCCCTGCGTGTTACGGTGAGAGGAGCATTTGTGTCTCTGTGG
Orangutan    ATGCTTCGTGGAGGGCTCCAAAGCTGGCAGACCCCGGGGCCTCCTCTGTCTCTCCCAGGCCCTGCGTGTTGCGGTGAGAGGAGCATTTGTGTCTCTGTGG
Rhesus       ACACTTCGTGGAGGGCTCCAAAGCTGGCACATCCCGGGGCCTCCTCTCTCTCTCCCAGGCCCTGTGTGTTGCGGTGAGAGGAGCATTTGTGTCTCTGTGG
Marmoset     ACGCTTCGTGGAGGGCTCCAAAGCTGGCCGATCCT-GGGCTTCCTCCGTTTCTCCCAGACCCCGCGTGTTGCAGTGAGAGGAGCATTTGTGTCTCTGTGG

Human        TTTGCTGCTGGAGCTGGTGACCGGGAGAGAAACAAGG--GAG-ACAAGGGTGCCCAGACAGGTGCGGGGCTCAGCCAGGAGGCAGAAGACGTGGACGTGT
Chimp        TTTGCTGCTGGAACTGGTGACCGGGAGAGAAACAAGG--GAG-ACAAGGGTGTCCAGACAGGTGTGGGGCTCAGCCAGGAGGCAGAAGACGTGGACGTGT
Orangutan    TTTGCTGCCGGAGCTGGTGACTGGGAGAGAAACAAGGGAGAG-ACAAGGGTGTCCAGACAGGTGCGGGGCTCAGCCAGGAGGCAGAAGACACGGACGTGT
Rhesus       TTTGCTGCTGGAGCTAATGACCTGGAGAGAAACAAGG--GAG-ACAAGGGTGTCCAGACAGGTGCGGGGCTCAGCCAGGAGGCAGAAGACGTGGATGTGT
Marmoset     TTTGCTGCTGAAGCTGATGACC-AGAGAGAAGCAAGG--GAG-ACAAGCCTCTCCAGACAGGTGTGGGGCTCAGCCAGGAGGCAGGAGACACAGACCTGC

Human        CCCGGGCCAGGAGGGTCACAGATGCACCACAAGGCACTCTGTGTGGCACTGGGAACAGGAATTCTGGGAGTCAGTCTGCAAGGGTGGTGGGCGTTGC---
Chimp        CCCGGGCCAGGAGGGTCACAGATGCACCACAAGGCACTCTGTGTGGCACTGGGAACAGGAATTCTGGGAGTCAGTCTGCAAGGGCGGTGGGCATTGC---
Orangutan    CCCGGGCCAGGAGGGTCACAGATGCACCACCAGGTACTCTGTGTGGCACTGGGAACAGGAATTCTGGGAGTCAGTCTGCAAGGGCGATGGGCATTGC---
Rhesus       CCCAGGCCA--------------GCGCCACCAGGCACTCTGTGTGGCACTGGGAACAGGAATTCTGAGAGTTAGTCTGTAAGGGTGGCGGGCATTGC---
Marmoset     CCCGGGCCAGGCAGGTCACAGATGCACCACCAGGCACTCTGTGTGGCACTGGGCACAGGAATTCTGGAATTCAATCTGCAAGGGTGGTAGGTGTTGGTGG

Human        TCACCTGGGAGAAGCCTTTAGAGTGGGCGTTGAGCAGGCCATTAGCTCGTGCCCTGAGGAGGTGCATGGGCGGCATGGGCTCTCCATGGAAATTATGTGG
Chimp        TCACCTGGGAGAAGCCTTTAGAGTGGGCGTTGAGCAGGCCATTAGCTCGTGCCCTGAGGAGGTGCATGGGCGGCATGGGCTCTCCATGGAAATTATGTGG
Orangutan    TCACCTGGGAGAAGCCTTTAGGGTGGGCGTTGAGCAGGCCATTAGCTCGTGCCCGGAGGAGGTGCATGGGCGGCATGGGCTCTCCACGGAAATTACGTGG
Rhesus       TCCCCTGGGAGAAGCCTTTCCAGTGGGCGCTGAGCAGGCCATTAGCTCGTGTCCTGAGGAGGTGCATGGGTGGCCTGGGGTCTCCACGGAAATTATGTGG
Marmoset     TCCCCTGGGAGAAGCCTTTTGAGTGGGCGTTGAGCAGGCCGGTATCTCATGCCCTGAGGAGGTGCATGGGCGGCATAGGCTTTCCACAGAAATTACGTGG

Human        GCGCGAATGGATGTGGCTCTGCGCTCACCTGGGCGAGGACTTCTGGCCGGTGCCGGGGCACTCTGCATGACCCTGGCAGAATCGAGC-TGCCCTGACTAT
Chimp        GCGCGAATGGATGTGGCTCTGCGCTCACCTGGGCGAGGACTTCTGGCCGGTGCCGGGGCACTCTGCATGACCCTGGCAGAATCGAGC-TGCCCTGACTAT
Orangutan    GTGCGCATGGATGTGGCTCTGTGCTCACCTGGGTGAGGATTTCTGGCCGGTGCCAGGGCACTCTGCATGACCCTGGCAGAATTGAGC-TGCCCTGACTGT
Rhesus       GCGCGCATGGGTGTGGCTCTGTGTTCACGTGGGCGAGGACTTCTGACCGGTGCCAGGGCACTCTGCATGACCCTGGCAGAATCAAGC-TCTCCCGACTGT
Marmoset     GTATGCATGGGTGTGGCTCTGCGCTCACCTGGGGGAGGACTTCTGGCTGGTGCTGGGGCGCTCTGTGTGACCCTGGCAGAATGGAGCTTCCCCTGAC--T

Human        GAAAGGGGAAGAAGAGCATGCC-----TGACCCTCCACC-----GGCACCCCA-CCCCTCACTGCTCCACCTGGGGCCTGCCTCTGCGGGTGGCTGGGTC
Chimp        GAAAGGGGAAGAAGAGCATGCC-----TGACCCCCCACC-----GGCACCCCACCCCCTCACTGTTCCACCTGGGGCCTGCCTCTGCGGGTGGCTGGGTC
Orangutan    GAAAGGGGAAGAAGAGCATGCC-----TGACCCCCCACC-----GGCACCCCA-CCCCTCACTGCTCCACCTGGGGCCTGCCTCTGGGGGTGGCTGGATC
Rhesus       GGAAGGGGAAGAAGAACATGCG-----TGACCCCCCACC-----AGCACCGCA-CCCCTCACTGCTCCACCTGGGG-CTGCCTCTGGGGGTGGCTGGGTC
Marmoset     GAAAGAGGAAGAAGAGCATGCCIIIIITGAAACCCCGTCIIIIIGGCACCCCA-CCCTGAACTACTCCACCTGGGGACTGCCTCTGGGGGTGGCGGGGTT

Human        CTGGCTGACTGTTGTGACTGTTGAGGCCCTGGG-GGGGTGGCGCATGGGAGTTAGGAGGACTGGCCAGGTGGGGCCCACTATGCACCCACCCCCAGTGTC
Chimp        CTGGCTGACTGTTGTGACTGTTGAGGCCCTGGG-GGGGTGGCGCATGGAAGTTAGGAGGACTGGCCAGGTGGGGCCCACTATGCACCCACCCCCAGTGTC
Orangutan    CTGGCTGACTGTTGCGACTGTTGAGGCCCTGGG-GGGGTGGGGCATGGGAGTTAGAAGGACTGGCCAGGTGGGGCCCACTATGCACCCACCCCCAGTGTC
Rhesus       CTGGCTGACTGTTGTGACAGTTGAGGCCAGGGGTGGGGTGGGGCATGGGAGTTAAGAGGACTGGCCAGGTGGGGCACACCGTGTGCCCAGCCCCAGTGTC
Marmoset     CTGGCTGACTGCTGAGGCCCTGGGGG---TGTG-GGTGTGGGGCATGGGACTTAGGAGGACTGGCCAGGTGGGGCCCACCACATGCCCACCCCCAGTGTC

Human        AGGTGCTTCTCCTCTCCTCCTCCACCACCTCCTCCTCCTCCTCCTCATCATCATCCTCACTTGTTGA
Chimp        AGGTGCTTCTCCTCTCCTCCTCCACCACCTCCTCCTCCTCCTCCTCATCATCATCATCACTTGTTGA
Orangutan    AGGAGCTTCCACTCTCCTCCTTCACCTCCTCCTCCTCCTCCTCATCATCATCATCCTCACTTGTTGA
Rhesus       AGGAACTTCCCC-CTCCTCCT---CCACCCCCTCGTCCTCCTCCTCCTCATCATCCTCACTTGTTGA
Marmoset     AAGAGCTTCCCCTCTCCCTGT---CCACCTCCTCCTCCTCTTCCTCATGATCATCCTCACTTGTTGA
```

S1-3: ENSP00000323252 ENST00000315302 ENSG00000177822 H-C

```
Human       ATGGGCGACCT-------GAGCCGAACCGCTCTCTGGCTGGGTGGTCGGCGGGATCCTTCAGGCTCCTTTCGGGTCCGGGACGGGGCGGCGGCAGAGGTG
Chimp       ATGGGCGACCT-------GAGCCGAACCGCTCTCTGGCTGGGTGGTCGGCGGGATCCTTCAGGCTCCTTTCGGGTCCGGGACGGGGCGGCGGCAGAGGTG
Orangutan   ATGGGCGACCT-------GAGCAGAACCGCTCTCTGGCTGGACGGTCGGCGGGATCTTTCAGGCTCCTTTCGGGTCCGGGACGGGGCGGCGGCAGAGATG
Rhesus      ATGGGCGACCT-------GAGCAGAACCGCTCTCTGGCTCGGCGGTCCGTGGGATCCTTCAGGCTCCTTTCGGGTCCGGGACGGGATGGTGGCAGAGGTG
Marmoset    ACCGGCGACCTCAGCCCTGAGCCGAACCGCTCTCTGGCTCGGCGGTCCGCGGGATCCTTCAGGCACCTTTCAGGTCCGGGAGGAGGCGGCTGCTGGGGTG

Human       GGGCGTGCGTGCTTGGGG----CTGCCGAGTGAGTGCGGCTCCCTGGTGCGCGCCCGAGCATCTCCCCGCCCTCTCCTGGGACCAGCAGTCTGCCCG---
Chimp       GGGCGTCCGTGCTTGGGG----CTGCCGAGTGAGTGCGGCTCCCTGGTGCGCGCCCGAGCATCTCCCCGCCCTCTCCTGGGACCAGCAGTCGGCCCG---
Orangutan   GGGCGTGCGTGCTTGGG-----CTGCCGAGTGAGTGCGGCTCCCT----CGTGCCCGAGCATCTCCCCGCCCTCCCCTGGGACCAGCAGTCCGCCCG---
Rhesus      GGGCATGCGCGCTTGGGA----CTGCCGAGTGCGTGCGACTCCCCCGTGGGCGCCCGAGCATCTCCCCGCCTTCCCCTGGGACCTGCAGTCCGCCGG---
Marmoset    GGGCGTGCGCGCTTGGGGCGGCCGGCCGACTGCGCGCGGCTCACC-GTGGGCGCCCGAGGGTCTCCCCGGCCTCCCCTGGGACTCGCCGCCCGCCCGCCG

Human       -TGGAGGGCTAGGAGCCCGCGACCAGGCCTGCCCCGGCCGTCGCCA------ACCTGTCCTTGGAAGCGTGGCGGAGACTGGTGCGCTCTCAGCCTCGTC
Chimp       -TGGAGGGCTAGGAGCCCGCGACCAGGCCTGCCCCGGCCGTCGCCA------ACCTGTCCTTGGAAGCGTGGCGGAGACTGGTGCGCTCTCAGCCTCGTC
Orangutan   -TGGAGGGCTGGGAGCCCGCGACCAGGCCTGCCCCGGCCGTCGCCG------ACCCGTCCTTGGAAGCGTGGCGGAGACTGGTGCGCTCTCAGCCTCCTC
Rhesus      -TGGAGGGCTGAGACCCGGCGACCAGGCCTGCCCCGGCCGCCGCCG------ACCTGTCCTTGGAAGCGTGGCGGAGACTGGTGCGCTCCCAGCCTCCTC
Marmoset    GTGGAGGGCTGGGAGCCCACGACCAGGCCTGCCCGGGCCGCCGCCGCCGCCGACCAGTCCTCGGAAGCCTGATGGAAACTGGTGCGCTTCTAGCATCCTG

Human       GCGCCAGCCGCCTCGCGCCACCCTCCACTTTCAGCTTCAGAGCAGAGGATTCCGCCGACCTCCACCTGTACCTGTACTCGGC----CGTTAGGAAATCCA
Chimp       GCGCCAGCCTCCTCGCGCCACCCTCCACTTTCAGCTTCAGAGCAGAGGATTCCGCCGACCTCTACCTGTACCTGTACTCGGC----CGTTAGGAAATCCA
Orangutan   GCGCCA--------------CCCTCCACTTTCAGCTTCAGAGCAGAGGATTCTGCCGACCTGTACCCGTACCTGTACTCGGC----CGTTAGGAAATCCA
Rhesus      GCGCCA--------------CCGTCCACTTTCAGCTTCAGAGCGGAGGATTCCGCCGACCT------GTATCTGTACTTGGC----CGTTAGGAAGTCCA
Marmoset    GCACCA--------------CCCATCACTTTCAGCTTCAGAGC---GGATTCCGCTGACCT------GTACCTA-ACTTGACTCCACGCTAGGAAATCCA

Human       CTTGCATTTTTAACTACCGTGCAGAACCAGAAACAGATCGAGCACTGGGAGAACAAGG--ACGACGCCTCCTAG
Chimp       CTTGCATTTTTAACTACCGTGCAGAACCAGAAACAGATCGAGCACTGGGAGAACAAGG--ACGACGCCTCCTAG
Orangutan   CGTGCATTTTTAACTACCGTGCAGAACCAGAAACTGATCGAGCACTGGGAGAACAAGG--ACGACGCTTCCTAG
Rhesus      CTTGCATTTTTAACTGCCGTGCAGAACCAGAAACAGATCGAGCACTGGGAGAACACGG--ACGACGCCTCCTAG
Marmoset    CTTGCCTTTGTAACTACCGTGCAGAACCAGAAACAGATCGATCACTGGGAGAACAAGGCGACGATGCTTCCTAG
```

S1-4: ENSP00000322693 ENST00000318659 ENSG00000179522 H-C

```
Human      ATGGATGTGGGCTTCCAGGGAAGGTGCTCGCGCTGGTCCCGAGCCCTCCGGGGAAGATATTCGAGCGCGGAGCGTAAGCGCAGGGCACGCCAGCCCCGGG
Chimp      ATGGATGTGGGCTTCCAGGGAAGGTGCTCGCGCTGGTCCCGAGCCCTCCGGGGAAGATATTCGAGCGCGGAGCGTAAGCGCAGGGCACGCCAGCCCCGGG
Orangutan  ATGGATGTGGGCTTCCAGGGAAGGTGCTCGCGCTGGTCCCGAGCCCTCCGGGGAAGATATTCCAGCGCGGAGCGTAAGCGCAGGGCACGCCAGCCCCGGG
Rhesus     ATGGATGTGGGCTTCCAGGGAAGGTGCTCGCGCTGGTTCCGAGCCCTCCGGGGAAGATATTCGAGCGCGGAGCGTAAGCGCAGGGCACGCCAGCCC-GGG
Marmoset   ACGGATGTGGGCTCCCAGGGAAGGTGCTCGCGCCCGTCCCGCGCCCTCCGGGGAAGATATCCGAGCGCGGAGCGTCAGCGCAGGACACGCCAGCCCCGGG

Human      AGCCGCGGGAGCAGGCGCCGCGCGTCTCTGCACCACCGGGCCGCCTCCCAGCCTTCTTTCCCCAGTTTGCCCTCCTGCCGCAGTCCGGGCCGAGATTAAT
Chimp      AGCCGCGGGAGCAGGCGCCGCGCGTCTCTGCACCATCGGGCCGCCTCCCAGCCTTCTTTCCCCAGTTTGCCCTCCTGCCGCAGTCCGGGCCGAGATTAAT
Orangutan  AGCCGCGGGAGCAGGCGCCGCGCGTCTCTGCACCACCGGGCCGCCTCCCAGCTCTCTTTCCCCAGTTTGCCCTCCTGCCGCAGTCCGGGCCGAGATTAAT
Rhesus     GGCCGCGGAAGCAGGCGCTGCGCGTCTCTGCACCACCGGGCCGCCTCCCAGCCCTCTTTCCCCAGTTTGCCCTGCTGCCGCAGTCCGGGCCCAGATTAAT
Marmoset   GGCCGCGGGAGCAGGCGCTGCGCGTCTCTGCACCACCGTGCCGCCTCCCAGCCCTCTTTCCCCAGTTCGCCCTCCTGCCGCAGTCCGGGCCCAGATTA-T

Human      TCTCTGCACTTGTGAGTGGGCACACACA--AGTTCTCCGGGCACGATCCTTTCATCTATTTCCCTGG-GGGAGTCCACCTTTTTAACGATTAACCTCCTA
Chimp      TCTCTGCACTTGTGAGTGGGCACACACA--AGTTCTCTGGGCACGATCCTTTCATCTGTTTCCCTGG-GGGAGTCCACCTTTTTAACGATTAACCTCCCA
Orangutan  TCTCTGCACTTGTGAGTGGGCACACACA--AGTTCTCCGGACACGATCCTTTAATCTGTTTTCCTGG-GGCAGTCCACCTTTTTAACGATTAACCTCCCA
Rhesus     TATCTGCACTTGTGAGTGGGCACACACACAAGTTCTCCGGACACGATCCTTTCATCTGTTTCCCCGG-CGGAGTCCACCTTTTTAAAGATTAACCTCCCA
Marmoset   TCTCTGCACTTGTCAGTGGGCACACACA--AGTTCTCCGGGCACGAACCTTCCATCCGTTTCCCCGGAGGGAGTCCACCTTTTTAACGATTAACCTCCCA

Human      GCTACCGCGGGCAAGGTGGCAGGATGCGAGTGGGGCGGGGAGGGGCGTTTCACACGTTCAGAGGCACCAAAATTAGCTGCCAGTGCTAAAAGGCTTTGCT
Chimp      GCTACCGCGGGCAAGGTGGCAGGATGCGAGTGGGGCGGGGAGGGGCGTTTCACACGTTCAGAGGCACCAAAATTAGCTGCCAGTGCTAAAAGGCTTTGCT
Orangutan  GCTACCGCGGGCAAGGTGGCAGGATGCGAGTGGGGCGGGGAGGGGCGTTTCACACGTTCAGAGGCACCAAAATTAGCTGCCAGTGCTAAAAGGCTTTGCT
Rhesus     GCTACCGCGGGCAAGGGGGCAGGATGCGAGTGAGGCGGGGAGGGGCGTTTCACACGTTCAGAGGCACCAAAATTAGCTGCCAGTGCTAAAAGGCTTTGTT
Marmoset   GCTACGGCGGGCAAGGTGGCAGGATGCGAGTGGGGCGGGGAGGGGCGCTTCGCACATTCAGAGGCACCAGAATTAGCTGCCAGTGCTCAAATGGCTTGCT

Human      TTCTTCGGTTTTTGACAAATAAATGGGGTGGGATGCTTGCTTGGCCGCCCGCTGCCCCAGCCCGAGCCCTGGGCTCACTTAGCAGCCTGATGCCGAGTTT
Chimp      TTCTTCGGTTTTTGACAAATAAATGGGGTGGGATGCTTGCTTGGCCGCCCGCTGCCCCAGCCCGAGCCCTGGGCTCACTTAGCAGCCTGATGCCGAGTTT
Orangutan  TTCTTCGGTTTTTGACAAACAAATGGGGTGGGATGCTTGCTTGGCTGCCCGCTGCCCCAGCCCGAGCCCTGGGCTCACTTAGCAGCCTGATGCCGAGTTT
Rhesus     TTCTTCGGTTTTTGACAAACAAATGAGGTGGGATGCTTGCTTGGCCGCCCGCTGCCCCAGCCCGAGCCCTTGGCTCACTTAGCAGCCTGATGCCGACTTT
Marmoset   TTCTTTGGTTTTTGACAAACAAACGGGGTGGGATGCTTTCTTGGCCGCCGGCCGCCG-GGCCTGAGCCCCGGGCTCACTTCGCAGCCTGATGCCGAGTTT

Human      CAGAC-GCAGTCCTGCTGCGCTTACACCCGGGCTTCTTCGCCCCCTTGCCAAAGTCTGCAGCCCGATGGATGCTGGGCGCGGGCTTTCCCTGAGCGCTTT
Chimp      CAGAC-GCAGTCCTGCTGTGCTTACACCCGGGCTTCTTCGCCCCCTTGCCAAAGTCTGCAGCCCGATGGATGCTGGGCGCGGGCTTTCCCTGAGCGCTTT
Orangutan  CAGAC-GCAGTCCTGCTGTGCTTACACCCGGGCTTCTTCGCTCCCTTGCCAAAGTCTGCAGCCCGGTGGATGCTGGGCGCGGGCTTTCCCTGAGCGCTTT
Rhesus     CAGAC-GCAGTCCTGTTCTGCTTACACCAGGGCTTCTTCGCCCCCTTGCCGAAGTCTGCAGCCCGGTGGATGCTGGGCGCGGGCTTTCCCTGGGCGCTTT
Marmoset   GAGACCGCAGTCCCGCTCTGCTGACACCCGGGCTTCT-CGCCCCATTGCCGAAGTCTGCGGCCCGAGGGATGCTGGGCGCGGGCTTTCCCTGCGCGCTTT

Human      AACGCAGCTTAGGCTAAAGCCCCAGAGCTCCCACCTTCT-ACCTCCTGTTTATCCGCCCGCCCCTCTACCACCGCCAAAGGACGTGCCCCTTCAGTAG
Chimp      AACGCAGCTTAGGCTAAAGCCCCAGAGCTCCCACCTTCT-ACCTCCTGTTTATCCGCCCGCCCCTCTACCACCGCCAAAGGACGTGCCCCTTCAGTAG
Orangutan  AACGCAGCTTAGGCTAAAGCCCCAGAGCTCCCACCTTCTCACCTCCTGTTTATCGGCCCGCCCCTCTACCACCGCCAAAGGACGTGCCCCTTCAGTAG
Rhesus     AACGCGGCTTAGGCTAAAGCCCCAGAGCTCCCACCTTCTCACCTCCTGTTTATCGGCCCGCCCCTCTACCACAGCCAAAGGACGTGCCCCTTCAGTAG
Marmoset   AACGCTGCTTGGCTAAAGCCCCAGAGCTCCCACCTTCGCAGCTCCTGTTCCTCGGCCCGCTCCTCTACCTCCGCCAAAGGACGTGCCCCTTCAGTAG
```

S1-5: ENSP00000322161 ENST00000324987 ENSG00000215071 H-C

```
Human      ATGCAGATATGCTTCTTAGCCTTGTGGGGTGGAACT----ATGGCTGTAGCTACTTCTGCTGAAACTGCCCTTAGGACATCATATGCATCTTTGAAAGCC
Chimp      ATGCAGATATGCTGCTTAGCCTTGTGGGGTGGAACT----ATGGCTGTAGCTACTTCTGCTGAAACTGCCCTTAGGACATCATATGCATCTTTGAAAGCC
Orangutan  ATGCAGATATGCTTCTTAGCCTTGCGGGGTGGAACT----ATGGCTGTAGCTACTTCTGCTGAAACTGCCCTTAGGA---CATATGAATCTTTGAAAGAC
Rhesus     ATGCAGATACGCTCCTTAGCCTCGTGGGGTGGAACTTACCATGGCTGTAGCTACTGCTGCTGAAACTGCCCTTAGGA---CATATGAATCTTTGAAAGAC
Marmoset   ACCCAGATACACTC--TAAGCTTGTGGGGT-GAACTTACCACAGCTGTAGCTACT-CTGCAGAAACTG-CCTTAGGG---CATATGAATC-TTGAAAGAC

Human      AAATACATAAGTGAACACATCTGCGTGTATGTGTATACAC-CATACATGCACACATTCATACGCATGCATATACGTGTGCATGTTTTAGTTAACAGAAGT
Chimp      AAATACATAAGTGAACACATCTGCGTGTATGTGTATACAC-CATACATGCACACATTCATACGCATGCGTATATGTGTGCATGTTTTAGTTAACAGAAGT
Orangutan  AAATACATAAGTGAATACATCTGCGTGTATGTGTATACACGCACACATGCACACATTCATATGCATACGTATATGTGTGCGTGTTTTAGTTAACAGAAGT
Rhesus     AAACACATAAGTGAATACATCTGCATGTATGTGTATACACGCATACACGCACACATTCATGCACATATGTATACGTGTGCACGTTTTAGTTAATGGAAGT
Marmoset   AAA-ACATAAGTGAATCCATCTGCGTGTATGTGTCCATATGCGTACATGCACACATTCATACGCATATGTACACGTGTGCACATTTTAGTTAACAGAAAT

Human      CCTTGGGGGCTGGACAATATTTTGGCTCCAAGGGATGTGAGCAACTATAAAGTCATGAGACTGAGTTTCTTGTGCGGCTCCAGCGGCTCTGAAAGCCCCT
Chimp      CCTTGGGGGCTGGACAATATTTTGGCTCCAAGGGATGTGAGCAACTATAAAGTCATGAGACTGAGTTTCTTGTGCGGCTCCAGCGGCTCTGAAAGCCCCT
Orangutan  CCTTGGGAGCTGGACAACGTTTTGGCTCCAAGGGACATGAGCAACTATAAAGTCATGAGACTGAGTTTCTTGTGCAGCTCCAGCAGCTCTGAAAGCCCCT
Rhesus     CCTTGGGAGCTGGACAATGTTTTGGCTCCAAGGGATGTGAGCAATCATAAAGTCATGAGACTGGGTTTCTTGTGCGGCCCCAGCAGCTCTGAGAGCCCCT
Marmoset   CCTTGGGAGCTGGACAA--GTTTGCTTTCAAGGGATGTGACTAACTATAAAGTCGTGAGACTGAGTTTCTTGTGCGGCTCCTGCCACGCTGAAAGCCCCT

Human      CTGAAGGGAGTCCACCAGCGCACAGTGTTACATTCACCAGGCCAAGGGGTCTGCAGAGGTGCTGGTGTTGA
Chimp      CTGAAGGGAGTCCACCAGCGCACAGTGTTACATTCACCAGGCCAAGGGGTCTGCAGAGGTGCTGGTGTTGA
Orangutan  CTGAAGGGGGTCCACCAGCGCACAGTGTTACATTCACCAGGCCAAGGGGTCTGCGGAGGTGCTGGTGTTGA
Rhesus     CTGAAGGGAGTCCACCAGCG--CGGTGTTAC-------------AAGGGTCTGCAGAGGTGCTGGTGTCGA
Marmoset   CTAAAGAGAGTCTGCCAGCC--CTGTGTTATATTCACCAGGCCAATGGGCCTGTGGAGGTGCTGGGGTCCA
```

S1-6: ENSP00000325255 ENST00000326341 ENSG00000178803 H

```
Human      ATGGAGCAGGACTGGCAACCTGGAGAGGAAGTCACTCCTGGTCCTGAGCCCTGTTCAAAGGGCCAGGCTCCCCTCT-ACCCCATTGTCCATGTGACAGAG
Chimp      ATGGAGCAGGACTGGCAACCTGGAGAGGAAGTCACTCCTGGTCCTGAGCCCTGTTCAAAGGGCCAGGCTCCCCTCTTACCCCATTGTCCATGTGACAGAG
Orangutan  ATGAAGCAGGACTGGCAACCTGGAGAGGAAGTCACTCCTGGTCCCGAGCCCTGTTCAAAGGGCCAGGCTCCCCTCT-ACCCCATTGTCCATGTGACAGAG
Rhesus     ACGGAGCAGGATTGGCAACCTGGAGAGAAAGTCAGTCCTGGTCCTGAGCCCTGTTCAAAGGGTCAGGCTCCCCTCT-ACCCCACTGTCCATGTGACGGAG
Marmoset   ATGGAGCAGGCCTGGCAACTTGGAAAGAAAGTCACTCCTGGTCCCAAGCCCTGTTCAAAGGGCCAGGCTCCCCTCT-ACCCCACTGTCCATGTGACGGAC

Human      CTCAAACACACAGACCCCAACTTTCCCTCCAACTCCAATGCTGTCGGCACCTCAAGTGGCTGGAACAGGATTGGCACGGGCTGCAGCCATACCTGGGACT
Chimp      CTCAAACACACAGACCCCAACTTTCCCTCCAACTCCAATGCTGTCGGCACCTCAAGTGGCTGGAACAGGATTGGCACGGGCTGCAGCCATACCTGGGACT
Orangutan  CTCAAACACACAGACCCCAACTTTCCCTCCAACTCCAATGCTGTCGGCACCTCAAGTGGCTGGAACAGGATTGGCATGGGCTGCAGCCATACCTGGGACT
Rhesus     CTCAAACA----GACCCCAACTTTCCCTCCAACTCCAATGCTGTCAGCACCTCAAGTGGCTGGAACAGGATTGGCACAGGCTGCAGCCATACCTGGGACT
Marmoset   CTCAAACAGGTGGACCCCAACTGTTCCTATAACTCGAATACCATCAGCACCTCAGGGGGCTGGAACAGGATCAGCAGAGGCTGTAGCCAGACCTGGGCCT

Human      GGAGGTTCTCCTGCACCCAGCAGGCCCTTTTGCCCCTACTAGGAGCCTGGGAATGGAGCAT-TGACACAGAAGCAGGAGGAGGAAGGAGAGAGCAGAG-C
Chimp      GGAGGTTCTCCTGCACCCAGCAGGCCCTTTTGCCCCTACTAGGAGCCTGGGAATGGAGCAT-TGACACAGAAGCAGGAGGAGGAAGGAGAGAGCAGAGGC
Orangutan  GGAGGTTCTCCTGCACCCAACAGGCCCTTTTGCCCCTACTAGGAGCCTCGGAATGGAGCAT-TGACACAGAAGCAGGAGGAGGAAGGAGAGAGCAGAGGC
Rhesus     GGAGGTTCTCCTGCACCCAACAGGCCCTTTTGCGTCTACTAGGAGGCTTGGAATGGAGCAT-TGACACAGAAGCAGGAGGAGGAAGGAGAGAGCAGAGAC
Marmoset   GGAGGTTCTCCTGCACCCAACGGGCCCTTATGCCCCCACTAGGAGGCTTGGAATGGAGCCTCTGACACAGAAGTGGGAGGAGGGAGGAGAGTGCAGAG-C

Human      CAGAAACCCTGCAGCAACGGAGGGCCTGCAGCAGCTGGAGAGGGCCGAGTCCTCCCAAGCCCCTGCTTT-----CCA-TGGAGCACTTGCCAGGCAGCCA
Chimp      CAGAAACCCTGCAGAAACGGAGGGCCTGCAGCAGCTGGAGAGGGCTGAGTCCTCCCAAGCCCCTGCTTT-----CCA-TGGGGCACTTGCCAGGCAGCCA
Orangutan  CAGAAACCCTGCAGAAACGGAGGGCCTGCAGCAGCTGGAGAGGGCTGAGTCCTTCCAAGCCCCTGCTTT-----CCA-TGGGGCACCTGCCAGGCAGCCA
Rhesus     CAGAGACCCTGCAAAAATGGAGGGCCTGCAGCAGCTGGAGAGGGCTGAGTCCTCCCAAGCCCCTGCTTT-----CCA-TGGGGCACTTGCCAGGCAGCCA
Marmoset   CAGAGACCCTGCAGAAGCGGAGG-TTTGCAGCAGCTGGAGAGGGCTCAGTCCTCCTGAGCCCCTGCTTTIIIIICCACT---GCACTTGCCAGGCAGCCA

Human      TTCACAAAGTGTGTCGTTGGCAGGGATGCACCAGACCAGCTCTCCTGGCACCATCCCTGGCCACACTCAAGGAACACAGTTATCCCTGA
Chimp      TTCACAAAGTGTGTTGTTGGCAGGGATGCACCAGACCAGCTCTCCTGGCACCATCCCTGGCCACACTCAAGGAACACAGTTATCCCTGA
Orangutan  CTCACAAAGCATGTCGCTGGCAGGGATGCGCCAGACCAGCTCTCCTGGCACCATCCCTGGCCACACTCAAGGAACACAGTTATCCCTGA
Rhesus     CTCACAAAGCATGTCGCTGGCAGGGATGCGCCAGACCAGGTCTCCTGGCACCATCTCTGGCCACACTCAAGGAACACAGTTATCCCTGA
Marmoset   CTCACAAAGCGTGTTGCTGGCAGGGATGTGCCAGACTAGGTCTCCTGGCACCGTCCTTGGCCACACTCAAGGAACACAGTTATCCCTGA
```

S1-7: ENSP00000330965 ENST00000327903 ENSG00000182457 H-C

```
Human      ATGGACTCCAGATTTCCAGATTTTCAAGACCTGGACCTGGAACCCGAAAGAGCTTGTCACGATGCGGCAGGAACACTGGAGGTAGA-TTTTTTTTT-ATT
Chimp      ATGGACTCCAGATTTCCAGATTTTCAAGAACTGGACCTGGAACCCGAAAGAGCTTGTCAGGATGCGGCAGGAACACTGGAGGTAGA-TTTTTTTTT-ATT
Orangutan  ATGGACTCCAGATTTCCAGATTTTCAAGAACTGGACCTGGAACCCGAAAGAGCTTGTCAGGATGCGGCAAGAACACTGGAGGTAGATTTTTTTTTT-ATT
Rhesus     GTGGACTCCAGATTTCCAGATTTTCAAGAACTGGACTTGGAACCCGAAAGAGCTTATCGGGATGTGGCAGGAACACTGGAGGTAGATATTTTTTTT-ATT
Marmoset   ATGGACTCCAGATTTCCAGATTTTCAAGAACTGGACCTGGAACCTGAAAGAGCTTGTCAGGATGTGGCAGGAACACTGAAGGTAGATTTTTTTTTTAATT

Human      TTTGAATTTTGGGACTGTTGACCTTGCTGTGAGAAAAGAGACAACGACTGAGCAAGCACTACCACCAGCACTGTTACTGGGAATT--AGAAGACCTGAGT
Chimp      TTTGAATTTGGGGACTGTTGACCTTGCTGTGAGAAAAGAGACAACGACTGAGCAAGCACTACCACCAGCACTGTTACTGGGAATT--AGAAGACCTGAGT
Orangutan  TTTGAATTTTGGGACTGTTGACCTTGCTGTGAGAAAAGAGACAACGACTGAGCAAGCACTACCACCAGCATTGTTACTGGGAATT--AGAAGACCTGAGT
Rhesus     TTTGAATTTTGGGACTGTTGACCTTGCTGTGAGAAAAGAGACAACGACTGAGCAAGCACTACCACCAGCACTGTTACTGGGAATT--AGAATACCTGAGT
Marmoset   TTTGAATTTTGGGACTGTTGACCTTGCTGTAAGAAAAGAGACAACGACTGAGCAAGCACTACCACCAGCTCTGTTACTGGGAATTAGAGAAGACCTGAGT

Human      TTCTGTCCAGACCCTCAGTGCAAACTGAGGATGCTCCATCCAAAGTGAATTATG-----TCCTGTGCCTCCTGATTGCTGAGTGTTCACCTGGACCTTCT
Chimp      TTCTGTCCAGACCCTCAGTGCAAACTGAGGATGCTCCATCCAAAGTGAATTATG-----TCCTGTGCCTCCTGATTGCTGAGTGTTCACCTGGACCTTCT
Orangutan  TTCTGTCCAGACCCTCAGTGCAAACTGAGGACGCTCCATCCAAAGTGAATCATG-----TCCTGTGCCTCCTGATTGCTGAGTGTTCACCTGGACCTTCT
Rhesus     TTCTGTCCAGACCCTCAGTGGAAACTGAGGACACTCCATCCAAAGTGAATTATG-----TCCTGTGCCTCCTGATTGCTGAGTGTTCACCTGGACCTTCT
Marmoset   TTCTGTCCAGACCCTCAGTGCAAACTGAGGATGCTCCATCCAAAGTGAATTATG-----TCTTGTGCCTCCTGATTGCTGAGTGTTCACCTGGACCTTCT

Human      GACTACCTTCCCTGTGCTATTCCATCAG----------CCTACAGACCTGGTACCTGGATTTTTGCCCGAGATGATTCCTACCACCTTACTACTGACGAAG
Chimp      GACTACCTTCCCTGTGCTATTCCATCAG----------CCTACAGACCTGGTACCTGGATTTTTGCCCGAGATGATTCCTACCACCTTACTACTGAAGAAG
Orangutan  GACTACCTTCCCTGTGCTATTCCATCAG----------CCTACAGACCTGGTACCTGGATTTTTGCCCGAGATGATTCCTACCACCTTACTACTGAAGAAG
Rhesus     GACTACCTTCCCTGTGCTATTCCATCAG----------CCTACAGACCTGGTACCTGGATTTTTGCCCAAGATGATTCCTACCACCTTACTACTGAAGAAG
Marmoset   GACTACCTTCCTTGTGCTATTCCATCAGCCTACAGACCCTACAGACCTGGTACCTGGATTTTTGCTGAAGATGATTCCTACCACCTTACTACTGAAGAAG

Human      ACACCCATTCCAGTGGACCACTGTGA
Chimp      ACACCCATTCCAGTGGACCACTGTGA
Orangutan  ACACCCATTCCAGTGGACCACTGTGA
Rhesus     ACACCCATTCCAGTGGACCACTGTGA
Marmoset   ACACCCATTCCATTGGACCACTGTGA
```

S1-8: ENSP00000359554 ENST00000370523 ENSG00000174407 H-C-O

```
Human       ATGGATACGGGGTCCCGGGAGGCAGCCTTCACCAGGGACCTGCCCAGGAAACCAGCAGATGCCCAG-----GTCAGCCCTGAAATGTCCATTACTCACAA
Chimp       ATGGACACGGGGTCCCGGGAGGCAGCCTTCACCAGGGACCTGCCCAGGAAACCAGCAGATGCCCAG-----GTCAGCCCTGAAATGTCCATTACTCACAA
Orangutan   ATGGACACGGGGTCCCGGGAGGCAGCCTTTACCAGGGACCTGCCCAGGAAACCATCAGCTGCCCAG-----GTCAGCCCTGAAATGTCCATGACTCACAA
Rhesus      AGGGACACAGCATCCCGGGAGGCAGCCTTCACCAGGGACCTGCCCAGGAAACCGCCAGCTGCCCAG-----GTCAGCCCTGAAATGTCCATTACTCACAA
Marmoset    GGGGACACCAGGTCCCAGGAGGCAGCCTTTACCAGGGATCTGCCCAGGAAGCTGCCAGCTGCCCAG-----GTCAGCCCTGAAATGTCCATTTAACAAAA

Human       AGAAAAGGAAAATGCTCATTTGAAAGA-AATTCTTCTCTTTGTCAATGCTGAGGCATTCTCTCAGCCCCAGCCCCACAGTGCTCCAGTGTGTGAAGGGCA
Chimp       AGAGAAGGAAAATGCTCATTTGAAAGA-AATTCTTCTCTTTGTCAATGCTGAGGCATTCTCTCAGCCCCAGCCCCACAGTGCTCCAGTGTGTGAAGGGCA
Orangutan   AGAAAAGGAAAATGCTCATTTGAAAGA-AATTCTTCTCTTTGTCAATGCTGAGGCATTCTCTCAGCCCCAGCCCCACAGTGCTCCAGTGTGTGAAGGGCA
Rhesus      AGAAAAGGAAAATGCTCATTTGAAAGACAATTCTTCTCTTTGACAATGCTGAGACATTCTCCCAGCCCCAGCCCCACAGTGCCCCAGTGTGTGAAGGACA
Marmoset    AGAAAAGGAAAATGCTTATTTGAAAG--AATTCTTCTCTTTGGCAATGCTGAGACATTCTCCTAGTCTCAGCCCCACAGAGCTCCAGTGTGTGACAGACA

Human       ACAGCTGACGGGGAAGTTCAGCACATCTGTGCTGACCAGGGCTGGAGGCGATGCCAGTCCGTGCAGCTGGGAACGCCTCCTCTGCTATGGCTGGAGCCAC
Chimp       ACAGCTGACGGGGAAGTTCAGCACATCTGTGCTGACCAGGGCTGGAGGTGATGCCAGTCTGTGCAGCTGGGAACGCCTCCTCTGCTATGGCCGGAGCCAC
Orangutan   ACAGCTGACAGGGAAGTTCAGCACGTCTCTCCTGACCAGGGCTGGAGGCGATGCCAGTCCGTGCAGCTGGGAACGCCTCCTCCACTGTGGCTGGAGCCAC
Rhesus      ACAGCTGTTGGGGAAGTTCAGTGCATCTGTCCTAACCAGGGCTGGAGGTGGTGCCAGTCTGTGCAGCTGGGAACACCTCCTCTGCTGTG-----------
Marmoset    ACAGCTGATGGGGAAGTTCCGCGTGTCCGTCCTAACCAGGGCTAGAGGCGATGACTGTCTGTGCGGCTGGGAAAGCCTCCTCC-TTGTGGCTGCAGCCAC

Human       TGCTGA
Chimp       TGCTGA
Orangutan   TGCTGA
Rhesus      -GCTGA
Marmoset    TGCTGA
```

S1-9: ENSP00000359566 ENST00000370535 ENSG00000203930 H

```
Human      ATGCCACTG-----GAAAAATTCGTAGACATGGAATTCCTGGATCAAATG-----ATACTAAGGGGGACTTGCCTGGAAGCAGACTTGTGTCCGCAGTAT
Chimp      ATGCCACTG-----GAAAAATTCGTAGACATGGAATTCCTGGATCAAATG-----ATACTAAGGGGGACTTGCCTGGAAGCAGACTTGTGTCCGCAGTAT
Orangutan  ATGCCACTG-----GAAAAATTCGTAGACATGGAATTCCTGGATCAAATG-----ATACTAAGGGAGACTTGCCTGGAAGCAGACTTGTGTCCGCAGTAT
Rhesus     GTGCCGCTG-----GAAAAATTCATAGACATGGAATTCGTGGATCAAATG-----ATACTAAGGGAGACTTGCCTGGAAGCAGACTTGTATCCGCAGTGT
Marmoset   ATGCCACTG-----GATAAATTCGTAGATGTGGAATTCCTGGATCAAATG-----ATACTAAGGGAGACTTCCCTGGAAGCAGACTTGTATCCGCAGTGT

Human      ACAAACTCACCCTTTAATGCACACACTGACCAGATAACACAA--ACACATTTACCCCCACAGCACCTAGACTCTTCTCACAGTACACACACTTGCCTTCC
Chimp      ACAAACTCACCCTTTAAAGCACACACTGACCAGATAACACAAACACACATTTACCCCCACAGCACCTAGACTCTTCTCACAGTACACACACTTGCCTTCC
Orangutan  ACAAACTCACCCTTTAATGCACACACTGACCAGATAACACAAACACACATTTACCCCCACAGTACATAGATTCTTCTCACAGTACACACACTTGCCTTCC
Rhesus     ACAAACTCATCCTTTAGTGCGCACACTGGCCGGATAACACAAACACACATTTACTCCCACAGCACACAGACACTTTCCACAGTACACACACTTGCCTTCC
Marmoset   ACAAACTTACCCTGTAATGCACACAATGGCCAGATAACACAAACACACATTTATCTCCACAGCACACAGACTCTTGTCACAGTACACAGACTTGCTTTCC

Human      AGTACACAGACATATCCAAAGGCACACCGACAAGCACCACAGCAAAAAGATGTTCCCCAGAACACATGGACAC-CCCAATCCCCGTATCATTAGACTCTC
Chimp      AGTACACAGACATATCCAAAGGCACATCGACAAGCACCACAGCAAAAAGATGTTCCCCAGAACACATGGACAC-CCCAATCCCCGTATCATTAGACTCTC
Orangutan  AGTACACAGACATATCCAAAGGCACACCGACAAGCACCACAGCAAAAAGATGTTCCCCAGAACACATGGATAC-CCCACTCCCCGTATCATTAGACTCTC
Rhesus     AGTACACAGACATATCCAAAGGCACACCGACAAACACCACAGCAGAAAGATTTTCCCCAGAACACACGGACAC-CCCACTCCCCGTATCATTAGACTCTC
Marmoset   AGTACACAGACATATCCAAAGGCACACCGACAAGCACCACAGCAAAAAGACTTTCCCCAGAGCACACAGACACTCCCATTGCATGTATCAATAGACTCTC

Human      CCAGAGGCACGTATGTACTGCTTAA
Chimp      CCAGAGGCACATATGTACTGCTTAA
Orangutan  CCAGAGGCACATATGTACTGCTTAA
Rhesus     CCAGAGGCACATATGTACTCCTTCA
Marmoset   CCAGAGGCACCTATATTCTCCTTAA
```

S1-10: ENSP00000362265 ENST00000373170 ENSG00000204091 H-C-O

```
Human      ATGAAGAGGAGGGAGGCAGTCTGCGCGCACCGCCA-TTTTCTAGGAACTGGGAA---------GCCCCCCCACCCCTTAGGAAGATCCATCCCTGTGGAA
Chimp      ATGAAGAGGAGGGAGGCAATCTGCGCGCACCGCCA-TTTTCTAGGAACTGGGAA---------GCCCCCCCACCCCTTAGGAAGATCCATCCCTGTGGAA
Orangutan  ATGAAGAGGAGGGAGGCAGTCTGCACGCACCGCCA-TTTTCTAGGAACTGGGAA---------GCCCCCCCACCCCTTAGGAAGATCCATCCCTGTGGAA
Rhesus     ATGAAGAGGAGGGAGGCAGTCTGCACGCACCGCCATTTTTCTAGGAACTGGAAAGCGCCTCCCGCTCCCCCCGCCCTTAGGAAGATCCATCCTTGTGGAA
Marmoset   AGGAAGAGGAGGAAGGCAGTCTGCATATACCACCA-TTTTCTAGAGACTGGGAA---------G-----ACAGCCCTTAGGATGATCCATCCCTGTGTAA

Human      CCTTGCCCAGGCTTACCAGCCTTTGCTGAGGTTGATCTATT-------------------------------------GT--------------CCCTCC
Chimp      CCTTGCCCAGGCTTACCAGCCTTTGCTGAGGTTGATCTATT-------------------------------------GT--------------CCCTCC
Orangutan  CCTTGCCAAGGCTTACCAGCCTTTGCTGAGCTTGAACTATT-------------------------------------GT--------------CCCTCC
Rhesus     CCTTACCCAGGCTTACCAGCCTTTGCTGAGCTTGATCTATTTGGACTAGTATCAGTCAGTA--GGACTTGAGACGTTGGTTCTCTACACTTGCACCCTCC
Marmoset   CCCTGCCCAGGCTTACCAGCCTTTCCTGAGCTGGAGCTATTTGGACTAGTATCTAACAGGACTTGACATTGG------GT-CTCCACACTTGCACCCTCC

Human      TTGTCCCCATCAAAATATCCAGCACTCCACCTTCAGGGAGTAGACTTGACCCTCAAATAGCAAG------TTCAGCCTTCCCAGGTCTAGGTTCCCTGGG
Chimp      TTGTCCCCATCAAAATATCCAGCACTCCACCTTCAGGGAGTAGACTTGACCCTCAAATAGCAAG------TTCAGCCTTCCCAGGTCTAGGTTCCCTGGG
Orangutan  TTGTCCCCATCAAAATATCCAGCACTCCACCTTCAGGGAGTAGACTTGACCCTCAAATAGCAAG------TTCAGCCTTCCCAGGTCTAGGTTCCCTGGG
Rhesus     TTGTCCCCATCAAAATATCCAGTACTCTACCTCCAGGGAGTAGACTTGACCCTCAAATAGCAAG------TTCAGCCTTCCCAGGTCTAGGTTCCCTGGG
Marmoset   TTGTCCCCATCAAAATATGCAGTACTCTGCCTTCAGATAGGAGACATGACCCTTGCACAACAAGAAAAAGTCTAACCTTCCTGGGTCAAGGTTC------

Human      AGGTCAAGATTCGTCTGGTTCCTTAGTACAGAGGGCTAGCTGTGAGTTGGAATCCCCCTATGAGCTTTAG
Chimp      AAGTCAAGATTCGTCTGGTTCCTTAGTACAGAGGGCTAGCTGTGAGTTGGAATCCCCCTATGAGCTTTAG
Orangutan  AGGTCAAGATTCGTCTGGTTCCTTAGTACAGAGGGCTAGCTGTGAGTTGGAATCCCCCTATGAGCTTTAG
Rhesus     GGTTTAAGATTCGTATGGTTCCTTAGTATAGAGGGCTAGCTGTGAGATGGAATCCCACTATGAGCTTTAG
Marmoset   ------------CTATGCTTCCTTAGTA--GAGGGCTAGCTGTGAGTTGGAATCCCACTATGAGCTTTAG
```

S1-11: ENSP00000366008   ENST00000376812   ENSG00000204626   H

```
Human       ATGCACAGCCTGCCACGGAGTGGCTCTATCAGGCGCACACACAGCGACACACAGGCCACTGGCTGGCCTCCTCCCCAGCGCATTGGGG-ACAGCCCAGGC
Chimp       ATGCACAGCCTGCCACGGAGTGGCTCTATCAGGCGCACACACAGCGACACACAGGCCACTGGCTGGCCTCCTCCCCAGCGCATTGGGG-ACAGCCCAGGC
Orangutan   ATGCACAGCCTGCCATGGAGTGGCTCTATCAGGCGCGCACACAGCGACACACAGGCCACTGGCTGGCCTCCTCCCCAGCGTATTGGGG-ACAGCCCAGGC
Rhesus      ATCCACAGCCTGCCA-------------TCAGGCGCACACAGCGACACACACAAGCCACTGGTTGGCCTCCTCCCCAGCGCCCTGGGG-ACAGCCCAGGC
Marmoset    ATCCACGGCCCACCA-GGAGTGGCTCTATCAGGTGCACACACAGCAACACACAGGCCACTGGCTGGTCTCCTCCCCAGCACACCGGGACACAGCCCAGGC

Human       CCTTCTCCAGCATTTCTGTCCTGCCCACCTTCCCTCTGTGGAGGAGCAGCCCAGACAGGAGACCCTGTGGCCCTGCCCCATGGCCCAGAGAAATGGGTGT
Chimp       CCTTCTCCAGCATTTCTGTCCTGCCCACCTTCCCTCTGTGGAGGAGCAGCCCAGACAG--GACCCTGTGGCCCT-CGCCATGGCCCAGAGAAATGGGTGT
Orangutan   CCTTCTCCAGCACTTCTGTCCCGCCCACCTTCCCTCTGTGGAGGAGCAGCCCCGACAGGAGACCCCGTGGCCCTCCTCCATGACCCAGAGAAGTGGGTGC
Rhesus      CCTTCTCCAGCGCTTCTGTCCTGCCCACCTTCCCTCTGTGGAGGAACGGCTCAGACAGGACACCCCGTGGCCCTCCCCCAGGGCCCAGAGAAGTGGGTGC
Marmoset    CCTCCTCCAGCGCTTCTGTCCTGCCCACCGCCCCTCTGTGGAGGAGCAGCCCAGGCAGGAGACCCCATAGCCCTCCTCCATGGCCCAGAGAAGTGGGTGT

Human       GGGGCGGTGGCCTCTCCCCCAGGAATCCTCATTCCTGGGGCATCAAGGCCCACGGACTTAGACCACCCTGGGCCCCCAGGCTAGAAAGATGCATGGTCCC
Chimp       GGGGCGGTGGCCTCTCCCCCAGGAA----------TGAGGCATCAAGGCCCACGGACTTAGACCACCCTGGGCCCCCAGGCTAGAAAGATGCATGGTCCC
Orangutan   AGGGTGCTGGCCTCTCCCCCAGGAA----------TGAGGCAGCAAGGCCCACGGACTTAGACCACCCTGGGCCCCCAGGCTAGAAAGATGCATGGCCCC
Rhesus      GGGGCGGTGGCCTCTCCCCCAGGAA----------TGAGGCATCAAGGTCGACGGACTTAGACCACCCTGGGCCCCCAGGCTAGGAAGATGCATGGCCCC
Marmoset    GGGGTGGTGGCCTCTCCCCCAGGAA----------TGAGGCATCAGAGCACACGCACTTAGACCACCCTGGGCCCTGAGGCT------------GCTACC

Human       AGAGTCAGAATGGGCACCATGGCAACCCCAGCTACCCTGTGAGCCGAAGTGGCTGGGGAGCAGGAAGTCGAAGCCTCACAGAGAAAGTGGTCTCCGGGGA
Chimp       AGAGTCAGAATGGGCACCATGGCAACCCCAGCTACCCTGTGAGCCGAAGTGGCTGGGGAGCAGGAAGTCGAAGCCTCACAGAGAAAGTGGTCTCCGGGGA
Orangutan   AGAGTCAGAATGGGCACCGTGGCAACCCCAGCTACCCTGTGAGCCAAAGTGGCTGGGGAGCAGGAAGTCGAAGCCTCACAGAGAAAGTGGTCTCCGGGGA
Rhesus      AGAGTCAGAATGGGCACCATGGCAACCCCGGCTACCCTGGGAGCCGAAGTGGCTGGGGAGCAGGAAGTAGAAGCGTCACAGAGAAAGCGGCCCCCGGGGA
Marmoset    AGAGTCAGAATGGGCACAATGGCAACCCCAGCTACACTGTGAGCTGAAGTGGCTTCCGAGCAGGAAGTCAAAGCCTCACA----AAGTGGTCTCCAAG-A

Human       GGAGGACCCAGCAGATGTGCAAAGAGA--------GGAACACACTCCTGTGGCCCCAGAGAGAGTGGTGGCCCGGACACCTGCCACCTCCCCTGCCACTC
Chimp       GGAGGACCCAGCAGACGTGCAAAGAGA--------GGAACACACTCCTGCGGCCCCAGAGAGAGTGGTGGCCCGGACACCTGCCACCTCCCCTGCCACTG
Orangutan   GGAGGACCCAGCAGACGTGCAAAGAGAAGAG---AGGAGCACACTCCTGTGGCCCCAGAGAGAGTGGTGGCCTGGACACCTGCCACCTCCCCTGCCACTG
Rhesus      GGAGGACCCAGCAGACATAGAAAGGGGAGAAGACAGGAGCACACGCCTGTGGCCCCAGAGAGAGTGGCAGCCCAGACACC-GCCAGCTCCCC-GCCACTG
Marmoset    GGAGGACCCAGAAGATGTGCAGAGAGA--------GGAGTACGCTCATGTGGCCCCAGAGAGGGCAGCAGCCCGGACACCCGCCAGCTCCCCTGCCATTG

Human       A
Chimp       A
Orangutan   A
Rhesus      A
Marmoset    G
```

S1-12: ENSP00000366205 ENST00000377006 ENSG00000204666 H-C

```
Human       ATGTCACTAGACAGTGGGGCCCTCAAGGACCTGGCAAATGGGGGCTTGGACCTCGGATCGG-CCTCCCACGCGAAGCTTGCTCCCCACCAGCATCCCCAC
Chimp       ATGTCACTAGACAGTCGGGCCCTCAAGGACCTGGCAAATGGGGGCTTGGACCTCGGATCGG-CCTCCCACGCGAAGCTTGCTCCCCACCAGCATCCCCAC
Orangutan   ATGTCACTAGACAGCCGGGCCCTCAAGGACCTGGCAAATGTGGGCTTGGACCTCGGGTCGG-CCTCCCACGCGAAGCTTGCTCCCCACCACCATCCCCAC
Rhesus      ATGTCACTAGACAGTCGGGCCCTCAAGGACCTGGCAACTGGAGGCTTGGACCTCGGGTCGG-CTTCCCACGCGAAGCTTGCTCCCCACCTGCATCCGCAC
Marmoset    GTGTCACTAGACGGTCGGGCCCCCAGGTACCTGGCAACTGGGGCGTTGGAC------TCGGTCCTCCCAGGCGAAGCTCGCTCCCCATCAGCATCCCTAT

Human       GTTGGTGGCGACGCTGCCCCGG-CCCCACGGATACTTCCGCGCCTGTCAGACTCCCTGATGAACTACCCTTCCCAGAGTACCGCGGGAGCTCGGGCTCCT
Chimp       GTTGGTGGCGACGCTGCCCCGG-CCCCACGGATACTTCCGCGCCTGTCAGACTCCCTGATGAACTACCCTTCCCAGAGTACCGCGGGAGCTCGGGCTCCT
Orangutan   GTTGGTGGCGACGCTGCCCCGG-TCCCACGGATACTTCCGCGCCTGTCAGACTCCCTGATGAACTACTCTTCCCAGAGTACCGCGGGAGCTCGGGCTCCT
Rhesus      GTTGGTGGAGACGCTGCCCCGG-CCCCACGGATGCCTCTGCGCATGTCAGACTCCCTGATGAACTACCCTTCCCAGAATCCCGCGGGA------GCTGCT
Marmoset    GTTGGTGGCGAAGCTGCCCCAGCCCCCACGGATGCTTCCGCGCCTGTCAAACTCCGGGATGAACTACCCTTCCCAGAGTCCCGCGGGAGCTCGGGCTCTT

Human       GAGGGCGACGGTCCTCTGATGGCAGATGCGGGAGAAACTCTGGCGTCAGGCGGCCCTCGCGTGGAGCACACGAAGTCGTGGCTTATTCTGGCTTCAGTAT
Chimp       AAGGGCGACGGTCCTCTGATGGCAGATGCGGGAGAAACTCTGGCGTCAGGCGGCCCTCGCGTGGAGCACACGAAGTCGTGGCTTATTCTGGCTTCAGTAT
Orangutan   TAGGGCGACGGTCCTCTGATGGCAGATGCGGGAGAAACT----------------CCTTCGTGGGGCACACGAAGTCGTGGCTTATTTTGGCTTCAGTAT
Rhesus      TAGGGCGACGGTCCTCTGATGGCCGATGCGGGAGAAACCCTGGCGTCAGGCGGCCCTCGAGTGGGGCACACGAAGTCGTGGCTTATTCTGGCTTCAGTAT
Marmoset    TAGGGTGACGGTCCTCTGATGGCAGATGCGGGAACATCCCTGGCGTCAGGCAGCCCTCGCGTGGGGCGCATGAAGCCACGGCTTCCTCTGACCTCGATAG

Human       GTGGGGTGGAGAAGGCGATCCACGCAGCTGCGTCTATTTCCTGTGGATCAATCGCAAAATACGTTCTGTAA
Chimp       GTGGGGTGGAGAAGGCGATCCACGCAGCTGCGTCTATTTCCTGTGGATCAATCGCAGAATACGTTCTGTAA
Orangutan   GTCGGGTGGAGAAGGCGATCCACGCGGTTGCGTCTATTTCCTGTGGATCAATCGCAGAATACATTCTGTAA
Rhesus      GCGGGGTGGAGAAGGCGATCCACGAGGCTGCCTCTGTTTCCTATGGATCAAACGCAGAAATCGTTCTGTAA
Marmoset    GCGGGGTGGAGAAGGCGGCCCGC------GCGTCTATTTCCTAGCGGCCAATCGCAGGATACGTTCTGTAA
```

S1-13: ENSP00000366264 ENST00000377064 ENSG00000204674 H-C

```
Human      ATGCCCCTCCCAGGAACCCCA-------GGACCTGTCACCACCTCCCCACAGACCCCCACCCCCCGCCCCCTCACCACCGACTGGCGCATCTTGTCCGGC
Chimp      ATGCCCCTCCCAGGACCCCCA-------GGACCTGTCACCACCTCCCCACAGACCCCCACCCCCCACCCCCTCACCACCGACTGGCGCATCTTGTCCGGC
Orangutan  ATGCCCCTCCCAGGACCCCCA-------GGACCTGTCACCACCTCCCCACAGACCCCCACCCCC-GCCCCCTCACCACCGACTGGCGCATCTTGTCCGGC
Rhesus     ATGCCCCTCCCAGTACCCCCA-------GAACTTGTCACCACCTCCCCACAGAACCCCACCCCCCGCCCCCTCACCACTGACTGGCGCATCTTGTCCGGC
Marmoset   ACACCTCTCCTGGGACCCCCAACCTGCAGGACCTGCCACCGCCTCCCCACAGAACCACAACTCCTGGCCCCTTACCACTGACTGGCGCATCTTGTCTGGC

Human      AAGGGGTCCGGGGGCTCGGCCCGGGCTGTCTCCAAGCTGCGCTCCTCCAGCTCAGGGAACAGCTTGCTCCGGATCAGAGACCTGGGGGTGAGGAAGAGTC
Chimp      AAGGGGTCCGGGGGCTCGGCCCGGGCTGTCTCCAAGCTGCGCTCCTCCAGCTCAGGGAACAGCTTGCTCCGGATCAGAGACCTGGGGGTGAGGAAGAGTC
Orangutan  AAGGGGTCCGGGGGCTCAGCCCGGGCTGTCTCCAAGCTGCGCTCCTCCAGCTCAGGGAAGAGTTTGCTCCGGATCAGAGACCTGGGGGTGAGAAAGAGTC
Rhesus     AAGGGGTCCGGGGGCTCGGCCCGGGCTGTCTCCAAGCTGCGCTCCTCCAGCTCGGGGAAGAGCTTGCTCCGGATCAGAGACCTGGGGGTGAGGAAGAGTC
Marmoset   AGGGGGTCCGGGGGCTCGGCGCGGGCTGTCTCCAGGCTGCGCTCCTCTAGCTCAGAGAAGAGCTTGCTCCGGATCAGAGACCTGGGGGTGAGGAAGAATC

Human      AGGAGGAGGCCGCCCCTCCTTCCCCCCGGCCCCAGTCA----CGGGCACATGCACAGACCACAAACCCCTACTGGGCAGACACAAACACACGACCCGGAG
Chimp      AGGAGGAGGCCGCCCCTCCTTCCCCCCAGCCCCAGTCA----CGGGCACATGCACAGACCACAAACCCCTACTGGGCAGACACAAACACACGACCCGGAG
Orangutan  AGAAGGAGGACACCCCTCCTTCCCCCCGGCCCCAGTCA----CGGGCACGTGCACAGACCACAAACCCCTACTGTGCAGACACAAACACGAGACCCGGAG
Rhesus     AGGAGGAGGACGCCCCTTCTTCCCC--GGCCCCAGTCA----CAGGCACATGCACAGACCACAAACCCCTACTGTGCAGACACAAACACACGACCCGGAG
Marmoset   AGAAGGAGGACGCCCCTCCCTCCCCCCGGCCCCAGTCAGTCATGGGCACATGCACAGACCACAAATCCCTACCATGCAGACACAAACACAC-ACCCAGAG

Human      CACAC-------------------------------------CCACCAGAGCACGGTGGGGTCGCTGCTCTGCCGGCTCAGGTGGTAGGACAGTGCCACC
Chimp      CACAC-------------------------------------CCACCAGAGCACGGTGGGGTCGCTGCTCTGCCGGCTCAGGTGGTAGGACAGCGCCACC
Orangutan  CACACA-----CACCAGCGCGAGCACAGGCGCGTGCGGGCACCCACCAGAGCACGGTGGGGTCGCTGCTCTGCCGGCTCAGGTGGTAGGACAGCGCTACC
Rhesus     TACACA-----CACCAGCGCGAGCACAGGCGCGTGCGGGAACCCACCAGAGCACGGTGGGGTCGCTGCTCTGCCGGCTCAGGTGGTAGGACAGCGCCACC
Marmoset   CACACAACACACACCAGGGCGAGCACAGGCACGTGCGGGCACCCACCAGAGCACGGTGGGGTCACTGCTCTGCCGGCTCAGGTGGTAGGACAGTGCCACC

Human      AGGAGGCCACAGAAGACTGA
Chimp      AGGAGGCCACAGAAGACTGA
Orangutan  AGGAGGCCACAGAAGACTGA
Rhesus     AGGAGGCCACAGAAGACTGA
Marmoset   AGGAGGCCACAGAAGACCGA
```

S1-14: ENSP00000375249 ENST00000391430 ENSG00000212736 H-C-O

```
Human      ATGCTAAACTGGTTGGCCCAAATCCAACAGATTGCAAAGAGTGGCAGGGCCTGGCAGCTGTCTGGAAGCCCTTCATCTTACAGACAAGGAAGTGGTGTCA
Chimp      ATGCTAAACTGGTTGGCCCAAATCCAACAGATTGCAAAGAGTGGCAGGGCCTGGCAGCTGTCTGGAAGCCCTTCATCTTACAGACAAGGAAGTGGAGTCA
Orangutan  ATGCTAAACTGGTTGGCCCAAATCCAACAGATTGCAAAGAGTGGCAGGGCCTGGCAGCTGTCTGGAAGCCCTTCATCTTACAGACAAGGAAGTGGAGTCA
Rhesus     ATGCTAAACTGGGTAGCCCAAATCCAACAAATTGCAAAGAGTGGCAGGGCCTGGCAGCTGTCTGGAATCCCCTCATCTTACAGACAAGAAAGTGGAGTCA
Marmoset   CTGCTAACCAGGCTGGCCCAAATCCAACAGATTGCAAAGAGTGGCAGGGCCTGGCAGCTGTGTGGAAGCCCTTCATCTTACAGGCAAGGAAGTGGAGTCC

Human      AATGGACAGTAAAGGTGAATCACATACTCAGCTATAGCCTGCTCCCAGCTGGGGCACTCCAGGCCCCTCCTCTGGTCACTACTGTA-GAAATTAACTGCA
Chimp      AATGGACAGTAAAGGTGAATCACATACTCAGCTATAGCCTGCTCCCAGCTGGGGCACTCCAGGCCCCTCCTCTGGTCACTACTGTA-GAAATTAACTGCA
Orangutan  AATGGACAGTAAAGGTGAATCGCATACTCAGCTATAGCCTGCTCCCAGCTGGGGCACTCCAGTCCCCTCCTCTGGTCACTACCATA-GAAATTAACTGCA
Rhesus     AATGGAGATTGAAGGTGAATCGCATACCCAGCTATAGCCTGCTCCCAGCTGGGGTGCTCCAGGCCCCTCCTCTGGTCACTACCATAGGAAATTAGCTGCA
Marmoset   AATGGAGCTTAAAGGCAAACTGCATACCCAGCTACAACCTGCTCCCAGCTGGGGCACTCCAGGCCCCTCCTCTGGTCACAGCCATAGGAAATTAACTGCA

Human      TGTGTCTCAATCTGCGAGTGCAGCTGCTTCTAGATGGTGGCACCTTCCAGAGCTTGGTTCGCCCTGTGTCTACCCAAC-AAGTACAGATGCCATCCCGGT
Chimp      TGTGTCTCAATCTGCGAGTGCAGCTGCTTCTAGATGGTGGCACCTTCCAGAGCTTGGTTCGCCCTGTGTCTACCCAAC-AAGTACAGATGCCATCCCGGT
Orangutan  TGTGTCTCCATCTGCGAGTGCAGCTGCTTCTAGATGGTGGCACCTTCCAGAGCTTGGTTCACCCTGTGTCTACCCAAC-AAGTCTAGATGCCATCCCGGT
Rhesus     TGTGTCTCAATCTGCGAGTGCAGCTCCTTCTGGATGGTGACACCTTCCCCAGCTTGGTTCACCCTGTGGCTACC---C-AAGTCCAGACGCCATCCCGGT
Marmoset   TGTGTCTCAATCTGCAAGTGCAGCTTCTTCTAGATGGTGGCACCTTCCAGAGCTTGGTTCACCCTGTGTCTACCCAACAAAGTCCAGATGCCATCCTGGT

Human      GCTGTGATCTTCCAGCCATTTCTCCATTTCTGTCACAGCCCAGAAAGTGA
Chimp      GCTGTGATCTTCCAGCCATTTCTCCATTTCTGCCACAGCCCAGAAAGTGA
Orangutan  GCTGTGATCTTCCAGCCATTTCTCCATTTCTGCCACAGCCCAGAAAGTGA
Rhesus     GCTGTGATCTTCCAGCCACTTCTCCATTTCTGCCACAGCCCAGAAAGTGA
Marmoset   GCTGTGATCTTCCAGCCATTTCTCCATTTCTGTCACAGTCCAGAAAGTGA
```

S1-15: ENSP00000375688 ENST00000391812 ENSG00000167747 H-C-O

```
Human      ATGACAGTGCTGGAGGCCGTCTTGGAGATCCAGGCCATCACTGGCAGCAGGCTGCTCTCCATGGTGCCAGGGCCCGCCAGGCCACCAGGCTCATGCTGGG
Chimp      ATGACAGTGCTGGAGGCCGTCTTGGAGATCCAGGCCATCACTGACAGCAGGCTGCTCTCCATGGTGCCAGGGCCCGCCAGGCCACCAGGCTTATGCTGGG
Orangutan  ATGACAGTGCTGGAGGCCGTCCTGGAGATCCAGGCCATCACTGACAGCAGGCTGCTCTCCATGGTGCCAGGGCCCGCCAGGCCACCAGGCTCATGCTGGG
Rhesus     ATGACGTTGTTCTAGGCCGTCCTGGACATCCAGGCCATTGCCGACAGCAGGCTGCTTTCCATGGAGCCAG--------AGGCTGCCAGGCTCACGCTAGG
Marmoset   ATGATGGTGCTGGAGGCTGTCCTGGAGATCCAGACCATCACCAACAGCAAGCTCATCTCTATTGTGCCAGGGCCAGCCAGGCCACCAAGCTCACGCTAGG

Human      ACCCAACCCAGTGCACAAGGACTTGGCTGCTGAGCCACACACCCAGGAGAAGGTGGATAAGTGGGCTACCAAGGGCTTCCTGCAGGCTAGGGGAGGAGCC
Chimp      ACCCAACCCAGTGCACAAGGACTTGGCTGCTGAGCCACACACCCAGGAGAAGGTGGATAAGTGGGCTACCAAGGGCTTCCTGCAGGCTAGGGGAGGAGCC
Orangutan  ACCCAACCCAGTGCACAAGGACTTGGCTGCTGAGCCACACACCCAGGAGAAGGTGGATAAGTGGGCTACCAAGGGCTTCCTGCAGGCTGGGGGAGGAGC-
Rhesus     ACCCAACCCAGTGCACAAGGACCTGGCTGCTGAGCCGCACACCCTGGAGAAGGTGGATGAGTGAGCTACCAAGGGCTTCCTGCAGGCTAGGGGAGGAGCC
Marmoset   ACCCAACCCAGTGCACCAGGACCTGGCTGCTGAGCCGAGAGCCCAGGAGAAGGTGGATAAG-------------------------CTGGGGATGGAGCC

Human      ACCCCCGCTTCCCTATTGTGACCAGGCCTATGGGGAGGAGCTGTCCATACGCCACCGTGAGACCTGGGCCTGGCTCTCAAGGACAGACACCGCCTGGCCT
Chimp      ACCCCCGCTTCCCTGTTGTGACCAGGCCTATGGGGAGGAGCTGTCCATACGCCACCATGAGACCTGGGCCTGGCTTTCAAGGACAGACACCGCCTGGCCT
Orangutan  -----------------------------------------TGCCCATACACCACCATGAGACCTGGGCCTGGCTCTCGAGGACAGGCACCACCTGGCCT
Rhesus     AGCC-TGCTCCCCTGTTCTGACCAGGCCTGCGGGGAGGAGCTGCCCATATGCCACCATGAGACCTGGGCCTGGCTCCCGAGGACAGATACCGCCTGGTCT
Marmoset   ACCACTCCCCGCCTATTCTGACCAGACCTGTGGGAAGTAGCTGCCCATGCACCACAGCCAGCCCTGGGCCTGGCTCTGGAGGACAGACACCACCCGGCCT

Human      GGTGCTCCAGGGGTGAAGCAGGC---CAGAATCCTGGGGGAGCTGCTCCTGGTTTGA
Chimp      GGTGCTCCAGAGGTGAAGCAGGC---CAGAATCCTGGGGGAGCTGCTCCTGGTTTGA
Orangutan  GGTGCTCCAGGGGTGAAGCCGGC---CAGAATCCTAGGGGAGCTGCTCCTGGTTTGA
Rhesus     GGTGCTCCAGGGGTGAAGCAGGC---CAGAATCCTGGGGGAGCTGCTCCTGGTTTGA
Marmoset   GGTGCTCCAGGGGTAAAGCAGGGAGGCTGGGTTCTGGGGGAGCTGCTCCTGGTGTGA
```

S1-16: ENSP00000380701 ENST00000397571 ENSG00000214112 H-C-O

```
Human      ATGGAAAAGTGGAAACTTGGCAGACAGATCCAGCCTCCCTGGCCACTGGCCCATGCTCGTGGCTCCTGGATGGCGCTGCC-ACGTTCTGAGCAGCTTGGG
Chimp      ATGGAAAAGTGGAAACTTGGCAGACAGATCCAGCCTCCCTGGCCACTGGCCCATGCTCGTGGCTCCTGGATGGCGCTGCC-ACGTTCTGAGCAGCTTGGG
Orangutan  ATGGAAAAGTGGAAACTTGGCAGACAGATCCAGCCTCCCTGGCCACTGGCCCATGCTCATGGCTCCTGGATGGCGCTCCC-ACGTTCTGAGCAGCTTGGG
Rhesus     ATGGAAAAGTGGAAACTTGGCAGACAGATCCAGCCTCCCTGGCCATTGGCCCATGCTCGTGGCTCCTGGATGGTGCTGCCCACGTTCTGAGCAGCTCGGG
Marmoset   ATGGAAGCGTGGAAACTTGGCAGGCAGATCCAGCCTCCCTGGCCGCTGGCCCATGCTCGTGGCTCCCGGATGGCGCTGCCTGTATTCCGGGCAGCTCGGG

Human      ACAGGTGGAGATCAGGACTGGCAGCTGCAAGGACACACCAG-----AGCCACAGAAACTAAAGAGAATTTCCAAAAGGAGTCTATGGTGAAGTCTCTGAG
Chimp      ACAGGTGGAGATCAGGACTGGCAGCTGCAAGGACACACCAG-----AGCCACAGAAACTAAAGAGAATTTCCAAAAGGAGTCTATGGTGAAGTCTCTGAG
Orangutan  ACAGGTGGAGATCAGGACTGGCAGCTGCAAGGACACACCAG-----AACCACAGAAACTAAAGAGAATTTCCAAAAGGAGTCTATGGTGAAGTCTCTGAT
Rhesus     ACAGGTGGAGATCAGGACTGGCAGCTGCAAGGACAGACCAG-----AGCCACAGAAACTAAAGAGAATTTCCAAAAGGAGGCTATGGTGAAGTCTCTGAT
Marmoset   ACAGGTGGAGATCAGGACTGGCAGCTGCAAGG-CAGACCAG-----AGACACAGAAACTAAAGGGAATTTCCAGGAGGAGGCTATGGTGAAGTCTCTGAT

Human      GATGCAAAGAAGACAAGGAGAATGA
Chimp      GATGCAAAGAAGACAAGGAGAATGA
Orangutan  GATGCAAAGAAGA-AAGGAGAATGA
Rhesus     GATGCAAAGAAAACAAGGAGAATGG
Marmoset   GATGCAAAGAAGACAAGGAGAATGA
```

S1-17: ENSP00000380733 ENST00000397608 ENSG00000214130 H-C

```
Human      ATGGCCATGGCCCACGCAGGGCTCTGCGGGTGGAGAGGGGAAGTCAGCCTAGACTCCAGAGAGAAACCCTGGGGCAACCCGAAAGGCCTGAGGAAAACTG
Chimp      ATGGCCATGGCCCACGCAGGGCTCTGCGGGTGGAGAGGGGAAGTCAGCCTAGACTCCAGAGAGAAACCCTGGGGCAACCCGAAAGGCCTGAGGAAAACTG
Orangutan  ATGGCCATGGCCCACGCAGGGCTCTGCGGGTGGAGAGGGG-AGTCAGCCTAGACTCCAGAGAGAAACCCTGGGGCAACCCGAAAGGCCTGCGGAAAACTG
Rhesus     ATGGCCATGGCCCAAGCAGGGCTCAGAGGGTGGAGAGGGG-AGTCAACCTAGACTCCAGAGAGAAACCTTGGGACAACCCTAAAGGCCTTCTGAAAACTG
Marmoset   AGGGCCACTGCCCGCACAGGGCTCTGCGGGTCGGGTGGGG-AGTCAGCCTAGATTCCAGAGACAAACCCCGGGGCCACCCGAAAGGCCTGAGGAAAACTG

Human      GAATTGGGGTTAGGCATGAGGGGAGGGAGTCTCTGGGGAAAACGGGCCGGGCTGTGCGGAGCCCTGAGGGCTCCTGCGGCTGCAGCGCTGCAGGCCGC--
Chimp      GAATTGGGGTTAGGCATGAGGGGAGGGAGGCTCTGGGGAAAACGGGCCGGGCTGTGCGGAGCCCTGAGGGCTCCTGCGGCTGCAGCGCTGCAGGCCGC--
Orangutan  GAATTGGGGTTAGGTATGAGGGGAGTGAGGCTCTGGGGAAAACGGGCCGGGCTGTGCAGAGCCCTGAAGGCTCCTGCGGCTGCAGCGCTGCAGGCCGC--
Rhesus     GAACT-GGGTTAGGCACGAGGGGTGAGAGACTCTGGGGAAAACCGGCCGGGCT-TGCGGAGCCCTGAGGGCTCCTGCGGCTGCAGCGCTGCAGACCGC--
Marmoset   GAATTGGGGTTAGGCTTGAGGGGAGGGAGGCTCTGGGAAAAACCGGCCCGGCTGTGCGAAGCACCAAGGGTTCCTGTGGCTGCAGCGCTGCAGGCCTCCC

Human      ------------CCGCGTCT-----CGCCCCGCCCCGCCGCTGGGCGTCCCCGCCAGGAGCCCACCCGCGGGCGGCAGCTTTTCTCCATGCTGCCCAGGG
Chimp      ------------CCGCGTCT-----CGCCCCGCCCCGCCGCTGGGCGTCCCCGACAGGAGCCCACCCGCGGGCGGCAGCTTTTCTCCATGCTGCCCAGGG
Orangutan  ------------CCCCGTCTCGCCCCGCCCCGCCCCGCCGCTGGGCGTCCCCGCCAGGAGCCCACCCGCGGGCGGCAGCTTTTTTCCATGCTGCCCAGGG
Rhesus     ------------CCCTGTCT-----CGCCCCGCCCCGCCGCTGGGCGTCCCCGCCAGCAGCCCACCCGCGGGCGGCAGCTTTTCTCCATCCTGCCCAGGG
Marmoset   TGCCCCGCCCCGCCCTGCCC-----CGCCCCGCTCCGCCGCCGGGCGTCCCC-CCAGGAGCCCACCCGCGGGCAGCAGCTTTTCGCCATCCTGCCCAGGG

Human      AAGTTCGATGCCT-GGTGCTGGGATGCGCCAGCGCTTCTGTTCGCTTCTGGCAATCCTGGCGTCTCCCAATGAGAGGGCTCTCAAAATGAAGCTTTTAAT
Chimp      AAGTTCGATGCCT-GGTGCTGGGATGCGCCAGCGCTTCTGTTCGCTTCTGGCAATCCTGGCGTCTCCCAATGAGAGGGCTCTCAAAATGAAGCTTTTAAT
Orangutan  AAGTTCGATGACT-GGTGCTGGGATGCGCCAGCGCTTCTGTTCGCTTCTGACAATCCTGGCGTCTCCCAATGAGAGGGCTCTCAAAATGAAGCTTTTAAT
Rhesus     AAGTTCGATGCCT-GGTGCTGGGATGCGCCAGCGCTTCTCTTCGCTTCTGACAATCCTGCCGTTTCCGATTGAGGGGGCTCTCAAAATGAAGCTTTTAAT
Marmoset   AAGTTCGATGCCCGGGTGCTGGGACGCGCCAGCGATTCTCTTCTTTCCTGGCAATTCTGGCGTCTCCGATTCAGAGGGCTCTCAAAATGAAGCTTTTAAT

Human      AAACTCCAGAGTAAGGAACTCGGGATTGTGCGGCAAAGGCCGCGCATTGCACTTTGTGAGCAATCGGTAA
Chimp      AAACTCCAGAGTAAGGAACTCGGGACTGTGCGGCAAAGGCCGCGCATTGCACTTTGTGAGCAATCGGTAA
Orangutan  AAACTCCAGAGTAAGGAACTCGGGATTGTGCGGCAAAGGCCGCGCATTGCACTTTGTGAGCAATCGGTAA
Rhesus     AAACTCCAGAGTAATGAACTCGGGAT--TGCGGCAAAGGCCGCGCATTGCAATTTGTGAGCAATCGATAA
Marmoset   AAACTCCAGAGTAAGGGACTCGGGATTGTGCGGCAAAGGTAGCGCATTGCACTTTGGGGGCAATCGGTAA
```

S1-18: ENSP00000382022 ENST00000399070 ENSG00000118267 H

```
Human      ATGTGTGTGGGAAAGCCTTCAGTCAGAGCTCAGATCTTATTCTGCATCAGAGAATCCATACTGGGGAGAAACCATATCCATGTAATCAGTGTAGCAAAAG
Chimp      ATGTGTGTGGGAAAGCCTTCAGTCAGAGCTCAGATCTTATTCTGCATCAGAGAATCCATACTGGGGAGAAACCATATCCATGTAATCAGTGTAGCAAAAG
Orangutan  ATGTGTGTGCGAAAGCCTTCAGTCAGAGCTCAGATCTTATTCTGCATCAGAGAATTCACAGTGGGGAGAAACCATATCCATGTAATCAGTGTAGCAAAAG
Rhesus     ATGTGTGTGGGAAAGCCTTCAGTCAGAGCTCAGATCTTATTCTGCATCAGAGAATCCACACTGGGGAGAAACCATATCCATGTAATCAGTGTAGCAAAAG
Marmoset   ATGTGTGTGGGAAAGCCTTCAGTCAGAGCTCAGATCTTATTCTGCATCAGAGAATCCACAATGGGAAGAAACCATATCCATGTAATCAGTGTGGCAAAAG

Human      TTTCAGTCAGAATTCAGACCTTATTAAACATCGAAGGATCCACACTGGAGAGAAACCCTATAAATGTAATGAGTGTGGGAAAGCTTTTAATCAGAGCTCA
Chimp      TTTCAGTCAGAATTCAGACCTTATTAAACATCGAAGGATCCACACTGGAGAGAAACCCTATAAATGTAATGAGTGTGGGAAAGCTTTTAATCAGAGCTCA
Orangutan  TTTCAGTCAGAATTCAGACCTTATTAAACATCGAAGGATCCACACTGGAGAGAAACCCTATAAATGTAATGAATGTGGGAAAGCTTTTAATCAGAGCTCA
Rhesus     TTTCAGTCAGAATTCAGACCTTATTAAACATCAAAGGATCCACACTGGAGAGAAACCCTATAAATGTAATGAATGTGGGAAAGCTTTTAATCAGAGCTCA
Marmoset   TTTCAGTCAGAATTCAGACTTTATTAAACATCAAAGGGTCCACACTGGAGAGAAATCCTATAAATGTCATGAATATGGGAAAGCTTTTAATCAGAACTCA

Human      GTCCTTATTTTACATCAGAGGATTCATACTGGAGAGAAACCCTATCCCTGTGATCAATGTAGCAAAACCTTCAGTAGGCTTTCAGATCTTATTAATCATC
Chimp      GTCCTTATTTTACATCAGAGGATTCATACTGGAGAGAAACCCTATCCCTGTGATCAATGTAGCAAAACCTTCAGTAGGCTTTCAGATCTTATTAATCATC
Orangutan  GTCCTTATTTTACATCAGAGGATTCATACTGGAGAGAAACCCTATCCCTGTGATCAATGTAGCAAAACCTTCAGTAGGCTTTCAGATCTTATTAATCATC
Rhesus     GTCCTTATTTTACATCAGAGGATTCATACTGGAGAGAAACCCTATCCTTGTGATCAATGTAGCAAAACCTTCAGTAGGCTTTCAGATCTTATTAATCATC
Marmoset   GTCCTTATTTTACATCAGAGAATTCATATTGGAGAGAAACCCTATTTCTGTGATCAATGTAGCAAAACCTTCAGAAGACTTTCAGATCTTATTAATCATC

Human      AACGAATTCACACTGGAGAGAAGCCTTACCCATGTAATCAGTGCAATAAAATGTTTAGTCGAAGATGAGATCTTGTTAAACATCACAGAATTCATACAGG
Chimp      AACGAATTCACACTGGAGAGAAGCCTTACCCATGTAATCAGTGCAATAAAATGTTTAGTCGAAGATCAGATCTTGTTAAACATCACAGAATTCATACAGG
Orangutan  AACGAATTCACACTGGAGAGAAGCCTTACCCATGTAATCAGTGCAATAAAATGTTTAGTCGAAGATCAGATCTTGTTAAACATCACAGAATTCATACAGG
Rhesus     AACGAATTCACACTGGAGAGAAGCCTTACCCATGTAATCAGTGCAATAAAATGTTTAGTCGAAGATCGGATCTTGTTAAACATCACAGAATTCATACAGG
Marmoset   AACGAATTCAGACTGGAGAGAGGCCTTACTCATGTAATCAGTGCAATAAAATGTTTTGTCAAAGATCAGATCTTGTTAAACATCAGAGAATTCATACAGG

Human      TGAGAAACCCTATGAATGTGATGAATGTGGGAAAACCTTTAGTCAGAGCTCCAACCTTATTCTTCATCAGAGAATCCACACTGGAGAGAAACCTTATGCA
Chimp      TGAGAAACCCTATGAATGTGATGAATGTGGGAAAACCTTTAGTCAGAGCTCCAACCTTATTCTTCATCAGAGAATCCACACTGGAGAGAAACCTTATGCA
Orangutan  TGAGAAACCCTATGAATGTGATGAATGTGGGAAAACCTTTAGTCAGAGCTCCAACCTTATTCTTCATCAGAGAATCCACACTGGAGAGAAACCTTATCCA
Rhesus     TGAGAAACCCTATGAATGTGATGAATGTGGGAAAACCTTTAGTCAGAGCTCCAACCTTATTCTTCATCAGAGAATCCACACTGGAGAGAAACCTTATCCA
Marmoset   TGAGAAACCCTATGAATGTGATGAATGTGGGAAAGCCTTTAGTCAGAGCTCCAACCTCATTTTTCATCAGAGAATCCACACTGGAGAGAAACCTTATCCC

Human      TGTAGTGATTGTACTAAAAGCTTTAGTCGCCGTTCAGATCTTGTTAAGCATCAAAGAATACACACTGGAGAGAAACCATATGCATGTAATCAGTGTGATA
Chimp      TGTAGTGATTGTACTAAAAGCTTTAGTCGCCGTTCAGATCTTGTTAAGCATCAAAGAATACACACTGGAGAGAAACCATATGCATGTAATCAGTGTGATA
Orangutan  TGTAGTGATTGTACTAAAAGCTTTAGTCGCCGTTCAGATCTTGTTAAGCATCAAAGAATACACACTGGAGAGAAACCATATGCATGTAATCAGTGTGATA
Rhesus     TGTAGTGATTGTACTAAAAGCTTTAGTCGCCGTTCAGATCTTGTTAAGCATCAAAGAATACACACTGGAGAGAAACCATATGCATGTAATCAGTGTGATA
Marmoset   TGTAGGGATTGTACTAAAAGCTTTAGTCACGGTCCAGATCTTGTTAAGCATCAAAGAATACACACTGGAGAGAAACCATATGCATGGAATCATTGTGATA

Human      AAAGTTTTAGTCAAAGCTCAGACCTCACTAAACATCAGAGAGTACACTCTGGTGAAAAGCCTTATCATTGCAATAGTTGTGAGAAAGCCTTCAGTCAGAG
Chimp      AAAGTTTTAGTCAAAGCTCAGACCTCACTAAACATCAGAGAGTACACTCTGGTGAAAAGCCTTATCATTGCAATAGTTGTGAGAAAGCCTTCAGTCAGAG
Orangutan  AAAGTTTTAGTCAAAGCTCAGACCTCACTAAACATCAGAGAGTACACTCTGGTGAAAAGCCTTATCATTGCAATAGTTGTGAGAAAGCCTTCAGTCAGAG
Rhesus     AAACTTTTAGTCAAAGCTCAGACCTCACTAAACATCAGAGAGTACACTCTGGTGAAAAGCCTTATCATTGCAATAGTTGTAAGAAAGCCTTCAGTCAGAG
Marmoset   AAAGATACAGTCAAAGCTCAGACCTCACTAAACATCAGAGAGTACACTCTTATGAAAAGCCTTATCATTGTAATAGTTGTGGGAAAGCCTTCAGTGAGAG

Human      TTCTGACCTTATTCTTCATCAGAGAATTCACACTGGAGAAAAACTATTATCTGTGCACACAGTGCAGCAAAAGTTTCAGTCAGATCTCAGACCTCATTAA
Chimp      TTCTGACCTTATTCTTCATCAGAGAATTCACACTGGAGAAAAACCAT-ATCTGTGCACACAGTGCAGCAAAAGTTTCAGTCAGATCTCAGACCTCATTAA
Orangutan  TTCTGACCTTATTCTTCATCAGAGAATTCACACTGGAGAAAAACCAT-ATCTATGCACACAGTGCAGCAAAAGTTTCAGTCAGAACTCAGACCTCATTAA
Rhesus     TTCTGACCTTATTCTTCATCAGAGAATTCACACTGGAGAAAAATCAT-ATCCATGCACACAGTGCAGCAAAAGTTTCATTCAGAACTCAGACCTCATTAA
Marmoset   TTTTGACCTTATTCTTCATCAGAGACTCTACAATGCAGAAAAACCAT-ATCCATGCACACAGTGCAGCAAAAGGTTCAGTCACAACTCATATCTCATTAA

Human      ACACCAGAGAATCCACACTGGGGAAAAACCATATAAATGCAGTGAGTGCAGGAAGGCTTTCAGTCAGTGCTCAGCTCTTACCCTACACCAGAGAATCCAC
Chimp      ACACCAGAGAATCCACACTGGGGAAAAACCATATAAATGCAGTGAGTGCAGGAAGGCTTTCAGTCAGTGCTCAGCTCTTACCCTACACCAGAGAATCCAC
Orangutan  ACACCAGAGAATCCACACTGGGGAAAAACCATATAAATGTAGTGAGTGCAGGAAGGCTTTCAGTCAGTGCTCAGCTCTTACCCTACACCAGAGAATCCAC
Rhesus     ACACCAGAGAATCCACACTGGGGAAAAACCATATAAATGCAGTGAGTGCAGGAAGGCTTTCAGTCAGTGCTCAGCTCTTACTGTACACCAGAGAATCCAC
Marmoset   ACATCAGAGAATACACACTAGGGAAAAACCATATAAATGTAATGAGTACAGGAAGGCTTTCAGTCAGTGCTTAGCCC------TACACTAGAGAATCCAC

Human      ACTGGGAAGAAACCAAATCCATGTGATGAGTGTGGCAAAAGCTTTAGTCGGCGTTCTGATCTCATTAACCATCAAAAAATACACACT-----GGTGAAAA
Chimp      ACTGGGGAGAAACCAAATCCATGTGATGAGTGTGGCAAAAGCTTTAGTCGGCGTTCTGATCTCATTAACCATCAAAAAATACACACT-----GGTGAAAA
Orangutan  ACTGGGGAGAAACCAAATCCATGTGATGAGTGTGGCAAAAGCTTTAGTCGGCATTCTGATCTCATTAACCATCAAAAAATACACACT-----GGTGAAAA
Rhesus     ACTGGGGAGAAACCAAATCCATGTGATGAGTGTGGCAAAAGCTTTAGTCGGCGTTCTGATCTCATTAACCATCAAAAAATACACACT-----GGTGAAAA
Marmoset   ACTGGTGAGAAACCAAATCCATGTGATGAATGTGGCAAAAGCTTTAGTCGGCACTCTGATCTCATTAATAATTGAAAAATACACACTGGTGAGGTGAAAA

Human      GCCGTATAAGTGTGATGCATGTGGGAAAGCCTTCAGCACATGTACTGATCTTATTGAACACCAGAAAACCCATGCTGAGGAGAAACCCTACCAGTGTGTT
Chimp      GCCGTATAAGTGTGATGCATGTGGGAAAGCCTTTAGCACATGTACTGATCTTATTGAACACCAGAAAATCCATGCTGAGGAGAAACCCTACCAGTGTGTT
Orangutan  GCCGTATAAGTGTGATGCATGTGGGAAAGCCTTTAGCACATGCACAGATCTTATTGAACACCAGAAAATCCATGCTGGGGAGAAACCCTACCGTTGTGTT
Rhesus     GCCGTATAAGTGTGAGGCACGTGGGAAAGCCTTTAGCACATGCACAGATCTTATTGAACACCAGAAAATCCATGCTGGGGAGAAACCTTACCAGTGTGTT
Marmoset   GCCATGTAAGTGTGATGCATGTGGGAAAGCCTTTAGCACATGCACAGATCTAATTGAGCACCAGAAAATCCATGTTGGGGAGAAAC---ACCAGTATGTT

Human      CAGTGCAGCAGAAGTTGTAGCCAACTCTCTGAACTTACTATTCATGAGGAAGTCCATTGTGGAGAAGACAGTCAAAATGTGATGAATGTGAGAAAACCTT
Chimp      CAGTGCAGCAGAAGTTGTAGCCAACTCTCTGAACTTACTATTCATGAGGAAGTCCATTGTGGAGAAGACAGTCAAAATGTGATGAATGTGAGAAAACCTT
Orangutan  CAATGCAGCAGAAGTTTTAGCCAACTCTCAGAACTTACTATTCATGAGGAAGTCCATTGTGGAGAAGACAGTCAAAATGTGATGAATGTGAGAAAACCTT
Rhesus     CAGTGCAGCAGAAGTTTTAGCCAACTCTCTGAACTTACTATTCATGAGGAAGTCCATTGTGGGGAAGACAGTTAAAATGTGATGAATGTGAGAAAACCTT
Marmoset   CAGTACAGCAGAAGTTTTAGCCAACTCTTTGAACTTACTAATCATGAGGAAGACCACTGTGGGGAATACAGTCAAAATGTGATGAATGTGAGAAAACCTT

Human      TAGTGTGTACACCAACTCTATTCAGTACCAGAGACACTGTACCAGAAAAAAATCTAATGAATGCTGTTGATTATTGA
Chimp      TAGTGTATACACCAACTCTATTCAGTACCAGAGACACCGTACCAGGAAAAAATCTAATGAATGCTGTTGATTATTGA
Orangutan  TAGTGTATACATCAACTCTATTCAGTACCAGAGACACTGTACCAGGAAAAAATCTAATGAATGCTGTTGATTATTGA
Rhesus     TAGTATATACTTCAACTCTATTCAGTACCAGAGACACCATACCAGGAAAAAATCTAATGAATGCTGTTCATTATTGA
Marmoset   TAGTGCATACGTCATCTCTATTCAGTACCAGAGATACCATACTAGGAAAAAATCTAATGAATGCTGTTGATTATTGG
```

S1-19: ENSP00000383235 ENST00000400385 ENSG00000215458 H

```
Human      ATGCCATGCAGACTTCTACATCAAAGGGAAACAAGGTCAGGAGGGCCGAGGGGTCCACGGCACTCCGCACCCACGGGGCCAGGCTGGCACAACG------
Chimp      ATGCCATGCAGACTTCTACATCAAAGGGAAACAAGGTCGGGAGGGCCGAGGGGTCCATGGCACTCCGCACCCACGGGGCCAGGCTGGCACAACG------
Orangutan  ATGCCATGCAGACTTCTACATCAAAGGGAAACGAGGTCGGGAGGGCCGAGGGGTCCACAGCACTCTGCACCCACGGGGCCAGGCTGGCGGAATG------
Rhesus     ATGCCATGCAGCCTTCTATGTCAAAGGGAAACGAGGTCGGGAGGACCGAGGGGTCCACAGCACTTCGCACCCACGGGGCCAGGCTGGCACA---------
Marmoset   ATGCCATGCAGGCTTGTATGTCAAAAGGAATCGAGGTCAGGAAGCCAGAGGGATCCACAGCACTGCAAACCCATGAGGCCAGGCTGGCGCCACACCCCAT

Human      CCCCAACGCTGCAATCCTGGGAAGAGTCACACGCGCCCTCCCGGGACCCACGTGACCATCAAGGGAG-----------TGTGGAGGACACATCCTCGGG
Chimp      CCCCAACGCTGCAATCCTGGGAAGAGTCACACGCGCCCTCCCGGGACCCACGTGACCATCAAGGGAG-----------TGTGGAGGACACATCCTCGGG
Orangutan  CCCCAATGCTGCAACCCTGGGGAGAGTCACACGCGCCCTCCCGGGACCCACGTGACCGTCAAGGGAG-----------TGTGGCGGACACATCCTCAGG
Rhesus     CCCCAACGCTGCAACCCTGGGAAGAGTCACACGTGCCCTCCCGGGACCCACATGACTGTCGAGGGAGCC----TGGTGTGGGGCAGACACATCTCTCAGG
Marmoset   CCCCATCGCTGCAAACCCAGGAAGAGTCATAGGCTCCCTCCCAGGACCCACGTAACCGCCAAGGGACCCAGCGTGGCATGGAGCGGAGGCAGCCCTTGGG

Human      GGTGACGCCCCTGCAGATGGAGTTTCCCCATCTGTGCCACCCTTGCAAGGCCTGGGAAA-GGCTGCGGGGCCAGGCGGCACCGGGCAAGCTGAGTGCCAG
Chimp      GGTGACGCCCCTGCAGATGGAGTTTCCCCATCTGTGCCACCCTTGCAAGGCCTGGGAAA-GGCTGCGGGGCCAGGCGGCACCGGGCAAGCTGAGTGCCAG
Orangutan  G-TGACACCCCTGCAGATGGAGTTTCCCCATCTGTGCCACCCTTGCAAGGCCTAAGAAA-GGCTGCGGGGCCAGGCGGCACCAGGCAAGCTGAGTGCCAG
Rhesus     GGTGACGCCCCTGCAGATGGAGTTTCCCCATCTGTGCCGCCCTTGTGAGGCCTGGAAAA-GGCTGCAGGGCCAGGCGGCACCGGGCAAGCTGAGTGCCAG
Marmoset   GGCGACGCCC-TGCTGATGGAGTTTTCCCATCTGTGCTTCCCTTGTGAGATCTGGGAAAAGGCTGCCGGGCCAGGCAGCACCGGGCAAGCTGAGTGCCAG

Human      GTGGT--GGTTGCAACACACCGGGCAAATCTGAAACCATGGG-ACGGACGAGCTGCACCCCTGGGAAAGAGAACAGAGGAAGGCCGGTTATCAACGTCCT
Chimp      GTGGT--CGTTGCAACACACCGGGCAAATCTGAAACCATGGG-ACGGACGAGCTGCACCCCTGGGAAAGAGGACAGAGGGAGGCCGGTTATCAACGTCCT
Orangutan  GTGGT--TGTTGCAACATGCTGGGCAAATCTGAAACCATGGGGACGGACGAGCTGCACCCCTGGGAAAGAGGACAGAGGGAGGCCAGTTATCAACGTCCT
Rhesus     GCAGT--TGTTGCAACATGCTGGGAAAATCTGAAACCCTGGGGACGGACGAGCTGCACCCCTGGGAAAGAGGACAGCGGGAGGCCGGTTATCAACGTCCT
Marmoset   GCAGTCTTGCAACAACACGCCAGGCGAATCTGAAACCACGGG-ATGGATGAGCCGCACCCCTGGGAAAGAGAACAGAGGGAGGC-AGTTATCAATGTCCT

Human      CCTGTGCCTCAGTTTCCAGAAACAAGCCAGACTCAGTGCCTCAGGGAGACCCCGTGTGGCCTCCGGAGAGCACGGGACCCGCCCTTTGTGCAGGTGAGGA
Chimp      CCTGTGCCTCAGTTTCCAGAAACAAGCCAGACTCAGTGCCTCAGGGAGACCCCGTGTGGCCTCCGGAGAGCACGGGACCCGTCCTTTGTGCAGGTGAGGA
Orangutan  CCTGTGCCTCGGTTTCCAGAAACAAGCCAGACTCAGTGCCTCAGGGAGACCCCGTGTGGCCTCCGGAGAGCACGGGACCCGCCCTTTGTGCAGGTGACGA
Rhesus     CCTGTGCCTCAGTTTCCAGAAATAAGTCAGACTCAGTGCCCCAGGGAGGCCCCGTGTGGCCTCTGGAGAGCGCAGGACCTGCCCTTTGTGCAGGTGAGGA
Marmoset   CCTGCTCCTTGGTTTTCAGAAATAAGCCAGACTCAGTGCCTCAGGGACACCCCGTGTGGCCTCCGGAGAGCACAGGCCCCACCCTCTGTGCAGGTGAGGA

Human      A-ACTGAACC----------CCAGAGCAGTGAGGGACT----GGCCTGGGGACCCTGGGCTCAGCCCTGGGCGCCTTCCCTCTGCCCATCTCAGAC--GG
Chimp      A-ACCGAACC----------CCAGAGCAGTGAGGGACT----GGCCTGGAGACCCTGGGCTCAGCCCTGGGCGCCTTCCCTCTGCCCATCTCAGACAGGG
Orangutan  A-ACCAAACC----------CCAGAGCAGTGAGGGACT----GGACCGGGGACCCTGGGCTCAGCCCCGGGCGCCTTCCCTCTGTCCATCTCAGACAGGC
Rhesus     A-ACCAAACC----------CCAGAGCAGTGAGGGACT----GGCCTGGAGACCCTGG-CTCAGCCCCAGGTGCCTTCCCTCTGCCCATCTCAGACAGGG
Marmoset   AGCCCAGAGCAATGANNNNNCCAGAGCAATGAGGGACTCACCGGCCTGGGGACTCGAG-CGCAGTCCTGGGCGCCTTCCCTCTGCCCATCTCAGACAGGA

Human      GCACAGCCAGCACCGCCTCACCCCAACGCGCCTCCAGACTGGCCCTCCAGGGGCCCCCAGG-CACAATCCTCTCACTTTCTTCCTCAAGCCCCTG-CCTT
Chimp      GCACAGCCAGCACCGCCTCACCCAAACGCGCCTCCAGACTGGCCCTCCGGGGGCCCCCAGG-CACAATCCTCTCACTTTCCTCCTCAAGCCCCCG-CCTT
Orangutan  GCACAGCCAGCACCGCCTCACCCCAACGCGCCTCTAGACTGGCCCTCCAGGGGCCCCCGGGGAACAATCCTCTCACTTTCCTCCTCAAGCCCCCG-CCCT
Rhesus     GCACAGCCAGCACCGCCTCACCCCA-CACGCCTCCAGATTGGCCCTCAGGGG--CCTTGGG-CACACTCCTCTCTCTCTCCTCCTCAAGCCCCCA-CCCT
Marmoset   GCACGGCCAGCACCACCTCGCCCCAACACGCCTCCAGACCAGCCCTCAGG----CCCCGGG-CACACTCTTCTCACTTCCCTCCTCCAGCCCCCTTCCCT

Human      CCCCCTTCGCACTGTGACCCAGGGGCTGCTAGCAGCTGGGCTGGGCTCCAAAGCTTGAAGCTGCTTCTCCAGG-----TCTCTTTCCAGGGGGCGGCGGA
Chimp      CCCCCTTCGCACTGTGACCCAGGGGCTACTAGCAGCTGGGCCGGGCTCCAAAGCTTGAAGCTGCTTCTCCCGG-----TCTCTTTCCAGGGGGCGGCGGA
Orangutan  TCCCCTTCGCACCGTGACCCAGGGACTGCTAGCAGCTGGGCGGGGCTCCAAGGCTTGAAGCTGCTTCTCCAGG-----TCTCTTTCCAGGGGGCGGCGGA
Rhesus     TCCCCTTCGCACCATGACCCAGGGGCTGCTAGCAGCTGGGCGAGACTCCAAGACTTGAAGCTGCTTTTCCAAGCTGCCTCTCTTTCCAGGGGGCGGCAGG
Marmoset   TCCCCTTCGCATCATCACCCAGGGGCTTCTAGGAGCTGAGCGGGGCTATGAGGCTTGAGGCTGCTGCTCCAGA-----TCCCTTTCCGGGGGGTGGCAGG

Human      TGGCTGCTCACTCAGGGACGCCAACACCAACAGGAAGGGACCCATGCACGGATCTGACTTCCCTCCCAGGCTCTGCCTTCACTCAATGTCCCTGTGGGGC
Chimp      TGGCTGCTCACTCAGGGATGCCAACACCAACAGGACGGGACCCATGTATGGATCTGACTTCCCTCCCAGGCTCTGCCTTCACTCAATGTCCCTGTGGGGC
Orangutan  TGGCTGCTCACTCAGGGACGCCAACACCAACAAGGTGGGACCCATGCACGGATCTGACTTCCCTTCCAGGCTCTGCCTTCACTCAATGTCCCTGTGGGGC
Rhesus     CGGCTGCTCACTCAGGGACGCCGGCACCAACAGGACGGGGCCCATGTGCAGGTCTGACTTTCCTCCCAGGCTCTGCCTTCACCCAATGGCCCTGTGGGGC
Marmoset   TGGCTGCTCAC-CAGGGACACCAGCCCGAACAGGATGGGGCTGATGCGAGGCTCTGACTTTCCTCCCAGGCTCTGCCTTCACCCAATGGTCCTGTGGGGC

Human      AGCTGGGGCTCCCGGCCGCCCTCCCCTGCCCACAGCCGTGAGGTTGCTTCCTAG
Chimp      AGCTGGGGCTCCCGGCCGCCCTCCCCTGCCCACAGCCGTGAGGTTGCTTCCTAG
Orangutan  AGCTGGGGATCCCCGCCGCCCTCCCCTGCCCACAGCCGTGAGGTTTCTTCCTAG
Rhesus     AGCTGGGGCTCCCGGCTGCCCTCCCCTGCCCACAGCCGAGAGGCTTCTTCCCAG
Marmoset   AGCTGGCGCTCCCCGCCGCCCCCTCCC-CCAACAGCTGTGAGGTTTCTTCCTAG
```

S1-20: ENSP00000383299 ENST00000400449 ENSG00000215494 H

```
Human      ATGCCTTCCCAGCCGCACCTCA----GTGCGTGTTCTGTTGAATCTCCGGGTGCTGCAGCCAGGTTGACACAGCGACACCTCACAGGTGTTGCTCGTGGA
Chimp      ATGCCTTCCCAGCCGCACCTCA----GTGCGTGTTCTGTTGAATCTCCGGGTGCTGCGGCCAGGTTGACACAGCGACACCTCACAGGTGTTGCTCGTGGA
Orangutan  ATGCCTTCCCAGCCGCACCTCA----GTGTGTGTTCTGTTGAATCTCCGGGTGCTGCGGCCAGGTTGACACAGCGACACCTCACAGGCGTTGCTCGTGGA
Rhesus     ATGCCTTCCCAGCCGCATCTCA----GTTCATGTTCTGTTGAATCTCTGGGTGCTGCGGCCAGGTTGACACAGCGACACCTCACAGGC-TTGCTTATCGA
Marmoset   GTGCCCTCCCAGCCACACCTCACTCAGTTCCTGTTCTGTTGCATCTCCGGGGGCTGCGGCCAGGTTGACACAGCGACACCTCGCAGGCGTTGCTCGTCGA

Human      GGTGGGCAGACCGGTACATTCCATATGGGGTTACTGCCCAGCAGACAAGAGTGTGCTCCTGTCTCAGCCTTGACCCTCACAACCACCATGGGGCGGCTAT
Chimp      GGTGGGCAGACCGGTACATTCCATATGGGGTTACTGCCCAGCAGACAAGAGTGTGCTCCTGTCTCAGCCTCGACCCTCACAACCACCATGGGGCTGCTAT
Orangutan  GGTGGGCAGACTGGTACATTCCATATGGGGTTACTGCC-AGCAGACAAGAGCATGCTCCCGTCTCAGCCTCGACCCTCACAACCACCATGGGGCGGCTAT
Rhesus     GGTGGGCAGACTGGTACATTCCATACGGGGTTACTGCCCAATAGACAAGAGCGTGCTCCAGTCTCAGCCCCGTCCCTCGCAACCACCGTGGGGCGGCTAT
Marmoset   GGTGGGCAGACAAGTACATTCCATATGGGGTCACTGCCCAGGAGACAGAAGCATGCTCCCATGTCAGCTCTGACCCTCACAACCACTGTGGGGCGGCTGT

Human      TTAGTGTGCACTGGCAAATCTGGGGGCCCTGTTCTCCCTGCATCGTTTACCCCAAAGGTGATAGTGAACAATCTGCTTCCAAGTCGAAACAGGCTTTCCC
Chimp      TTAGTGTGCACTGGCAAATCTGGGGGCCCTGTTCTCCCTGCATCGTTTACCCCAAAGGTGATAGTGAACAATCTGCTTCCAAGTCGAGACAGGCTTTCCC
Orangutan  TTAGTGTGCACTGGCAAATCTGGGG-CCCTGTTCTCCCTGCATCGTTTACCCCAAAGGTGATAGTGAACAATCTGCTTCCAAGTCGAGATAGGCTTTCCC
Rhesus     TTAGTGTGCACTGGCAAATCCGGGG-CCGTGTTCTCCCTGCATCGTTTACCCAAAAGGTGACAGTGAACAATCTGCTTCCAAGTCGAGACAGGCTTTCCC
Marmoset   TTAGTGTGCAGTTGCAAATCGGGGG-TCCTGTTCTTCCTGCAACGTTTACCCCAAAGGTAGTAG--GACAGTCTGCTTCCAAGTAGAGACAGGCTTTCCA

Human      ATTGCTATTGAGAAAGGGAATGGAATGGCCTCTGTCC-----------------------------------------------------------GCAT
Chimp      ATTGCTATTGAGAAAGGGAATGGAATGGCCTCTGTCCGCATGTAGACTCTGTCCACATTGATGGCTGACAAGTCACACACTGCCTGAGGTGAGTCTGCAT
Orangutan  ATTGCTATTGAGAAAGGGAATGGAATGGCCTCTGTCCGCATGGTGGTCCT--------TGATGGCTGACGAGGCACACACTGCCTGAGATGAGTCTGCAT
Rhesus     ATTGCTATGGAGAAAGGGAATGGAATGGCCTCTGTCCGTGTGGTGGTCCT--------TCATGGCTGACGATG---AGTCTGCCTGAGATGAGTCTGCGT
Marmoset   GTTGCTCTTGAGAAAGGGAATGGATCAACCTCTGTCCGAGTGGTGGTCCT--------TCATGGCTGATGAGTCACACGCTGCCTGAGGTGAGT--GCAT

Human      GGCTGGAATCC--TCCTCCCCTGCCCCCTGCATGCCTCTCT----GGAGACGCAACTCATTCTGGGAATCCCGCCCGAGTCCTTGCAGGATTAGGGGGCT
Chimp      GGCTGGAATCC--TCCTCCCCTGCCCCCTGCATGCCTCTCT----GGAGACGCAACTCATTCTGGGAATCCCACCCAAGTCCTTGCAGGATTAGGGGGCT
Orangutan  GGCTGGAATCC--TCCTCCTCTGCCCGCTGCAAGCCTCTCTCTCTGGAGATGCAACTCGTTCTGGGAATCCCGCCCGAGTCCTTGCGGGATTAGGGGGCT
Rhesus     GGCTGGAAT-----CCTCTCCCGCCCCCTGCATGCCTCTCT----GGAGACGCAACTCGTTCTGGGAATCCTGCCCAAGTCCTTGCTGGATTAGGGGGCT
Marmoset   GGCTGAGATCCCCTCCTCCCCCACCC-CTCCATGCCTCT------GCAGATGCAGCTCGTTCTGGGAATCCTG----AGTCCTTGCCGGATTAGGGGGCT

Human      TTGCTCCATGAGACCCCACGGGCTTTGA
Chimp      TTGCTCCATGAGACCCCACGGGCTTTGA
Orangutan  TTGCTCCGTGAGACCCCAAGGGCTT-GA
Rhesus     TTGCTCCGTGAGACCCCAAGGGCTTTGA
Marmoset   TTGCTCCGTGAGACCCCAGGGGCTTTGA
```

S1-21: ENSP00000383776 ENST00000400991 ENSG00000215848 H

```
Human       ATGAATGGCGGCCCCCAGAAGAGATGTAAGGTTGAGCACAGGAGCAGGGGTGGTAGAGAGGCCTCAGCACTGGGCTTAGTTCCCAGACTACTTAGAGGAC
Chimp       AGGAATGGCGGCCCCCAGAAGAGATGTAAGGTTGAGCACAGGAGCAGGGGTGGTAGAGAGGCCTCAGCACTGGGCTTAGTTCCCAGACTACTTAGAGGAC
Orangutan   AGGAATGGCGGCCCCCAGAAGAAATGTAAGGTTGAGCACAGGAGCAGGAGTGGTAAAGAGGCCTCAGCACTGGGCTTAGTTCCCAGACTACTTAGAGGAC
Rhesus      AGGAATGGCGGCCCCCAGAAAAGATGTAAGGTTGAGCACAGGAGCAGGGGTGGTAGAGAGGCCTCAGCACGTGGCTTAGTTCCCAGAGGACTTAGAGGAC
Marmoset    A---ATGGCGGCCCGCGGAAGAGACATAAAGTTGAGCACAGGAGCAGGGATG-TAGAGAGGCCTCAGCACCGGGCTTAGTTCCCAGAGGACTTAGAGGAC

Human       TGTGGGTAATAAGAAAAGTACTGGAGGAATCAGAATGGGGGAATATCAGCATGAGGAGAGAGAGAGTAAGATGTGGCCAGGCAGTATCATCTGCTAGTTT
Chimp       TGTGGGTAATAAGAAAAGTACTGGAGGAATCAGAATGGGGGAATATCAGCATGAGGAGAGAGAGAGTAAGATGTGGCCAGGCAGTATCATCTGCTAGTTT
Orangutan   TGTGGGTAATAAGAAAAGTACTGGAGGAATCAGAATGGGGGAATATCAGCATGAGGAGAGAGAGAGTAAGATGTGGCCAGGCAGTATCATCTGCTAGTTT
Rhesus      TGTGAGTAATAAGAAAAGTACTGGAGGAATCAGAATGGGG-AATATCAGCAGGAGGAGTGAGAGGGTAGGATGTGGCCAGGCAGTATCATTTGCCAGTTT
Marmoset    TGTGGGTAGTGTGAAAAGTACCGGAGGAATCAGAATGGGG-AGTATCAGCAGGAGGAGGGAGAGGGTAAGACGTGGGCAGGCAGTATCATCTGCCAGCTT

Human       GAGGGTCTCTCTGGGGCCTGTGAAAGGTGACTGTGTACTTGAGGTGATGCTCCACCTTCTCTCCTCAGCCCAGCACTCTGGT------------------
Chimp       GAGGGTCTCTCTGGGGCCTGAGAAAGGTGACTGTGTACTTGAGGTGATGCTCCACCTTCTCTCCTCAGCCCAGCACTCTGGT------------------
Orangutan   GAGGGTCTCTCTGGGGCCTGTGAAAGGTGACTGTGTACTTGAAGTGATGCTCCACCTTCTCTCCTCAGCCCAGCACTCTGGTTTCCATCTTTGTAACTCT
Rhesus      GAGGGTCTCTCTGGGGCCTGTGAAAGGTGACTGTGTACTTGAGGAAATGCTCCACCTTCTCTCCTCAGCCCAGCACTCTAGTTTCCATCTTTATAACTCT
Marmoset    GAGGGTCTCTCTGGGGCCTGTGAAAGGTGACTGCGTACTTGAGGTGATGCTCCACCTTCTCTCCTCAGCCCAGCACTCTGGTTTCC-----------TCT

Human       ---GAAACTGCATCCTCTCAGAGGCCACTCACAGCCCCCTCAACAAAGACCACTGGCCCTTCCCAAGCCCAGTCCTCCTTGACCTGTATGGAAAATGTTA
Chimp       ---GAAACTGCATCCTCTCAGAGGCCACTCACAGCCCCCTCAACAAAGACCACTGGCCCTTCCCAAGCCCAGTCCTCCTTGACCTGTATGGAAAATGTTA
Orangutan   GGTAAAACTGCACCCGCTCAGAGGCCACTCACAGCCTCCTCAACAAAGACCACTGGCCCTTCCCAAGCCCAGTCCTCCTTGACCTGTATGGAAAATTTTA
Rhesus      GGTAAAACTGCACCCTTTCAGAGGCCACTCATAGCCTCCTCAACAAAGACCTCTGGCCCTTCCCAAGCCCAGTCCTCCTTGACCT--ATGGAAAATTTTA
Marmoset    GGTAAACCTGCACCCTCTCAGAGGCCACTCACAGCCTCCTCAACAAAGACCACTGGCCCTTCCCAAGCCCAGTCCTCCTTGACCTCTGTGGAAAATGTTC

Human       TGCCATTTTCTCAGTCTTTTCCTTTGGCCTTCTGTTCTTCTGTTACATCCATGATCATCTGTTCTTGGTTCCATTCATTCAGTCCTCCGGATGGACCAGT
Chimp       TGCTATTTTCTCAGTCTTTTCCTTTGGCCTTCTGTTCTTCTGTTACATCCATGATCATCTGTTCTTGGTTCCATTCATTCAGTCCTCCGGATGGACCAGT
Orangutan   TGCTATTTTCTCAGTCTTTTCCTTTGGCCTTCTGTTCTTCTGTTACATCCATGATCATCTGTTCTTGGTTCCATTCATTCAGTCCTCCGGATGGATCAGT
Rhesus      TGCTATTTTCTCAGTCTTTTCCTTTGACCTTCGGTTATTCTGTTATATCCGTGATCATCTGTTCTTGGTTCCATTCATTCAGTCCTCCAGATGGACCAGT
Marmoset    CGCTGTTTTCTCAG-------------CCTTTGGTTCTTCTGTTATATCCATGATTGTCTGTTCTTGGTTCCATTCAGTCAGTACTCTGGATGTGCCAGT

Human       TCCTTAA
Chimp       TCCTTAA
Orangutan   TCCTTAA
Rhesus      TCCTTAA
Marmoset    TCCTTAG
```

S1-22: ENSP00000386203 ENST00000408893 ENSG00000221953 H

```
Human    ATGGGCCTGTGCACCCTCCAGCCCCTGGGCCCTCCTCGAAAATCCTCCACATCCTGCGGGACCTGGACCGCCAGCGGCCTCCCAAGCCTGGGCCATTTGC
Chimp    ATGGGCCTGTGCACCCTCCAGCCCCTGGGCCCTCCTGGAAAATCCTTCACATCCTGCGGGACCTGGACCGCCAGCGGCCTCCCAAGCCTGGGCCATTTGC
Gorilla  ATGGGCCTGTGCACCCTCCAGCCCCTGGGCCCTCCTGGAAAATCCTTCACATCCTGCGGNACCTGGACCGCCAGCGGCCTCCCAAGCCTNGGCCATTTGC
Rhesus   GTGGGCCTGTGCACCCTGCAGCCCCTGGGCCCTCCTGGAAAATCCTTCGCATCCTGTGGGACCTGGACCGCCAGCGGCCTCCCAACCCTGGGC-ATCTGC
Baboon   GTGGGCCTGTGCACCCTGCAGCCCCTGGGCCCTCCTGGAAAATCCTTCGCATCCTGCGGGACCTGGACCGCCAGCGGCCTCCCAACCCTGGGC-ATCTGC

Human    CCCGTCGCCTCCGCCTGGCATTCGACTTCTTAGAACCGCGGGCACGAGGGCAGCGTGGGGGCAGTGGTGGCTGTCAGTCCACGCGGGCCGCCGAGAGGAC
Chimp    CCCGTCGCCTCCGCCTGGCATTCGACTTCTTAGAACCCCGGGCACGAGGGCAGCGTGGGGGCAGTGGTGGCTGTCGGTCCATGCGGGCCGCCGAGAGGAC
Gorilla  CCCGTCGCCTCCGCCTGGCATTCGACTTCTTAGAACCCCGGGCACGAGGGCAGCGTGGGGGCAGTGGTGGCTGTCAGTCCATGCGGGCCACCGAGAGGAC
Rhesus   CCC-TCGCCTCCGCCTGGCATTGGACTTCTGAGAACCCTGGGCACGAGGGCAGCGTGGGCGCAGGGGAGGCTGTCAGTCCAGGCCGGC-GCTGAGAGGAC
Baboon   CCC-TCGCCTCCGCCTGGCATCGGACTTCTGAGAACCCTGGGCACGAGGGCAGCGTGCGCGCAGTGGAGGCTGTCAGTCCAGGCCGGC-GCTGAGAGGAC

Human    GCGCCCACCCCACCCTCCAAACGCCGTCCTCCTGCTTCAGCCAGAGCCGTCCCTGACATGGGCCCAGGGACGCGGCTGCAGAGGCCACTTCCGGTCACTC
Chimp    GCGCCCACCCCACCCTCCAAACGCCGCCCTCCTGCTTCAGCCAAAGCCGTCCCAGAGATGGGCCCAGGGACGCGGCTGCAGAGGCCACTTCCGGTCACTC
Gorilla  GCGCCCACCCCACCCTCCAAACGCCGTCCTCCTNCTTCAGCCAAAGCCGTCCCAGAGATGGGCCCAGGGACGCGGCTGCAGAGGCCACTTCCAGTCACTC
Rhesus   GCGTCCACCCCACCCTCCAAACGCCGTCCTGCTGCTTCAGCCAAAGCCGTCCTGGAGACGGGCCCAGGGA---------------------------CTC
Baboon   GCGTCCACCCCACCCTCCAAACGCCGTCCTCCTGCTGCAGCCAAAGCCGTCCCGGAGACGGGCCCAGGGACTCNNNNNNNNNNNNNNNNNNNNNNNNNNN

Human    CCCGCTGCGGCCTCGCGCTCCG----------------------------------------GGAGCCGCACCCTCCGCTGCGCCAGCTC----CGACCG
Chimp    CCCGCTGCGGCCTCGCGCTCCGGGGT---------------------GGCCCCGCGGCTCCAGGAGCCGCACCCTCCGCTGCGCCAGCTC----CGACCG
Gorilla  CCCGCTGCGGCCTCGCGCTCCGGGGT---------------------GGCCCCGCGGCTCCAGGAGCCACACCCCCCGCCGCGCCAGCTC----CGACCG
Rhesus   CCCGCTGCAGCCTCGAGCTCCGGGGTGGCCCCGCGGCTCCCAGGGGTGGCCCCGCGGCTCCAGGAGTCACACACTCCGCCGCGCCAGCTCGGACCGACCG
Baboon   NNNNNNNNNNNNNNNNNNNNNNNNNNNNNNNNNNNNNNNNNNNNNNNNNNNNNNNNGCTCCAGGAGTCACACCCTCCGCCGCGCCAGCTCGGACCGACCG

Human    CAGTCTTCGGGAGCAGAAGCAGCGCCGCGCGGGCCCTGACCCCACACCCT---CCCCGGCGCCTCCGCCTGCAGGACCCCGGCCCTCCCCGGGCTCGCTG
Chimp    CAGCCTTCGGGAGCAGAAGCAGCGCCGCGCGGGCCCCGACCCCACACCCTC-CCCCCGGCGCCTCCGCCTGCAGGACCCCGGCCCTCCCCGGGCTCGCTG
Gorilla  CAGTCTTCGGGAGCAGAAGCAGCGCCGCGCGGGCCCTGACNCCACACCCTCGCCCCCAGCGCCTCCGCCTGCAGGACCCCGGCCCTCCCCGGGCTCGCTC
Rhesus   CAGACTTCGCGAGCAGAAGCAGCGCCGCGCGGGCCCTGACCCCACACCCTG--CCCCGGCGCCTCCGCCTGCAGGACCC------TCCCCGGGCTCGCTG
Baboon   CAGACTTCGGGAGCAGAAGCAGCGCCGCGCGGGCCCTGACCCCACACCCTG--CCCCGGCGCCTCCGCCTGCAGGACCC------TCCCCGGGCTCGCCG

Human    GGTCCCTCCGCCCCGGCCGCTCCCCGCACCGCGCGCGGTGCCTACGAGCTCCAAGGCGGCGCCAGCCAAGACGGTCCCGGCCAAGCGGCGGTGGGTGCGA
Chimp    GGTCCCTCCTCCCCGGCCGCTCCCCGCACCACGCGCGGTGCCTACGAGCTCCAAGGCGGCGCCAGCCAAGACGGTCCCGGCCAAGCGGCGGTGGGTGCGA
Gorilla  GGTCCTTCCTCCCCGGCCGCTCCCCGCACCACGCGCGGTGCCTACGAGCTCCAAGGCGGCGCCAGCCAAGACGGTCCCGGCCAAGCAGCGGTGGGTGCGA
Rhesus   GGTCCTTCCTCCCCCGCCGCTCCCCCGACCGCGCGCGGTGCCGACG-GCTCCAAGGCGGCGCCAGCCAAGACGGTCCCGGCCACGCAGCGGTGGGTGCGG
Baboon   GGTCCTTCCTCCCCCGCCGCTCCCCCGACCGCGCGCGGTGCCGACG-GCTCCAAGGCGGCGCCAGCCAAGACGGTCCCGGCCACGCAGCGGTGGGTGCGG

Human    C------------------------------------CCCCACCACGGGCCCCGGCACCGGCGGGGAGGGCGCGCTCCTAGGCTGCGGGAGCGGACGGAC
Chimp    C------------------------------------CCCCACCACGGGCCCCGGCACCGGCGGGGAGGGCGCGCTCCTAGGCTGCGGGAGCGGACGGAC
Gorilla  C------------------------------------CCCCACCACGGGCCCCGGCACCGGCGGGGAGGGCGCGCTCCTGGGCTGCGGGAGCGGACGGAC
Rhesus   CGGGTGCGGCGGGTGCGGCGGGTGCGGCGGGTGCGGCCCGCACCACGGGCCC-GGCA--GGCGGGGAGGACGCGCTCCCAGTCTCCGGCAGCAGACAGAC
Baboon   C------------------------------------CCCCACCACGGGCCC-GGCA--GGCGGGGCGGACGCGCTCCCAGTCTCCGGCAGCGGACGGAC

Human    CCCTCCGACTTCGGCCAC----------------------CTGGCGCC-GCCGCCTCCTCCCCGCCGAAGTCCCTCCCGGGGCAGCCGCCGCAAACTTTC
Chimp    CCCTCCGACTCCGGCCGC----------------------CTGGCGCC-GCCGCCTCCTCCCCGCCGAAGTCCCTCCCGGGGCAGCCGCCGCCAACTTTC
Gorilla  CCCGCCGACTCCGGCCAC----------------------CTGGCGCC-GCCGCCTCCTCCCCGCCGAAGTCCCTCCCGGNGCAGCCGCCGCAAACTTTC
Rhesus   CCCTCCGACTCCGACTCCGACTCCGACTCCGACTCCGACTCCGACTCCGGCCGCCTCCTCCCCGCCGAGGTCCCTCCGGGGGCGGCCGCCGCACACTTTC
Baboon   CCCTCCGACTCCGAC-----------------------------TCCG-GCCGCCTCCTCCCCGCCGAGGTCCCTCC-GGGGCGGCCGCCGCACACTTTC

Human    CGGAGCGGGAGCGACTGTGA
Chimp    CGGAGCGGGAGCGACTGTGA
Gorilla  CGGAGCGGGAGCGACTGTGA
Rhesus   CGGAGCGGGAGCGACTGTGA
Baboon   CGGAGCGGGAGCGACTGTGA
```

S1-23: ENSP00000386144 ENST00000408897 ENSG00000221891 H-O

```
Human       ATGGAAGGATGCGCGGTGCGGCGGGGCAGCTGTCCTCTTCTCCCCGGACCCAGCGCCTGGAGAGCCAGCCCTGCAGGGTGGGCTGGGCGAGCCAAACTGC
Chimp       ----------------------------------------------------------------------------------------------------
Orangutan   ATGGAAGGATGCGCGGTGCGGCGGGGCAGCTGTCCTCTTCTCCCCGGACCCAGCGCTTGGAGAGCCAGCCCTGCAGGGTGGGCTGGGCCGGCCAAACTGC
Rhesus      ATGGAAGGATGCGTAGTGCGGCGTGGCAGCTGCCCTCTTCTCCCCGGACCCAGCGCCTGGAGAGGCAGCCCTGCAGGGTGGGCTGGGCGAACTAAACTGC
Baboon      ATGGAAGGATGCGTGGTGCGGCATGGCAGCTGCCCTATTCTTCCCGGACCCAGCGCCTGGAGAGCCAGCCCTGCAGAGTGGGCTGGGCGAACTAAACTGC

Human       GTTCCTGGTGCAGGGCTTCGGGTCTCCCTAACAGACCTTATACGCTGACCGGCGGCCGCCATGGCAGTGTCTCTTTGCTCAGACATCCAGGGACGACCAC
Chimp       ----------------------------------------------------------------------------------------------------
Orangutan   GTTCCTGGTGCAGGGCTTCGGGTCTCCCTAACAGACCTTATACGCTGACCGGCAGCCGCCATGGCAGTGTCTCTTTGCTCAGAGATCCAGGGACGACCAC
Rhesus      GTTCCTGGTGCAGGGCTTCGGGTCTCCCTAACATACCTTAAACGTTGAACAGCCGCCGCCTTGGCAGTGTCTCTTTGCTCAGAGATCCTGAGACGACCAC
Baboon      GTTCATGGTGCAGGGCTTCGGGTCTCCCTAACATACCTTAAACGTTGAACCGCGGCCGCCTTGGCAATGTCTCTTTGCTCAGAGATCCTGAGACGACCAC

Human       ATTCGTCCAACAGCGGTCGCTCCACCAATCCTGGGAGAAGCGAATCGTTTTCTCCGCGTGCCCTGTCAGCCGCTCATGGTGCCCAGAG----AGGAATTT
Chimp       ----------------------------------------------------------------------------------------------------
Orangutan   ACTCGTCCAACAGCGGTCGCTCCACCAATCCGGGGAGAAGCGAATCGTTTTCTCCGCGTGCCCTGTCAGCCGCTTATGGTGCCCAGAG----AGGAATTT
Rhesus      ACTCGTCCAACAGCAGTCGCTCCACCAAGCCTGGGGAAAGCGAATCGTTTTCTCCGCGTGCCCTGTCAGCCGCTCATGCTGCCCAGAGAGGAAGGAATTT
Baboon      ACTCGTCCAACA---GTCGCTCCACCAAGCCTGGGAAAAGCGAATCGTTTTCTCCGCGTGCCCTGTCAGCCGCTCATGCTGCCCAGAG----AGGAATTT

Human       TAGTGGCAGCATTCCGGCTGTCACGCCACCGAAATTGCCAGGCCACTCCAAGTCAGAAGGACCACCAGGAAAAGTCAGGAAGAGAACCACCATCAGGTCC
Chimp       ----------------------------------------------------------------------------------------------------
Orangutan   TAGTGGCAACATTCCGGCTGTCACGCCACTGAAATTGCCAGGCCACTCCAAGTCAGAAGGACCACCAGGAAAAGTCAGGAAGAGAACCACCATCAGGTCC
Rhesus      TAGTGGCAACATTCCGGCTGTCACGGCACTGAAATTGCCAGGCCACTCCAAGTCAGAAGGACCACCAGGAAAAGTCAGGAAGAGAATCACCATCAGGTTG
Baboon      TAGTGGCAACATTCCGGCTGTCACGCCACTAAAATTGCCAGGCCACTCCAAGTCAGAAGGACCACCAGGAAAAGTCAGGAAGAGAATCACCATCAGGTCT

Human       CAGCCTCTTTTTGTGACAAGGACTAGAGGGTTTG---GGTCTGCAGTGGGCTGGC-TCCCGCTGGGCTCACCTGTCCTATAG
Chimp       ----------------------------------------------------------------------------------
Orangutan   CAGCCTCTTTTTGTGACAAGGACTAAAGGGTTTG---GGTCTGCAGTGGGCTGGC-TCCCGCTGGGCCCACCTGTCCTATAG
Rhesus      CAGCCTCTTTTTGTGACAAGGACTAGATGGTTGGTTTGGTCTGCAGTGGGCTGTCTTCCCGCTGTACTGGCCTCTCCTGTAG
Baboon      CAGCCTCTTTTTGTGACAAGGACTAGATGGTTT----GGTCTGCAGTGAGCTGTCTTCCCGCTGGACTCACTTCTCCTGTAG
```

S1-24: ENSP00000386220 ENST00000408913 ENSG00000221899 H

```
Human      ATGAATACGTACACCCGCAGCGCCTCCTTCACTCCGAAGACTTTCACTTTCCCCCTTGCACAAAACCGAACATCCCACCCCACCACCACCTCCAAGCTAA
Chimp      ATGAATATGTACACCCGCAGCGCCTCCTTCACTCCGAAGAC--TCACTTTCCCCCTTGCACAAAACCGAACATCCCACCCCACCACCACCTCCAAGCTAA
Orangutan  ATGAATAAGTACACCCGCAGCGGCTCCTTCACTCCGAAGACTTTCACTTTCCCCCTTGCACAAAACCGAACATCCCACCCTACCACCACCTCCAAGCTAA
Rhesus     ATGAATATGTATATCCGCAGCGCCTCCTTCACTCCGAAGACTTTCACTTTCCCCCTTGCACAAAACCGAACATCCCACCCCACCCCCACCTCCAAGCTAA
Marmoset   ATGAATAAGTACATCTGCAGCGCCTCCTCTACTCCGAGGACTTTCACTTTCCCCCTGGCACAAAACCGAACATCCCACCC------CACCCCCAAGCTAA

Human      AATACCTGGGCCGCCCCTCCCCACGAAGGGACGCGCTCCAGGCGAATGGGGAGGGCCCGGAAGTCGGGCCTGGGACAGAGGAGCCGGCCCACCGCCTCCC
Chimp      AATACCTGGGCCGCCCCTCCCCACGAAGGGACGCGCTCCAGGCGAATGGGGAGGGCCCGGAAGTCGGGCCTGGGACAGAGGAGCCGGCCCACCGCCTCCC
Orangutan  AATACCTGGGCCGCCCCTCCCCACGAAGGGACGCGCTCCAGGCGAATGGGGAGGGCCCGGAAGTCGGGCCCGGGACAGAGGAGCCGACCCACCGCCTCCC
Rhesus     AATACCTGGGCCACCCCTCCCCACGAAGGGACGCGCTCCAGGCGAATGGGGAGGGCCCGGAAGTCGGGCCCGGGACGGAGGAGCCGGCCCACCGCCTCCC
Marmoset   AATACCTGGGCCGCCCCTCCCCGGGAAGGGACGCGCTCCGGGCGAACGGG-AGGGCCCGGAAGTCGGGCCCAGGACTGAGGAGCCGGCCCACCGCCTCCT

Human      GGTCGGAAGCGGCAGTGCAGGTGAGGCGGCGGCCAGCCTGGGGACTGCGCGGGTAAGGAGCGGGAGGGCGGAATGCACCGCGAAGTTGGCGGGGGACGGC
Chimp      GGTCGGAAGCGGCAGTGCAGGTGAGGCGGCGGCCAGCCTGGGGACTGCGCGGGTAAGGAGCGGGAGGGCGGAATGCACCGCGAAGTTGGCGGGGGACGGC
Orangutan  GGTCGGAAGCGGCAGTGCAGGTGAGGCGGCGGCCAGCCTGGGGACCGCGCGGGTAAGGAGCGGGAGGGCGGACTGCACCGCGAAGTTGGCGGGGGACTGC
Rhesus     GGTCGGAAGCGGCAGTGCAGGTGAGGCCGCGGCCAGCCTCGGGACTGCGCGGGTAAGGAGCGGGAGGGCCGACTGCACCGCGAAGTTGGCGGGGGACGGC
Marmoset   GGTCGGAGGCCGCAGTGCAGGTGAGGCGGCGGCCACCCTGGGCACGGCG-GGGTAAGGAGCCG-AGGGCGGACTGCACCGGGAAGTTGGTGGGGGTCCGC

Human      CACGCACGCCTCGCCTCGGCCTTCCTGCACCGTAG-----GGCCGCCCTCCCGCCACCCCTGGGCACCTGTCGACCACGTCTGGGCGAGGGCGGGTCGGC
Chimp      CACGCACGCCTCGCCTCGGCCTTCCTGCACCGTAGGGGCTGGCCGCCCTCCCGCCACCCCTGGGCACCTGTCGACCACGTCTGGGCGAGGGCGGGTCGGC
Orangutan  CACGCACGCCTCGCCTCGGCCTTCCTGCACCATAGGGGCTGGCCGTCCTCCCGCCACCCCTGGGCACCTGTTGACCACGTCTGGGCGAGGGCGGGTCGGC
Rhesus     CGCGCACGCCTCGTCTCGGACTTCCTGCCCCGTAGGGGCTGGCCGCCCTCCCGCCACCCCAGGGCACCTGCCGACCACGTCTGGGCGGGGGCGGGCCGGC
Marmoset   CGCGC-CGCCTCGCCTCGGCCTTCCTGCCCCGTCCGGCCTTGCCGCCC------------GGGACACCAGCGGAGCACGTCTGGGCGGGGGCGGGCCGGC

Human      TCCCGCCCTCGGGCCCTCACC---------------GCGGCGGGGACGCCGGGTCAGCGAAGGGTTAAGGGACTCGCTCCCTCCTCTCGGCTCCCTCACC
Chimp      TCCCGCCCTCGGGCCCTCACC---------------GCGGCGGGGACGCCGGGTCAGCGAAGGGTTAAGGGACTCGCTCCCTCCTCTCGGCTCCCTCACC
Orangutan  TCCCGCCCTCGGGCCCTCACCCCACCCCGGCCAGCCGCGGCGGGGACGCCGGGTCAGCGAAGGGTTAAGGGACTCGCTCCTTCCTCTCGGCTCCCTCACC
Rhesus     TCCCGCCCTCGGGCCCTCACCCCACCCCGGCCAGCCGCGGCGGGGACGTCGGGTCAGCGAAGGGTTAAGGGACTTGCTCCCTCCTCTCGGCTCCCTCACC
Marmoset   TCACGCCCTCGGACCCTCACCCCATCCCGGCCAGCCGCAGCGGCGACACCGGGTCAGCGAAGGGTTAAGGAACTCGCTCCCTCCCCTCGGTTCCCTCACC

Human      CTTGGAAAGTCCCCGAAATGA
Chimp      CTTGGAAAGTCCCCGAAATGA
Orangutan  CTTGGAAAGTCCCCGAAATGA
Rhesus     CTTGGAAAGTCCCCGAAATGA
Marmoset   CTTGGAAAGTCCCCGAAATGA
```
