# Supplementary figures and images for: Hominoid-Specific De Novo Protein-Coding Genes Originating from Long Non-Coding RNAs
Source: PLoS Genet. 2012 Sep 13;8(9):e1002942. doi: 10.1371/journal.pgen.1002942 (PMC3441637; doi:10.1371/journal.pgen.1002942)

Figure S1

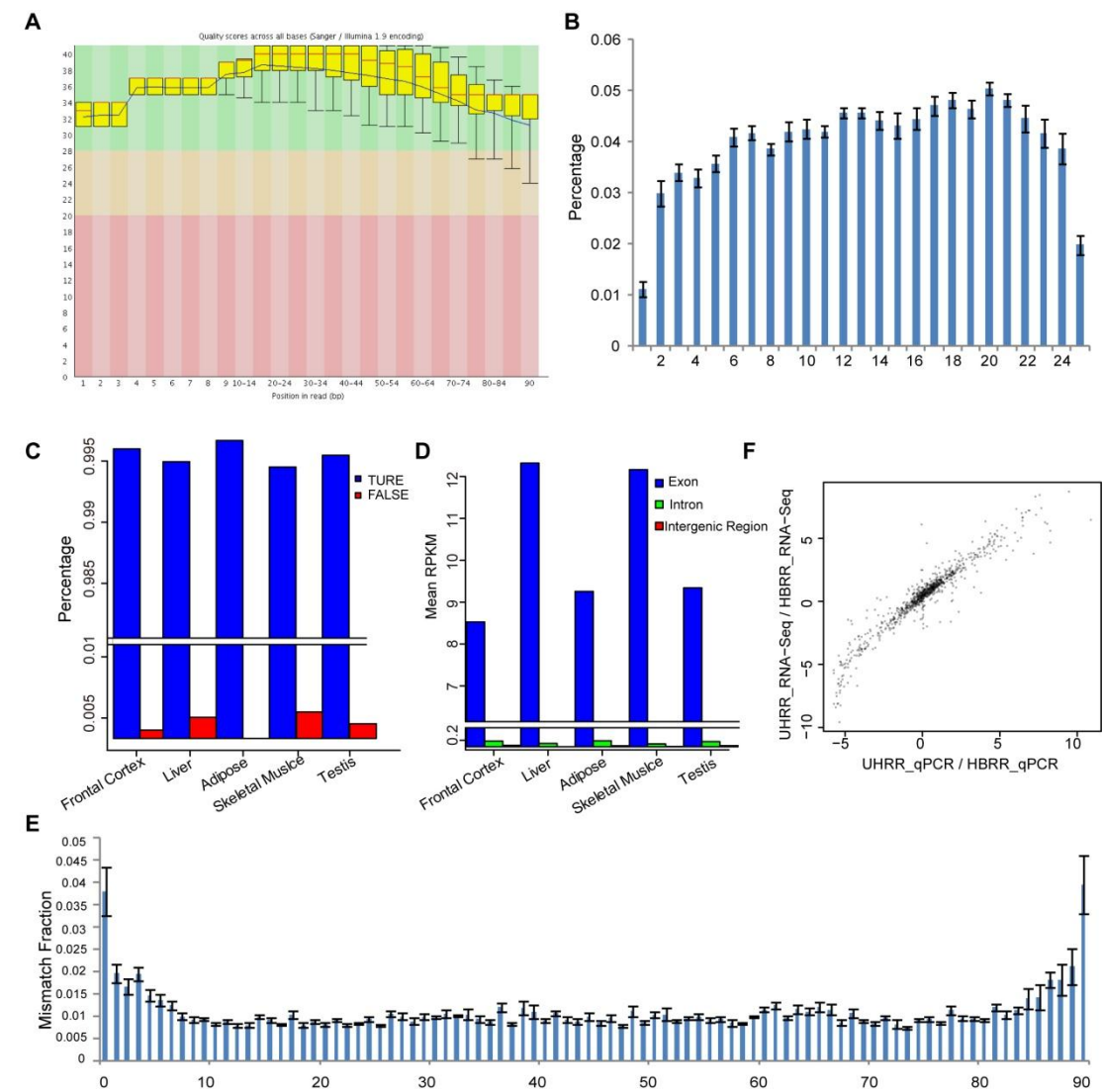

Supplement: Figure S1 — Strand-specific mRNA-Seq analysis in five rhesus tissues reveals comprehensive transcriptome information for rhesus macaque. (A) For each position of the reads, PHRED quality scores across all reads were calculated and summarized. The central red line is the median value, the yellow box represents the inter-quartile range (25–75%), the upper and lower lines represent the 10% and 90% points, and the blue line represents the mean quality. (B) Distribution of short reads on transcripts. The even distribution reveals well-controlled randomized fragmentation of the transcripts in the RNA-Seq experiments. Data are shown as mean ± SD. (C) Evaluation of strand-specific strategy. The reads with correct strand information were >100-fold more than strand-mislabeled reads for all five tissues from rhesus macaque. (D) RPKM scores of exonic regions, intronic regions and intergenic regions, in five rhesus tissues. (E) For each position of the reads, mutation rates were calculated and summarized. The average mutation rate was 1.51 errors per read. Data are shown as mean ± S.D. (F) Scatter plot showing relative expression of 877 transcripts between reference brain (HBRR) and UHR sample (UHRR) as measured by Taqman Gene Expression Assays (UHRR_qPCR/HBRR_qPCR) and by strand-specific RNA-Seq read counts (UHRR_RNA-Seq/HBRR_RNA-Seq). The Spearman correlation coefficient was calculated and is shown with the linear regression curve (R = 0.945). (PDF) [file pgen.1002942.s002.pdf]

**Figure S2**

**S2-1: Human**

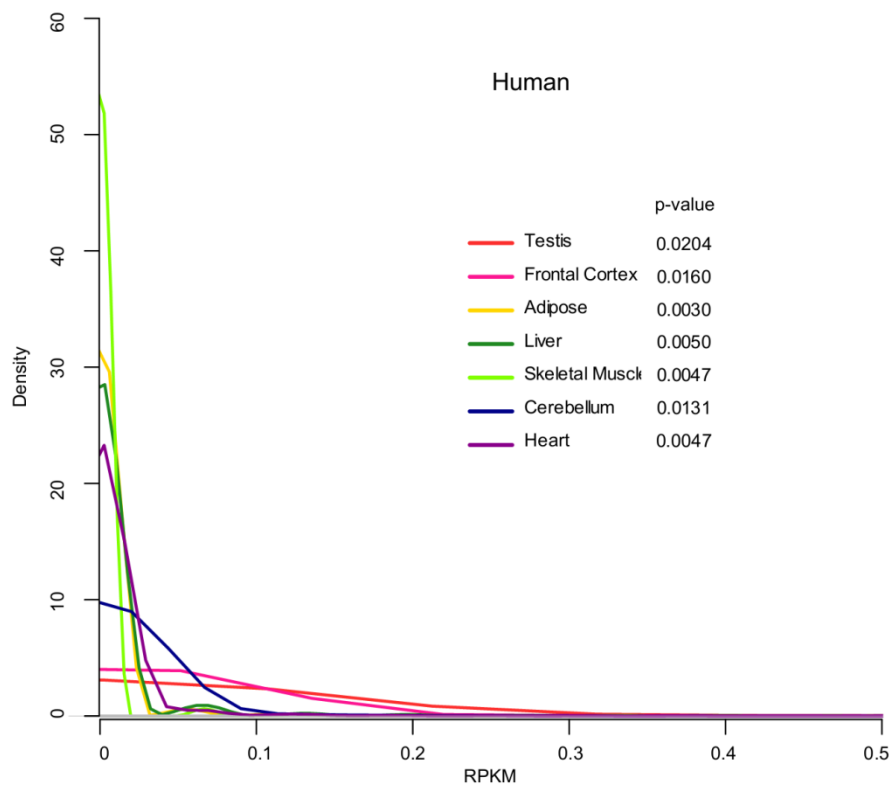

**S2-2: Chimpanzee**

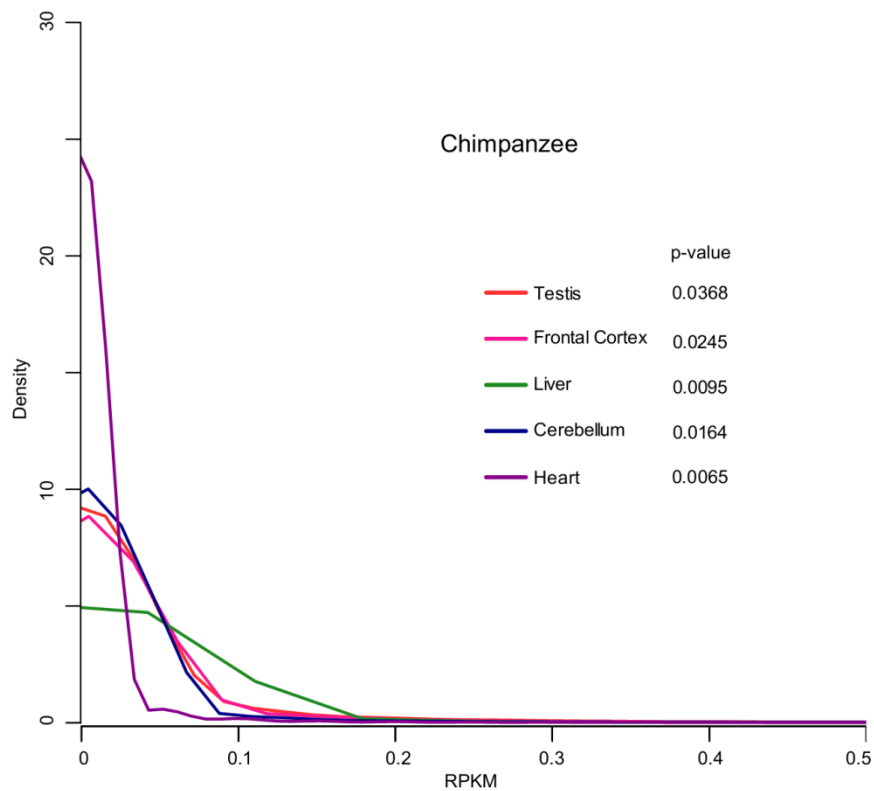

S2-3: Rhesus Macaque

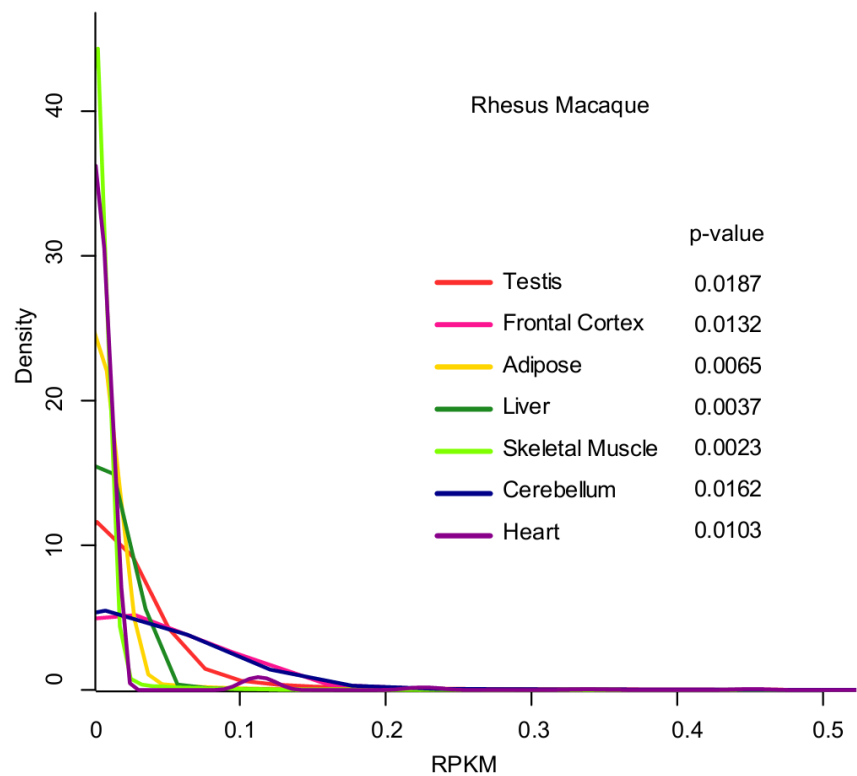

Supplement: Figure S2 — Estimation of RPKM scores for the genomic background represented by intergenic regions. 10,000 intergenic regions were randomly selected and used to calculate RPKM scores from seven human tissues (adipose, skeletal muscle, prefrontal cortex, cerebellum, heart, liver, testis; S2-1), five chimpanzee tissues (prefrontal cortex, cerebellum, heart, liver, testis; S2-2) and seven rhesus macaque tissues (adipose, skeletal muscle, prefrontal cortex, cerebellum, heart, liver, testis; S2-3). For each tissue type, the distribution of RPKM scores is illustrated and the number of regions with a score >0.2 were counted, which was further used to estimate p-values for the genomic background transcription with an RPKM cutoff of 0.2. (PDF) [file pgen.1002942.s003.pdf]

**Figure S3**

S3-1: Chimpanzee

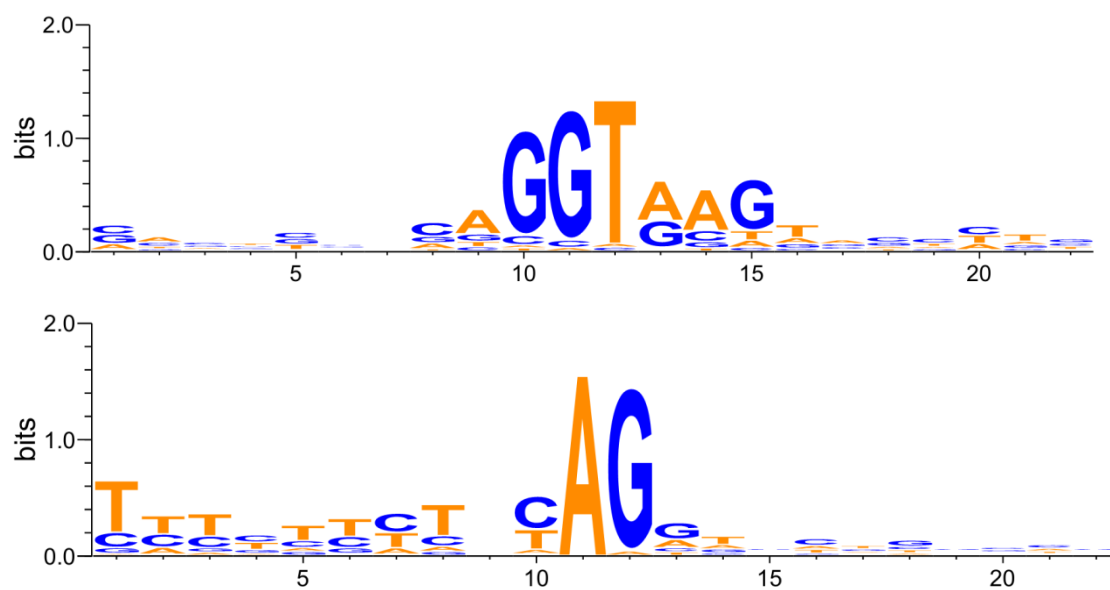

S3-2: Rhesus Macaque

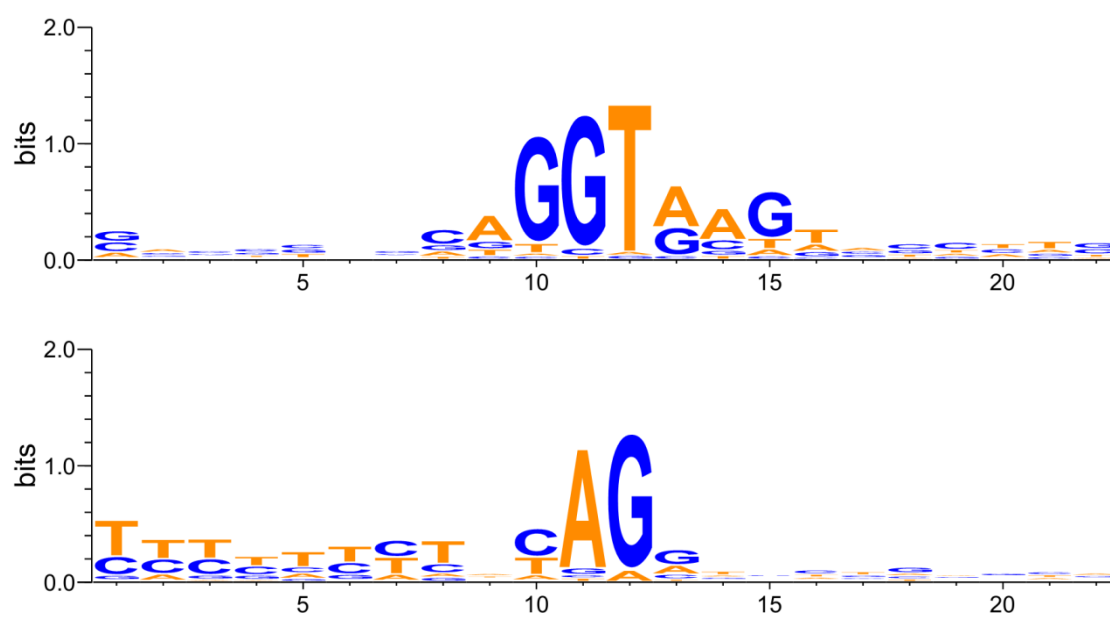

Supplement: Figure S3 — Sequence motif flanking the splicing junctions in chimpanzee and rhesus macaque. Sequence motifs near both the donor site and acceptor site, summarized by splicing junctions in de novo genes in chimpanzee (S3-1) and rhesus macaque (S3-2). (PDF) [file pgen.1002942.s004.pdf]

**Figure S4**

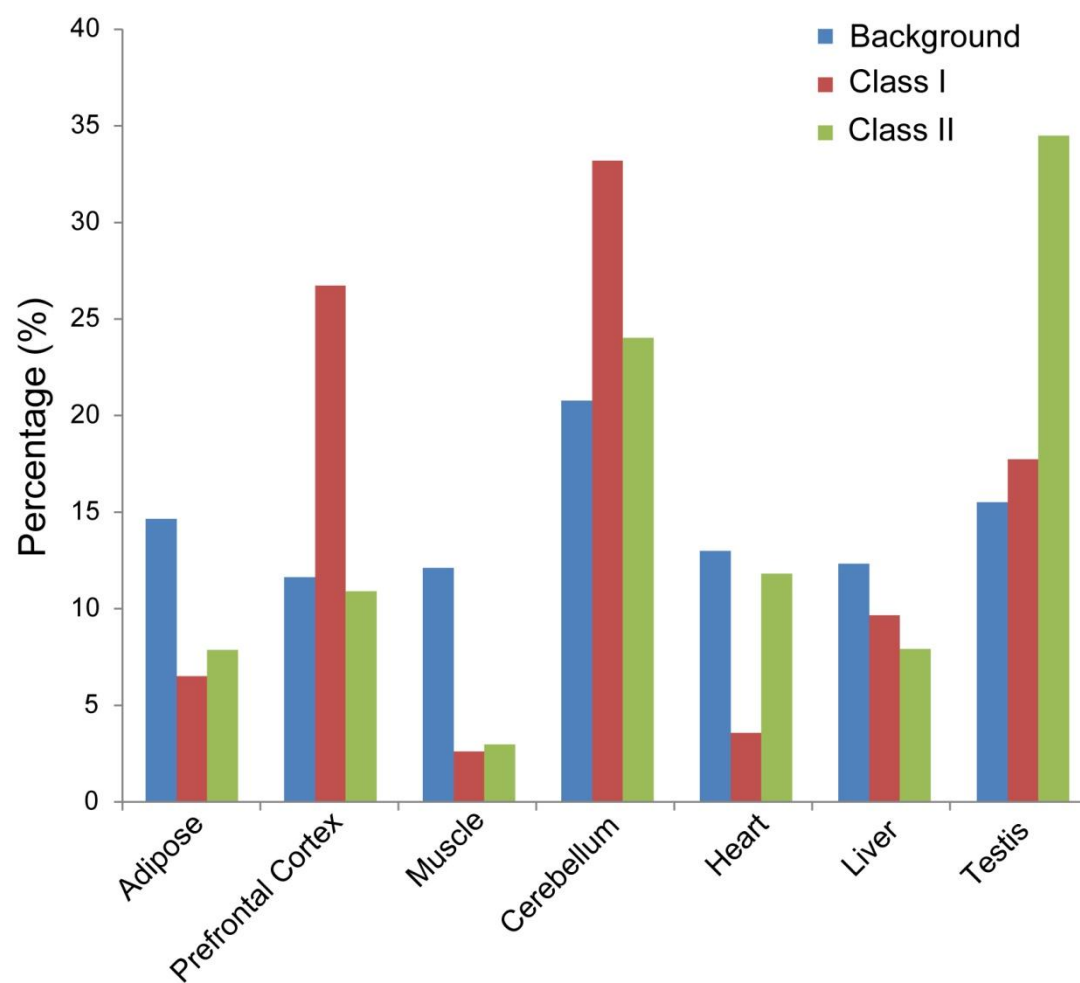

Supplement: Figure S4 — Tissue-enriched expression of de novo genes in human. RNA-Seq read distribution for class I and class II de novo genes, using the proportion of the RNA-Seq library of seven tissues as background. (PDF) [file pgen.1002942.s005.pdf]

Figure S5

S5-1: Class I

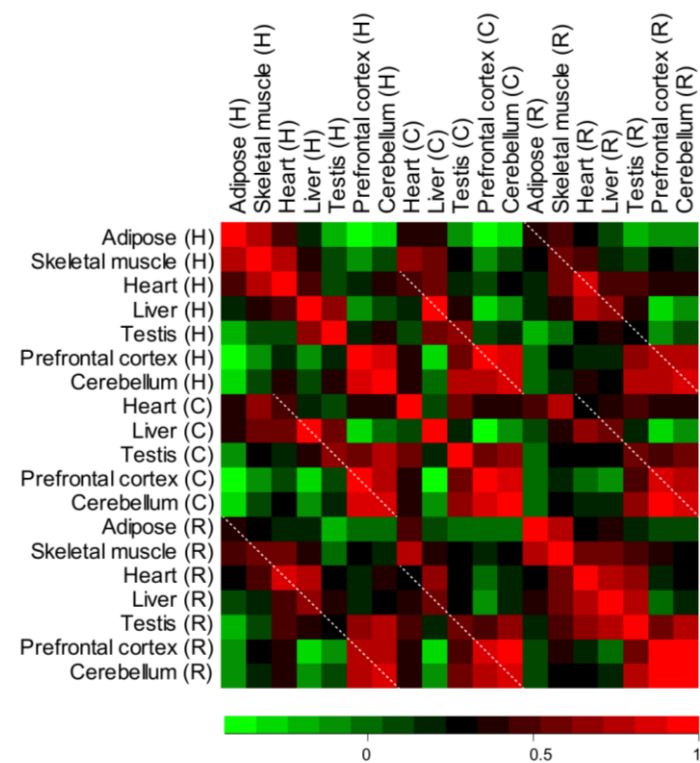

S5-2: Class II

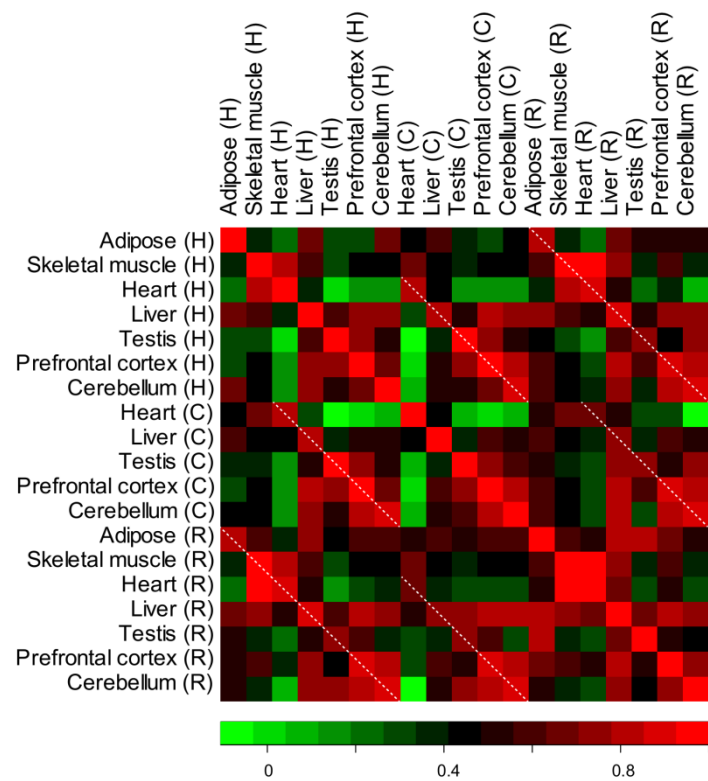

Supplement: Figure S5 — Non-coding orthologs of human de novo protein-coding genes show tissue expression profiles similar to human. For each pair of tissues, Spearman correlation coefficients were computed separately and the extent of tissue-specific differences in de novo gene expression are shown for Class I (S5-1) and Class II genes (S5-2). Comparisons between pairs of corresponding tissues in different species are highlighted with dotted lines. (PDF) [file pgen.1002942.s006.pdf]

Figure S6

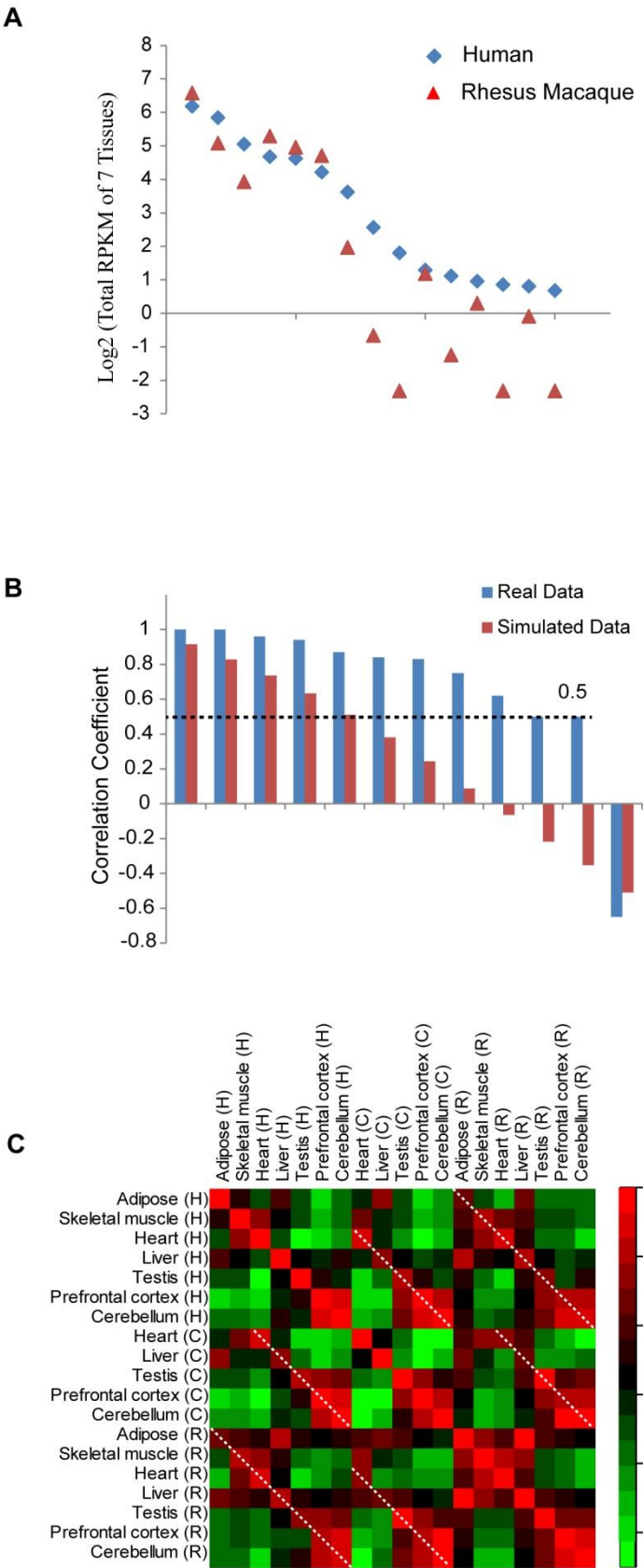

Supplement: Figure S6 — De novo protein-coding genes with stricter out-group ORF length cutoff encoded non-coding RNAs in the rhesus macaque with a correlated tissue expression profile and lower expression level. (A) Summed RPKM scores (log2 transformed) of de novo genes in seven tissues from human and rhesus macaque. The human genes are ordered with decreasing expression levels as reference, and genes in rhesus macaque are aligned accordingly. (B) Correlation coefficients for tissue expression profiles between human and rhesus macaque. The real data for de novo genes (brown histograms) are illustrated with background simulated by 10,000 Monte Carlo simulations neglecting ortholog relationship for the tissue expression profile. (C) For each pair of tissues, Spearman correlation coefficients were computed separately and the extent of tissue-specific differences in de novo gene expressions are shown. (PDF) [file pgen.1002942.s007.pdf]

Figure S7

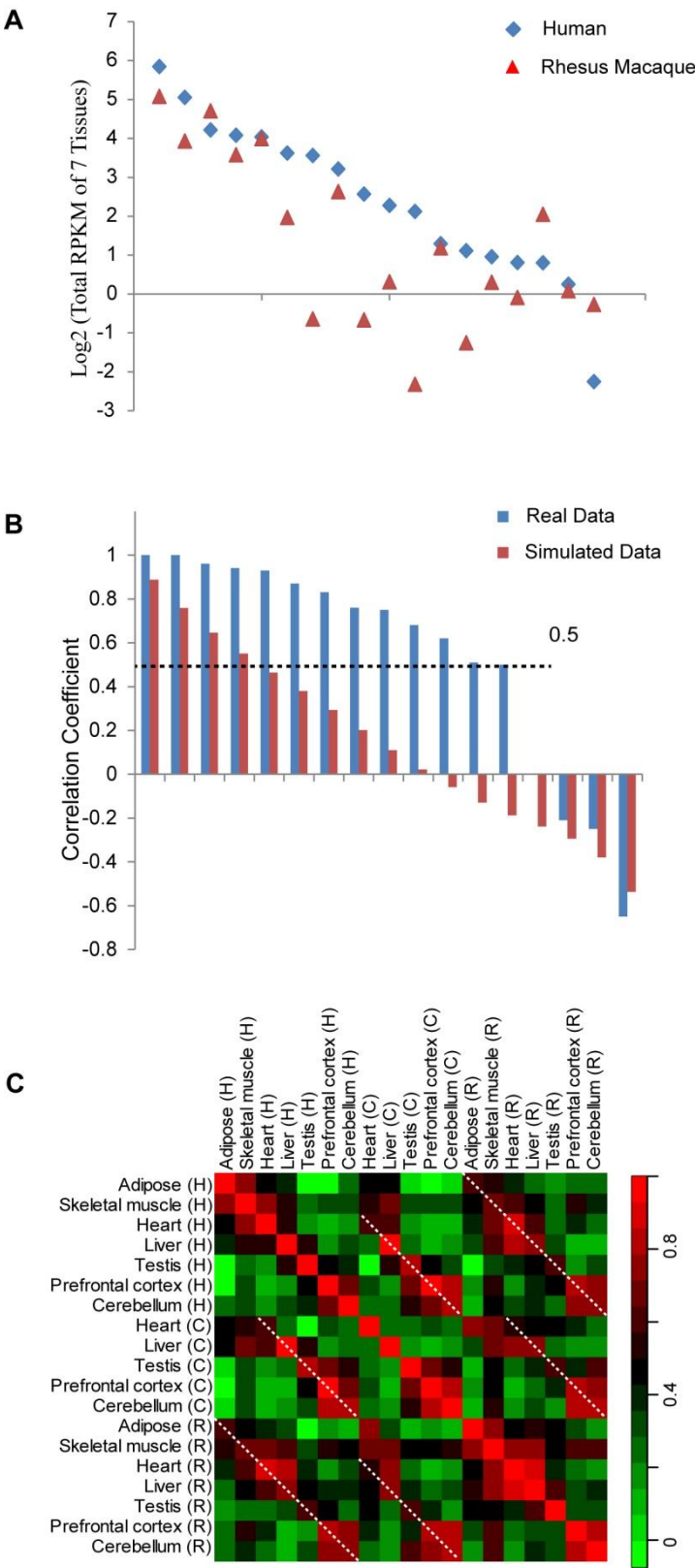

Supplement: Figure S7 — De novo protein-coding genes with single coding exon encoded non-coding RNAs in the rhesus macaque with a correlated tissue expression profile and lower expression level. (A) Summed RPKM scores (log2 transformed) of de novo genes in seven tissues from human and rhesus macaque. The human genes are ordered with decreasing expression levels as a reference, and the rhesus genes are aligned accordingly. (B) Correlation coefficients for tissue expression profiles between human and rhesus macaque. The real data for de novo genes (brown histograms) are illustrated with background simulated by 10,000 Monte Carlo simulations neglecting ortholog relationship for the tissue expression profile. (C) For each pair of tissues, Spearman correlation coefficients were computed separately and the extent of tissue-specific differences in de novo gene expression are shown. (PDF) [file pgen.1002942.s008.pdf]

Figure S8

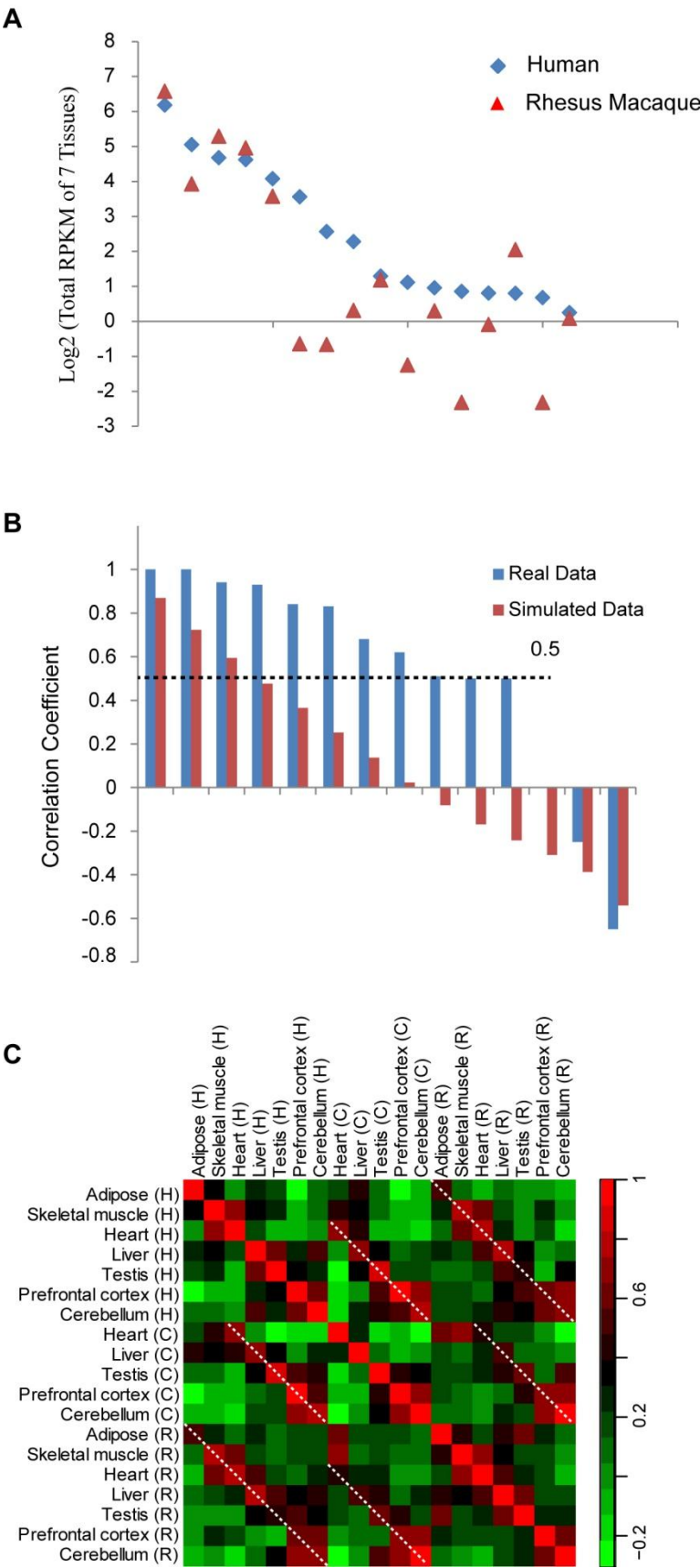

Supplement: Figure S8 — De novo protein-coding genes without Alu-element encoded non-coding RNAs in rhesus macaque with a correlated tissue expression profile and lower expression level. (A) Summed RPKM scores (log2 transformed) of de novo genes in seven tissues from human and rhesus macaque. The human genes are ordered with decreasing expression levels as a reference, and the rhesus genes are aligned accordingly. (B) Correlation coefficients for tissue expression profiles between human and rhesus macaque. The real data for de novo genes (brown histograms) are illustrated with background simulated by 10,000 Monte Carlo simulations neglecting ortholog relationship for the tissue expression profile. (C) For each pair of tissues, Spearman correlation coefficients were computed separately and the extent of tissue-specific differences in de novo gene expression are shown. (PDF) [file pgen.1002942.s009.pdf]

Figure S9

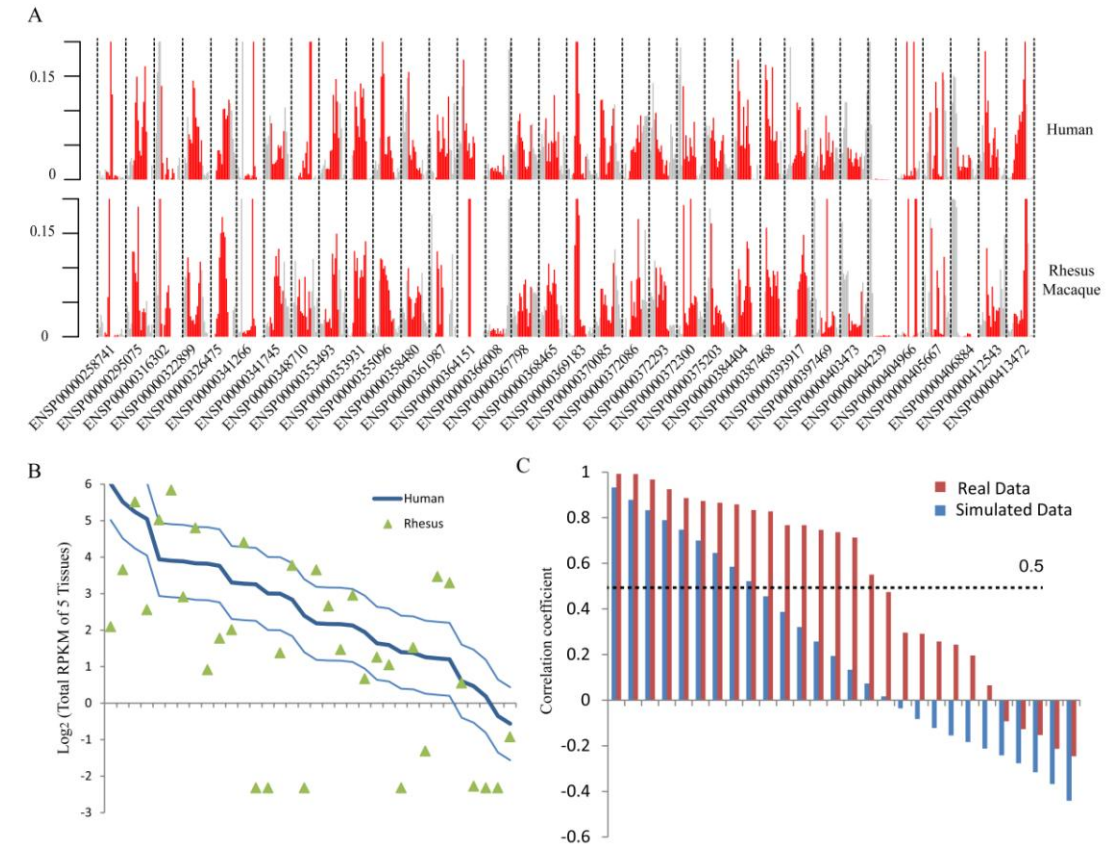

Supplement: Figure S9 — Human-specific de novo protein-coding genes (identified by Wu et al) encoded non-coding RNAs in rhesus macaque with fixed transcript structure and correlated tissue expression profile. (A) For each de novo gene, the base-level density of RNA-Seq reads across the transcript (red), as well as the upstream/downstream regions (grey, 50% of the length of the transcript), are shown. The raw density scores computed from RNA-Seq read coverage were normalized to the total reads across the region. (B) Summed RPKM scores (log2 transformed) of 34 de novo genes in five tissues from human and rhesus macaque. The human genes are ordered and connected with decreasing expression levels as a reference, and the rhesus macaque genes are aligned accordingly. The thinner lines indicate a two-fold change of expression, compared with the reference expression level. (C) Correlation coefficients for tissue expression profiles between human and rhesus macaque. The real data for the 28 de novo genes (brown histograms) are illustrated with background simulated by 10,000 Monte Carlo simulations neglecting ortholog relationship for the tissue expression profile (blue histograms). (PDF) [file pgen.1002942.s010.pdf]

Figure S10

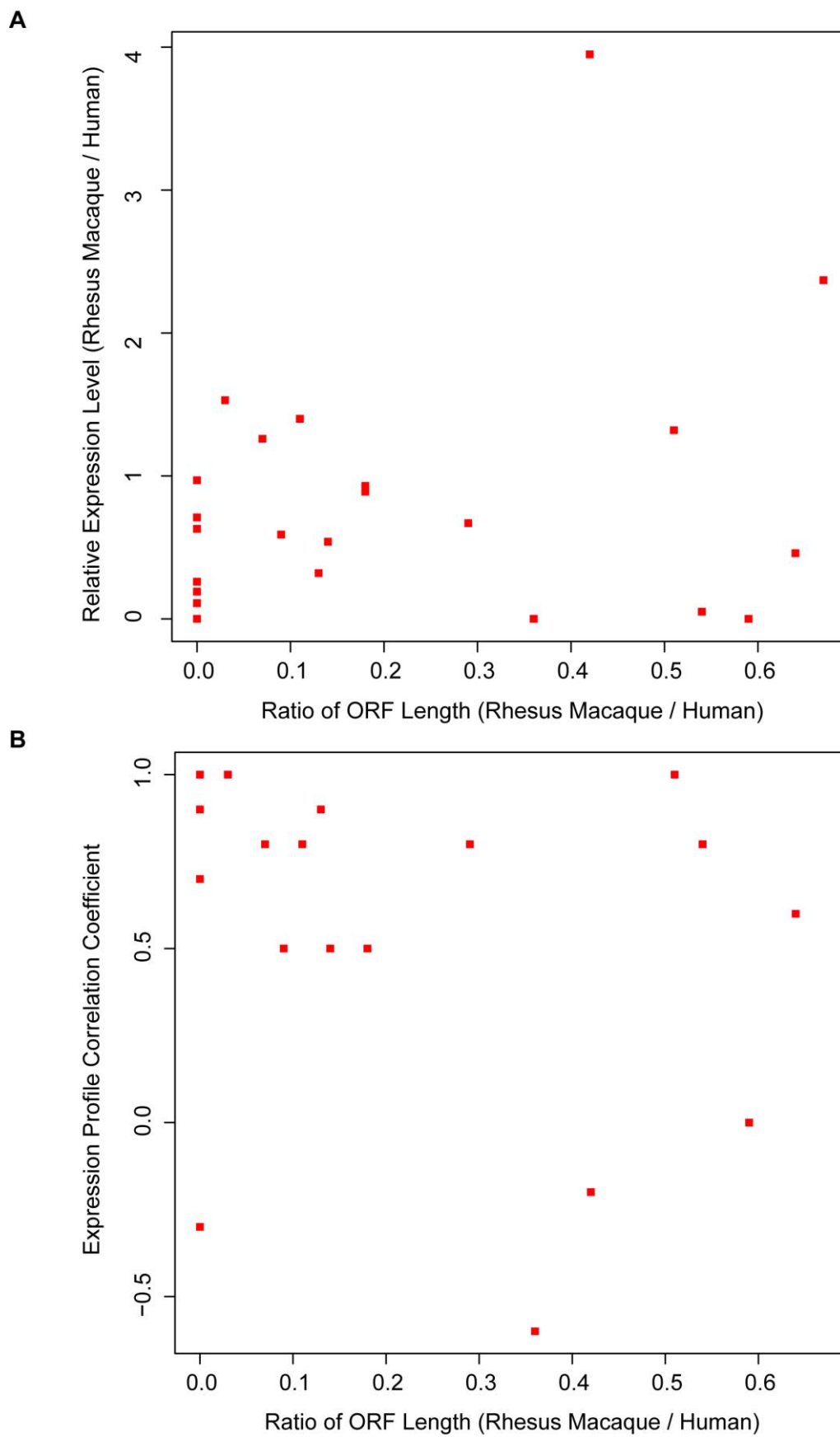

Supplement: Figure S10 — ORF expansion size has no correlation with ancestral transcription activity and tissue expression profile. For each de novo gene, the “Ratio of ORF Length” was calculated by normalizing the size of the truncated ORF in rhesus macaque to the size of the intact ORF in human. The “Relative Expression Level” was further determined as the ratio of summed expression in seven macaque tissues to that of corresponding human tissues. The Spearman correlation coefficient between human and rhesus macaque tissue expression profiles were calculated as before. The correlations between the “Ratio of ORF Length” and “Relative Expression Level” (A), as well as “Expression Profile Correlation Coefficient” (B) are shown in scatter diagrams. (PDF) [file pgen.1002942.s011.pdf]
