# Supplementary material for: Hominoid-Specific De Novo Protein-Coding Genes Originating from Long Non-Coding RNAs
Source: PLoS Genet. 2012 Sep 13;8(9):e1002942. doi: 10.1371/journal.pgen.1002942 (PMC3441637; doi:10.1371/journal.pgen.1002942)
Supplement: Table S1 — The phylogenetic distribution of ORF “disabling” mutations in multiple primate out-groups for de novo genes. (PDF) [file pgen.1002942.s012.pdf]

Table S1: The phylogenetic distribution of ORF "disabling" mutations in multiple primate out-groups for *de novo* genes

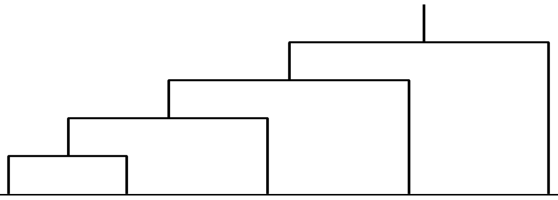

Ensembl ID	Status[#]	Human	Chimp	Orangutan	Rhesus	Marmoset
ENST00000273641	H	+	-	-	-	-
ENST00000308946	H	+	-	-	-	-
ENST00000315302	H-C	+	+	-	-	-
ENST00000318659	H-C	+	+	-	-	-
ENST00000324987	H-C	+	+	-	-	-
ENST00000326341[@]	H	+	-	-	-	-
ENST00000327903	H-C	+	+	-	-	-
ENST00000370523	H-C-O	+	+	+	-	-
ENST00000370535	H	+	-	-	-	-
ENST00000373170	H-C-O	+	+	+	-	-
ENST00000376812[@]	H	+	-	-	-	-
ENST00000377006	H-C	+	+	-	-	-
ENST00000377064	H-C	+	+	-	-	-
ENST00000391430	H-C-O	+	+	?	-	-
ENST00000391812	H-C-O	+	+	+	-	-
ENST00000397571	H-C-O	+	+	?	-	-
ENST00000397608	H-C	+	+	-	-	-
ENST00000399070	H	+	-	-	-	-
ENST00000400385	H	+	-	-	-	-
ENST00000400449	H	+	-	-	-	-
ENST00000400991	H	+	-	-	-	-
ENST00000408893	H	+	-	-[*]	-	-[**]
ENST00000408897	H-C-O	+	?	+	-	-[**]
ENST00000408913	H	+	-	-	-	-

[#]H: human-specific *de novo* genes (Class I), H-C: *de novo* genes shared by human and chimpanzee (Class II); H-C-O: *de novo* genes shared by human, chimpanzee and or orangutan (Class II); all of these genes were absent in rhesus macaque.

[*]Gorilla sequence was used in the alignment.

[**]Baboon sequence was used in the alignment.

[@]Genes reported in previous study as human-specific *de novo* protein-coding genes.
