# Supplementary material for: Hominoid-Specific De Novo Protein-Coding Genes Originating from Long Non-Coding RNAs
Source: PLoS Genet. 2012 Sep 13;8(9):e1002942. doi: 10.1371/journal.pgen.1002942 (PMC3441637; doi:10.1371/journal.pgen.1002942)
Supplement: Table S2 — Basic information for de novo genes. (PDF) [file pgen.1002942.s013.pdf]

Table S2: Basic information for *de novo* genes

Ensembl ID[#]			Protein length	Exon	Overlapped gene	Bi-promoter
ENSP00000273641	ENST00000273641	ENSG00000145063	174	7	No	FLJ33534:C2orf50
ENSP00000308330	ENST00000308946	ENSG00000172927	313	3	OCIM	No
ENSP00000323252	ENST00000315302	ENSG00000177822	148	4	ODZ3	MGC45800:ODZ3
ENSP00000322693	ENST00000318659	ENSG00000179522	230	3	ENSG00000196542	No
ENSP00000322161	ENST00000324987	ENSG00000215071	121	2	ENSG00000137266<br>ENSG00000180822	CR593815:AK096543
ENSP00000325255[@]	ENST00000326341[@]	ENSG00000178803[@]	159	3	ENSG00000128271	No
ENSP00000330965	ENST00000327903	ENSG00000182457	135	3	No	No
ENSP00000359554	ENST00000370523	ENSG00000174407	99	2	ENSG00000174403<br>ENSG00000199017	No
ENSP00000359566	ENST00000370535	ENSG00000203930	103	3	No	No
ENSP00000362265	ENST00000373170	ENSG00000204091	100	2	No	No
ENSP00000366008[@*]	ENST00000376812[@*]	ENSG00000204626[@*]	163	2	ENSG00000197653	No
ENSP00000366205	ENST00000377006	ENSG00000204666	122	3	ENSG00000213861	No
ENSP00000366264	ENST00000377064	ENSG00000204674	123	1	ENSG00000197136	AK126599:AK308625
ENSP00000375249	ENST00000391430	ENSG00000212736	115	1	ENSG00000154122	No
ENSP00000375688	ENST00000391812	ENSG00000167747	117	5	ENSG00000221381<br>ENSG00000221241<br>ENSG00000220988	No
ENSP00000380701	ENST00000397571	ENSG00000214112	72	3	ENSG00000173918	No
ENSP00000380733	ENST00000397608	ENSG00000214130	149	3	ENSG00000181072	No
ENSP00000382022	ENST00000399070	ENSG00000118267	423	3	No	AY234408:ZNF271
ENSP00000383235	ENST00000400385	ENSG00000215458	302	2	No	No
ENSP00000383299	ENST00000400449	ENSG00000215494	152	2	ENST00000157617	No
ENSP00000383776	ENST00000400991	ENSG00000215848	161	4	ENSG00000125462<br>ENSG00000207933	No
ENSP00000386203	ENST00000408893	ENSG00000221953	237	1	No	No
ENSP00000386144	ENST00000408897	ENSG00000221891	157	1	ENSG00000183439	No
ENSP00000386220	ENST00000408913	ENSG00000221899	166	1	ZFP36L1	No

[@]Genes previously reported by Knowles *et al* [1] as human-specific *de novo* protein-coding genes. [*]Genes previously reported by Wu *et al* [2] as human-specific *de novo* protein-coding genes. [#]Although the initial gene list identified by the genome-wide pipeline covered all three human-specific *de novo* genes (ENSP00000325255, ENSP00000366008 and ENSP00000367746) reported by Knowles *et al* [1], *FLJ33706* (ENSP00000354019) by Li *et al* [3], as well as five human-specific *de novo* genes (ENSP00000300458, ENSP00000358486, ENSP00000366008, ENSP00000369345 and ENSP00000353931) by Wu *et al* [2], some of these genes were filtered out by stringent inclusion criteria as summarized in Materials and Methods.

1. Knowles DG, McLysaght A (2009) Recent *de novo* origin of human protein-coding genes. Genome Res 19: 1752-1759.
2. Wu D-D, Irwin DM, Zhang Y-P (2011) De Novo Origin of Human Protein-Coding Genes. PLoS Genet 7: e1002379.
3. Li CY, Zhang Y, Wang Z, Zhang Y, Cao C, et al. (2010) A human-specific *de novo* protein-coding gene associated with human brain functions. PLoS Comput Biol 6: e1000734.
