# Supplementary material for: Hominoid-Specific De Novo Protein-Coding Genes Originating from Long Non-Coding RNAs
Source: PLoS Genet. 2012 Sep 13;8(9):e1002942. doi: 10.1371/journal.pgen.1002942 (PMC3441637; doi:10.1371/journal.pgen.1002942)
Supplement: Table S3 — Expression evidence for de novo genes. (PDF) [file pgen.1002942.s014.pdf]

Table S3: Expression evidence for *de novo* genes

Ensembl ID	mRNA / spliced EST	RNA-Seq[#]	Peptide	
ENST00000273641	5	brain, 0.82	LWGRHHLK<br>MHYGRKQVSWIIFLK<br>MQILGSTLKLFR	PRIDE: 8653<br>PRIDE: 9327<br>PRIDE: 8653
ENST00000308946	>10	breast, 23.69	ACLTLHRHPTPHCSTWGLPLR<br>CCLCLEQSPSWCHCLR<br>DSLLMFTRQAGHFVEGSK<br>GAQTGAGLSQEAEDVDVSR<br>GVSFLTFHLHQSVPLGDR<br>MALRICVTYTPALPIGLCTR<br>MDVALRSPGR<br>VGVEQAISSCPEEVHGRHGLSME<br>IMWAR<br>VTDAPQGTLCGTGNR	PRIDE: 8659<br>PRIDE: 8653; 8663; 8670<br>PRIDE: 8666<br>PRIDE: 8670<br>PRIDE: 8653; 8658<br>PRIDE: 8671<br>PRIDE: 8658<br>PRIDE: 8658<br>PRIDE: 8662
ENST00000315302	>10	fat, 1.74	ACLGLPSECGSLVRAR<br>PGLPRPSPTCPWK<br>RDPSGSFR	PRIDE: 8668<br>PRIDE: 8653<br>PRIDE: 8668
ENST00000318659	>10	brain, 1.33	EQAPRVSAPPGR<br>FLTNKWGGMLAWPPAAPAR<br>PQSSHLLPPVYPPAPLPPPKDVPL<br>Q<br>RSPAALTPGLLRPLAK	PRIDE: 8665<br>PRIDE: 8665<br>PRIDE: 8665<br>PRIDE: 288; 333
ENST00000324987	2	breast, 0.53	MQICFLALWGGTMAVATSAETAL<br>RTSYASLK<br>SPWGLDNILAPRDVSNYK	PRIDE: 8663<br>PRIDE: 10531; 8657
ENST00000326341[@]	3	liver, 2.36	EQSQKPCSNGGPAAAGEGR<br>IGTGCSHTWDWR<br>MEQDWQPGEEVTPGPEPCSK<br>PCSNGGPAAAGEGR<br>PCSNGGPAAAGEGRVLPSPCFPW<br>STCQAAIHK<br>WQGCTRPALLAPSLATLK	PRIDE: 8663<br>PRIDE: 8661; 8664<br>PRIDE: 8663<br>PRIDE: 69; 73; 74; 75; 76; 8653; 8661: 8664; 8667<br>PRIDE: 8663<br>PRIDE: 8668; 8672
ENST00000327903	>10	testis, 8.20	KETTTEQALPPALLLGIR<br>PEFLSRPSVQTEDAPSK	PRIDE: 8658<br>PRIDE: 8653; 8663; 8669
ENST00000370523	0	breast, 9.17	AGGDASPCSWERLLCYGWSHC<br>EMSITHKEKENAHLKEILLFVNA<br>EAFSQPQPHSAPVCEG<br>KPADAQVSPEMSITHK<br>LLCYGWSHC	PRIDE: 8653<br>PRIDE: 8531<br>PRIDE: 8671<br>PRIDE: 9328; 9342
ENST00000370535	>10	brain, 0.76	FVDMEFLDQMILR	PRIDE: 8662

			MPLEKFVDMEFLDQMILR	PRIDE: 8670
ENST00000373170	>10	testis, 44.27	ISSTPPSGSR	PRIDE: 8653
ENST00000376812[@]	1	brain, 2.53	GTHSCGPRESGGPDTCHLPCH	PRIDE: 8653; 8665
			LERCMVPESEWAPWQPQLPCEPK	PRIDE: 8661; 8664; 8670
			NPHSWGIKAHGLR	PRIDE: 8670
			PHRESGLR	PRIDE: 8653
			RGTHSCGPRESGGPDTCHLPCH	PRIDE: 8653; 8665
			THSCGPR	PRIDE: 8653
ENST00000377006	3	testis, 0.56	APEGDGPLMADAGETLASGGPR	
			VEHTK	PRIDE: 8672
			SWLILASVCGVEKAIHAAASISCG	PRIDE: 8671
			SIAKYVL	PRIDE: 8659; 8667
			VEHTKSWLILASVCGVEK	
ENST00000377064	1	lymph, 3.78	AHAQTTNPYWADTNTR	PRIDE: 9299
			ILSGKGSGGSAR	PRIDE: 8653
			KSQEEAAPPSPRPQSR	PRIDE: 9331
			LRSSSSGNSLLR	PRIDE: 8653; 9333
			PLTTDWRILSGK	PRIDE: 8661; 8664
			RILSGKGSGGSARAVSK	PRIDE: 8409
ENST00000391430	0	lymph, 1.97	MLNWLAQIQQIAKSGR	PRIDE: 8659
ENST00000391812	>10	testis, 22.15	ASCRLGEEPPPLPYCDQAYGEELSIR	PRIDE: 9335
			MTVLEAVLEIQAITGSR	PRIDE: 8666
			TDTAWPGAPGVKQAR	PRIDE: 608
ENST00000397571	>10	heart, 2.48	LGRQIQPPWPLAHAR	PRIDE: 8665
ENST00000397608	3	heart, 2.30	AAAFLHAAQGSSMPGAGMR	PRIDE: 3669; 3674; 8653
			EVSLDSR	PRIDE: 2674
			FCSLLAILASPNERALK	PRIDE: 8660; 8663; 8669
			LAPPRR	PRIDE: 8653
			MAMAHAGLCGWR	PRIDE: 8653
ENST00000399070	>10	heart, 4.87	AFSTCTDLIEHQK	PRIDE: 8658
			CDACGKAFSTCTDLIEHQK	PRIDE: 8665
			ECGKSFSR	PRIDE: 1805
			HQRIHTGK	PRIDE: 1831
			IHTGEKPY	PRIDE: 1789; 1828; 1830; 1831
			IHTGKK	
			NPIPVINVAK	PRIDE: 2027
			PLVCTPTLFSTRDTVPEKNLMNA	PRIDE: 8660
			VDY	PRIDE: 8663
			PNPCDECGKSFSR	PRIDE: 8663; 8665; 8671
			PSVGFQILLIINEFTLER	PRIDE: 8658
			PSVRAQILFCIR	PRIDE: 8658
			PYKCDACGKAFSTCTDLIEHQK	PRIDE: 9308
			QKIHTGEK	PRIDE: 76

			SDLIKHQR	PRIDE: 1824
ENST00000400385	4	testis, 1.32	DHQGSVEDTSLGGDAPADGVSPS	PRIDE: 146
			VPPLQGLGK	PRIDE: 8663
			LSTSSCASVSR	PRIDE: 8385; 8417
			PMHGSDFPPR	PRIDE: 9340
			PPSPAHSREVAS	PRIDE: 8538
			RASRLALQGPPGTILSLSSSSPC	PRIDE: 8665
			RSGGPR	
ENST00000400449	1	breast, 0.93	KGMEWPLSAWLESSSPAPCMPLWR	PRIDE: 8661; 8664; 8669; 8670
			LFSVHWQIWGPCSPCIVYPKGDSEQSASK	PRIDE: 8663; 8669; 8670
			MPSQPHLSACSVESPGAAARLTQR	PRIDE: 8669
			NSFWESRPSPCR	PRIDE: 8653
ENST00000400991	>10	brain, 2.93	PLTAPSTK	PRIDE: 76
ENST00000408893	3	fat, 0.85	MGLCTLQPLGPPRK	PRIDE: 8671
			SSTSCGTWTASGLPSLGHLPRR	PRIDE: 8667
ENST00000408897	4	testis, 1.49	GSCPLLPGPSAWR	PRIDE: 8666
			MEGCAVR	PRIDE: 8653
			MEGCAVRRGSCPLLPGPSAWR	PRIDE: 9297; 9304; 9305; 9306; 9334; 9335; 9336; 9337; 9338
			SWCRASGLPNR	PRIDE: 9353
ENST00000408913	4	lymph, 17.49	AALPPPLGTCRPR	PRIDE: 1789
			DSLPPLGSLTLGK	PRIDE: 2001; 9330
			DSLPPLGSLTLGKSPK	PRIDE: 1789
			LASAFLHR	PRIDE: 10049
			MNTYTRSASFTPK	PRIDE: 1789
			NTYTRSASFTPK	PRIDE: 1789
			RAALPPPLGTCR	PRIDE: 8653
			RAALPPPLGTCRPR	PRIDE: 1789
			VSEGLRDSLPPLGSLTLGK	PRIDE: 1789
			YLGRPSPR	PRIDE: 96
			YLGRPSPRR	PRIDE: 9333

[#]For each *de novo* gene, the highest RPKM score was shown with the corresponding tissue. [@]Genes reported in previous study as human-specific *de novo* protein-coding genes.
