# Supplementary material for: Hominoid-Specific De Novo Protein-Coding Genes Originating from Long Non-Coding RNAs
Source: PLoS Genet. 2012 Sep 13;8(9):e1002942. doi: 10.1371/journal.pgen.1002942 (PMC3441637; doi:10.1371/journal.pgen.1002942)
Supplement: Table S4 — De novo genes with different parameters in the computational identification. (PDF) [file pgen.1002942.s015.pdf]

Table S4: *De novo* genes with different parameters in the computational identification

Ensembl ID	50% cutoff	Coding exon	Lowest E-vlaue*	Alu Element
ENST00000273641	Yes	4	>1	Yes
ENST00000308946	Yes	2	>1	No
ENST00000315302	No	1	>1	No
ENST00000318659	Yes	1	>1	No
ENST00000324987	Yes	1	>1	No
ENST00000326341@	No	1	>1	No
ENST00000327903	Yes	2	>1	No
ENST00000370523	Yes	2	>1	No
ENST00000370535	Yes	3	>1	No
ENST00000373170	Yes	1	>1	No
ENST00000376812@	Yes	1	>1	Yes
ENST00000377006	No	1	0.317	No
ENST00000377064	No	1	>1	No
ENST00000391430	No	1	>1	Yes
ENST00000391812	Yes	1	0.809	Yes
ENST00000397571	Yes	2	>1	No
ENST00000397608	Yes	1	>1	No
ENST00000399070	No	1	0	Yes
ENST00000400385	No	1	0.092	Yes
ENST00000400449	No	1	>1	Yes
ENST00000400991	Yes	1	>1	Yes
ENST00000408893	Yes	1	>1	No
ENST00000408897	Yes	1	>1	No
ENST00000408913	No	1	>1	No

*Lowest E-values identified in BLASTP searches.

@Genes reported in previous study as human-specific *de novo* protein-coding genes.
