# Supplementary material for: Hominoid-Specific De Novo Protein-Coding Genes Originating from Long Non-Coding RNAs
Source: PLoS Genet. 2012 Sep 13;8(9):e1002942. doi: 10.1371/journal.pgen.1002942 (PMC3441637; doi:10.1371/journal.pgen.1002942)
Supplement: Table S5 — De novo genes partially contributed by Alu elements. (PDF) [file pgen.1002942.s016.pdf]

Table S5: *De novo* genes partially contributed by *Alu* elements

Ensembl ID	*Alu* name	Orientation	Gene region	Relative position[#]	Relative length[*]
ENST00000273641	*FRAM*	antisense	5' UTR	11.6%	8.2%
ENST00000376812[@]	*AluJo*	sense	3' UTR	96.6%	3.4%
ENST00000391430	*AluSq; AluSx*	antisense; sense	3' UTR	24.8%	31.7%
ENST00000391812	*AluSg*	antisense	5' UTR	8.8%	6.8%
ENST00000399070	*FRAM; AluSg/x*	sense	3' UTR	45.4%	7.4%
ENST00000400385	*FRAM/FAM*	antisense	3' UTR	97.7%	1.2%
ENST00000400449	*AluSg*	sense	3' UTR	82.6%	17.4%
ENST00000400991	*AluY*	antisense	3' UTR	96.9%	3.2%

[#]Position of the first *Alu* on the transcript;

[*]Percentage of transcript contributed by *Alu* element;

[@]Gene reported in previous study as human-specific *de novo* protein-coding gene.
