# Supplementary material for: Hominoid-Specific De Novo Protein-Coding Genes Originating from Long Non-Coding RNAs
Source: PLoS Genet. 2012 Sep 13;8(9):e1002942. doi: 10.1371/journal.pgen.1002942 (PMC3441637; doi:10.1371/journal.pgen.1002942)
Supplement: Table S6 — Representative regions selected to calculate the expression levels of de novo genes. (PDF) [file pgen.1002942.s017.pdf]

Table S6: Representative regions selected to calculate the expression levels of *de novo* genes.

Ensembl ID	Human*	Chimpanzee	Rhesus Macaque
ENST00000273641	chr2:11162893-11163239,<br>chr2:11178339-11178539,<br>chr2:11182159-11182362,<br>chr2:11186297-11186463,<br>chr2:11186560-11186675,<br>chr2:11189591-11189753	chr2a:11318214-11318571,<br>chr2a:11333709-11333909,<br>chr2a:11337537-11337742,<br>chr2a:11341934-11342100,<br>chr2a:11342197-11342312,<br>chr2a:11345856-11346018	chr13:11182337-11182691,<br>chr13:11197944-11198147,<br>chr13:11201731-11201952,<br>chr13:11205923-11206093,<br>chr13:11206189-11206292,<br>chr13:11209130-11209288
ENST00000308946	chr11:68819369-68819538,<br>chr11:68819634-68821329	chr11:67557667-67557836,<br>chr11:67557932-67559628	chr14:5466482-5466651,<br>chr14:5464701-5466386
ENST00000315302	chr4:183299800-183301692,<br>chr4:183301849-183301991,<br>chr4:183302485-183302613	chr4:186783382-186785278,<br>chr4:186785435-186785579,<br>chr4:186786087-186786215	chr5:174255693-174257561,<br>chr5:174257718-174257862,<br>chr5:174258381-174258509
ENST00000318659	chr3:162572565-162573362	chr3:166455801-166456598	chr2:126309577-126310376
ENST00000324987	chr6:3235099-3237217	chr6:3314292-3316406	chr4:3129731-3131964
ENST00000326341#	chr22:23155177-23156344,<br>chr22:23156876-23157530,<br>chr22: 23158336-23158398	chr22:23040756-23041923,<br>chr22:23042453-23043109,<br>chr22:23043915-23043977	chr10:67840525-67841680,<br>chr10:67842212-67842863,<br>chr10: 67843667-67843729
ENST00000327903	chr22:29696662-29697765,<br>chr22:29698840-29699587,<br>chr22:29701155-29702049	chr22:29838516-29839619,<br>chr22:29840696-29841443,<br>chr22:29844038-29844932	chr10:74846640-74847751,<br>chr10:74848824-74849569,<br>chr10:74851017-74851922
ENST00000370523	chr20:60561212-60561317,<br>chr20:60578095-60578416	chr20:60346750-60346855,<br>chr20:60367307-60367628	chr10:1973952-1974057,<br>chr10:1956435-1956743
ENST00000370535	chrX:139619589-139619636,<br>chrX:139621451-139621487,<br>chrX:139623347-139623614	chrX:140151058-140151101,<br>chrX:140152907-140152943,<br>chrX:140155220-140155489	chrX:138980387-138980434,<br>chrX:138982250-138982286,<br>chrX:138984126-138984395
ENST00000373170	chr6:40454053-40454614,<br>chr6:40454998-40455616	chr6:41317390-41317941,<br>chr6:41318326-41318944	chr4:40204225-40204786,<br>chr4:40205174-40205850
ENST00000376812#	chr12:122977700-122978886,<br>chr12:122979087-122979774,<br>chr12:122979984-122980163,<br>chr12:122984564-122985120,<br>chr12:122985266-122985484	chr12:125800755-125801941,<br>chr12:125802142-125802820,<br>chr12:125803030-125803209,<br>chr12:125807596-125808139,<br>chr12:125808285-125808503	chr11:125230136-125231319,<br>chr11:125231520-125232213,<br>chr11:125232423-125232602,<br>chr11:125237011-125237550,<br>chr11:125237696-125237912
ENST00000377006	chr19:55245657-55246263,<br>chr19:55256868-55257068,<br>chr19:55261143-55261862	chr19:55800746-55801352,<br>chr19:55811964-55812164	chr19:56325925-56326524,<br>chr19:56340240-56340444,<br>chr19:56342750-56343504
ENST00000377064	chr11:65149327-65149451,<br>chr11:65149557-65149625	chr11:64066128-64066252,<br>chr11:64066358-64066426	chr14:8796811-8796965,<br>chr14:8796637-8796705
ENST00000391430	chr5:14767788-14769601	chr5:14987305-14989115	chr6:14904711-14906534
ENST00000391812	chr19:55992773-55994027,	chr19:56468126-56469370,	chr19:57001245-57002480,

	chr19:55994362-55994426,	chr19:56469709-56469773,	chr19:57002813-57002877,
	chr19:55997285-55997326,	chr19:56472625-56472666,	chr19:57004992-57005033,
	chr19:55997522-55997683,	chr19:56472862-56473023,	chr19:57005229-57005390,
	chr19:55999640-55999786	chr19:56475186-56475332	chr19:57007779-57007925
ENST00000397571	chr17:74526885-74527293,	chr17:78710401-78710809,	chr16:74276279-74276687,
	chr17:74527682-74527913,	chr17:78711198-78711429,	chr16:74277077-74277309,
	chr17:74535052-74535278	chr17:78718559-78718785	chr16:74285073-74285292
ENST00000397608	chr7:136205091-136207250	chr7:137438063-137440223	chr3:174372647-174374811
ENST00000399070	chr18:31124243-31124353,	chr18:31263448-31263558,	chr18:28262421-28262531,
	chr18:31124971-31125194,	chr18:31264176-31264399,	chr18:28263163-28263386,
	chr18:31139937-31144728	chr18:31279137-31283985	chr18:28285048-28289941
ENST00000400385	chr21:44050113-44050897,	chr21:43441344-43442138,	chr3:2906702-2907541,
	chr21:44053109-44056876,	chr21:43444354-43448206	chr3:2900668- 2904541,
ENST00000400991	chr1:154648274-154650714	chr1:135621696-135624139	chr1:135016694-135019117
ENST00000400449	chr21:42186897-42188304,	chr21:41605224-41606690,	chr3:4776075-4777524,
	chr21:42188374-42188568	chr21:41606760-41606954	chr3:4775785-4776005
ENST00000408893	chr1:245341435-245342149	chr1:228229938-228230673	chr1:209869562-209870340
ENST00000408897	chr4:166097624-166098098	N.A.	chr5:157174651-157175133
ENST00000408913	chr14:68332263- 68332544,	chr14:68375912- 68376198,	chr7:131901534- 131901835,
	chr14: 68332713-68332764	chr14: 68376367-68376416	chr7: 131902004-131902055

[#]Genes reported in previous study as human-specific de novo protein-coding genes.
[*]Coordinates based on human genome hg18, chimpanzee genome panTro2 and rhesus macaque genome rheMac2.
