# Supplementary material for: Hominoid-Specific De Novo Protein-Coding Genes Originating from Long Non-Coding RNAs
Source: PLoS Genet. 2012 Sep 13;8(9):e1002942. doi: 10.1371/journal.pgen.1002942 (PMC3441637; doi:10.1371/journal.pgen.1002942)
Supplement: Table S7 — Statistics of public RNA-Seq data integrated in this study. (PDF) [file pgen.1002942.s018.pdf]

Table S7: Statistics of public RNA-Seq data integrated in this study

Tissue	Description	Total Reads	Unique Reads		SRA
Adipose (H*)	32 bp, single-end, Strand-nonspecific	27.8 M#	18.1 M	65.2%	SRX003932
Skeletal Muscle (H)	32 bp, single-end, Strand-nonspecific	22.6 M	15.0 M	66.2%	SRX003930
Testis (H)	76 bp, single-end, Strand-nonspecific	32.4 M	19.2 M	59.1%	SRR306858
Frontal Cortex (H)	76 bp, single-end, Strand-nonspecific	24.3 M	14.4 M	59.1%	SRR306841
Cerebellum (H)	76 bp, single-end, Strand-nonspecific	46.8 M	25.7 M	54.9%	SRR306845, SRR306846
Heart (H)	76 bp, single-end, Strand-nonspecific	30.9 M	16.1 M	52.0%	SRR306848, SRR306849
Liver (H)	76 bp, single-end, Strand-nonspecific	23.9 M	15.2 M	63.9%	SRR306856
Liver (C)	35 bp, single-end, Strand-nonspecific	6.4 M	4.5 M	70.0%	SRR032139
Testis (C)	76 bp, single-end, Strand-nonspecific	26.7 M	13.1 M	49.0%	SRR306825
Frontal Cortex (C)	100 bp, paired-end, Strand-nonspecific	22.2 M	16.6 M	74.9%	SRR306815
Cerebellum (C)	76 bp, single-end, Strand-nonspecific	19.4 M	10.8 M	55.7%	SRR306818
Heart (C)	76 bp, single-end, Strand-nonspecific	43.1 M	20.1 M	46.7%	SRR306820
Heart (R)	76 bp, single-end, Strand-nonspecific	20.8 M	8.8 M	42.5%	SRR306783
Cerebellum (R)	76 bp, single-end, Strand-nonspecific	21.1 M	10.9 M	51.7%	SRR306781
**Summary**		**368.4 M**	**208.5 M**	**56.6%**	

*R: Rhesus macaque, C: Chimpanzee, H: Human.
#M: million reads.
