# Supplementary material for: Hominoid-Specific De Novo Protein-Coding Genes Originating from Long Non-Coding RNAs
Source: PLoS Genet. 2012 Sep 13;8(9):e1002942. doi: 10.1371/journal.pgen.1002942 (PMC3441637; doi:10.1371/journal.pgen.1002942)
Supplement: Table S8 — Expression of de novo genes in human and rhesus macaque. (PDF) [file pgen.1002942.s019.pdf]

Table S8: Expression of *de novo* genes in human and rhesus macaque.

Ensembl ID	Human	Rhesus macaque
ENST00000273641	1.80[&]	-2.32
ENST00000308946	0.86	-2.32
ENST00000315302	0.80	2.05
ENST00000318659	1.29	1.18
ENST00000324987	0.81	-0.09
ENST00000326341[@]	4.08	3.57
ENST00000327903	6.18	6.58
ENST00000370523	4.68	5.29
ENST00000370535	4.62	4.96
ENST00000373170	5.05	3.93
ENST00000376812[@]	3.62	1.97
ENST00000377006	0.25	0.08
ENST00000377064	2.28	0.31
ENST00000391430	3.21	2.63
ENST00000391812	5.84	5.08
ENST00000397571	0.68	-2.32
ENST00000397608	1.11	-1.25
ENST00000399070	4.03	3.99
ENST00000400385	2.12	-2.32
ENST00000400449	-2.25	-0.27
ENST00000400991	4.22	4.7
ENST00000408893	0.96	0.3
ENST00000408897	2.57	-0.67
ENST00000408913	3.56	-0.64

[&]Log_2 transformed summed RPKM in seven tissues (adipose, skeletal muscle, heart, testis, prefrontal cortex, cerebellum and liver) were shown.
[@]Genes reported in previous study as human-specific *de novo* protein-coding genes.
