# Supplementary material for: Hominoid-Specific De Novo Protein-Coding Genes Originating from Long Non-Coding RNAs
Source: PLoS Genet. 2012 Sep 13;8(9):e1002942. doi: 10.1371/journal.pgen.1002942 (PMC3441637; doi:10.1371/journal.pgen.1002942)
Supplement: Table S9 — Human splicing junctions supported by expression evidence. (PDF) [file pgen.1002942.s020.pdf]

Table S9: Human splicing junctions supported by expression evidence

Ensembl ID	Position of Splicing Junction	Class	mRNA	Spliced EST[&]	Junction Reads
		**Class I**			
ENST00000273641	chr2:11182362:11186298	CDS-CDS	AK090853, AX746649	NA	5
ENST00000273641	chr2:11186463:11186561	5UTR-5UTR	AK311163	DA335795, DA196360	2
ENST00000273641	chr2:11157640:11162894	3UTR-3UTR	AK090853, AX746649	NA	0
ENST00000273641	chr2:11163239:11178340	CDS-CDS	AK090853, AX746649	NA	0
ENST00000273641	chr2:11178539:11182160	CDS-CDS	AK090853, AX746649	NA	0
ENST00000273641	chr2:11186675:11189592	5UTR-5UTR	NA	DA196360	0
ENST00000308946	chr11:68819538:68819635	CDS-CDS	AK290121, BC011815	BM837864, DA377733, BM787974, DA645878, BU543662, BF314411, BI870785, CV028486, BE259975	1
ENST00000308946	chr11:68818619:68819370	5UTR-5UTR	AK290121, BC011815	DA021966, BM837864, CB125910, CB124563, DA906730, DA377733, BM837305, BM833038, BM787974, DA645878	0
ENST00000326341[@]	chr22:23157530:23158230	5UTR-5UTR	AK091970, AX747284	DA635985	57
ENST00000326341[@]	chr22:23156344:23156877	3UTR-3UTR	AK091970, AX747284	NA	0
ENST00000370535	chrX:139621487:139623348	CDS-CDS	AK054976	DA082671, BP872202, BX488474, DA060415, DA359433, DA070595, DA079845, DA086733, DT216583, CD239672	46
ENST00000370535	chrX:139619636:139621452	CDS-CDS	AK054976	DA082671, BP872202, BX488474, DA060415, DA359433, DA070595, DA079845, DA086733, DT216583, CD239672	42
ENST00000376812	chr12:122981043:122984565	3UTR-3UTR	AK127211	NA	0
ENST00000399070	chr18:31124353:31124972	5UTR-5UTR	AF373036, AF153201, AK023456	BF212348, DA479011, DA005750, DA736321, DA576313, DA733689, DA748162, DA831167,	160

ENST00000399070	chr18:31125194:31139938	5UTR-5UTR	AF373036, AF153201, AK023456, AK094216	DA683872, DA517516 BF212348, DA479011, DA005750, DA736321, DA576313, DA733689, DA748162, DA831167, DA683872, DA517516	105
ENST00000400385	chr21:44050897:44053110	3UTR-3UTR	AK128598, BC031911	W05706	2
ENST00000400449	chr21:42188304:42188375	5UTR-5UTR	AK129520	NA	0
ENST00000400991	chr1:154651169:154663214	5UTR-5UTR	AK095626	DA497813, DA503916, DA319681, DB481954, DA797978, AW163715, DB467631, C15680, AA021414, EL950080	599
ENST00000400991	chr1:154663288:154665792	5UTR-5UTR	AK095626	DA497813, DA503916, DA319681, DB481954, DA797978, AW163715, DB467631, AA021414, AA058881, EL954532	266
ENST00000400991	chr1:154650714:154651070	5UTR-5UTR	AK095626	DA497813, DA503916, DA319681, DB481954, DA797978, AW163715, DB467631, C15680	6
		**Class II**			
ENST00000315302	chr4:183301692:183301850	5UTR-5UTR	AK024300	DA682898, AU132776, DB240569, DA219596, DA750697, DA604937, DA227075, DA757778, CN360795	2
ENST00000315302	chr4:183301991:183302194	5UTR-5UTR	AK024300	AU132776, CN360795	0
ENST00000315302	chr4:183302287:183302486	5UTR-5UTR	AK024300	AU132776, CN360795	0
ENST00000318659	chr3:162546837:162560284	3UTR-3UTR	BC037817	CA455115, CA487941, BU539808, BU959155, BG287503, BI766630, AV722799, CD692285, CX751494, BU858267	49
ENST00000318659	chr3:162560376:162571917	3UTR-3UTR	BC037817	CA455115, CA487941, BU539808, BU959155, BG287503, BI766630, AV722799, CD692285, CX751494, BU858267	47
ENST00000324987	chr6:3204429:3235032	5UTR-5UTR	AK095683	DA513520	0
ENST00000327903	chr22:29697765:29698841	CDS-CDS	AK056480	BM541501, DA744877, DB054609, DA812164,	112

ENST00000327903	chr22:29699587:29701156	3UTR-3UTR	AK056480	CA420106, AW390779, AW362039, DB244832, DA571445, EL734313 AW604129, CB141510, BP240227, DA832964, DA096209, BX643169, AW373835, DB070372	27
ENST00000370523	chr20:60561317:60578096	CDS-CDS	NA	NA	1
ENST00000373170	chr6:40454614:40454999	5UTR-5UTR	BC042123, BC033995, BC071820, DQ168992	BG701637, BI561942, BI459675, BU561761, AL045589, AA293846, BG183126, BU621961	55
ENST00000377006	chr19:55257068:55261144	3UTR-3UTR	AK130360	NA	1
ENST00000377006	chr19:55246263:55256869	3UTR-3UTR	AK130360, BC130542, BC130544	NA	0
ENST00000391812	chr19:55994027:55994363	5UTR-5UTR	BC006151, BC037227, BC051842, AY491972	CA916798, BU508032, BX365647, BX375691, BM547408, BM925137, BQ054356, BM906786, AL523972, BX362010	162
ENST00000391812	chr19:55994426:55997286	5UTR-5UTR	BC037227, BC051842	CX165317, BU508032, BX365647, BX375691, BM547408, BM925137, BQ054356, BM906786, AL523972, BX362010	151
ENST00000391812	chr19:55997326:55997523	5UTR-5UTR	BC051842	BM802982, BX376851, BM557337, BG746959, BQ642619, BM802702, BX437995, BE871649, CD634255	5
ENST00000391812	chr19:55997683:55999641	5UTR-5UTR	NA	BM802982	0
ENST00000397571	chr17:74527293:74527683	CDS-CDS	BC039683	BE856800, AI744569, BI837579, BI837517, BX099833, DA945591	2
ENST00000397571	chr17:74527913:74535053	5UTR-5UTR	BC039683	BI837579, BI837517, BX099833, DA945591	0
ENST00000397608	chr7:136204705:136205092	5UTR-5UTR	AK125233	DA821721, DA559483	2
ENST00000397608	chr7:136204460:136204548	5UTR-5UTR	AK125233	DA821721, DA559483	12

[@]Genes reported in previous study as human-specific *de novo* protein-coding genes.
[&]For splicing junctions with enough ESTs (>=10), accession numbers for the top ten ESTs were listed.
