# Supplementary material for: Hominoid-Specific De Novo Protein-Coding Genes Originating from Long Non-Coding RNAs
Source: PLoS Genet. 2012 Sep 13;8(9):e1002942. doi: 10.1371/journal.pgen.1002942 (PMC3441637; doi:10.1371/journal.pgen.1002942)
Supplement: Table S10 — Chimpanzee splicing junctions supported by RNA-Seq reads. (PDF) [file pgen.1002942.s021.pdf]

Table S10: Chimpanzee splicing junctions supported by RNA-Seq reads

Human Ensembl ID	Position of Splicing Junction in Chimpanzee	Class	Junction Reads
**Class I**			
ENST00000273641	chr2a:11337742:11341935	CDS-CDS	0
ENST00000273641	chr2a:11342100:11342198	5'UTR-5'UTR	0
ENST00000308946	chr11:67557836:67557933	CDS-CDS	N.A.[&]
ENST00000326341	chr22:23043109:23043809	5'UTR-5'UTR	31
ENST00000370535	chrX:140152943:140155221	CDS-CDS	90
ENST00000370535	chrX:140151101:140152908	CDS-CDS	62
ENST00000399070	chr18:31263558:31264177	5'UTR-5'UTR	103
ENST00000399070	chr18:31264399:31279138	5'UTR-5'UTR	50
ENST00000400385	chr21:43442138:43444355	3'UTR-3'UTR	0
ENST00000400991	chr1:135624594:135637188	5'UTR-5'UTR	156
ENST00000400991	chr1:135637262:135639756	5'UTR-5'UTR	31
ENST00000400991	chr1:135624139:135624495	5'UTR-5'UTR	11
**Class II**			
ENST00000315302	chr4:186785278:186785436	5'UTR-5'UTR	11
ENST00000318659	chr3:166430191:166443875	3'UTR-3'UTR	40
ENST00000318659	chr3:166443967:166455153	3'UTR-3'UTR	35
ENST00000327903	chr22:29839619:29840697	CDS-CDS	139
ENST00000327903	chr22:29841443:29844039	3'UTR-3'UTR	27
ENST00000370523	chr20:60346855:60367308	CDS-CDS	0
ENST00000373170	chr6:41317941:41318327	5'UTR-5'UTR	5
ENST00000377006	N.A.	3'UTR-3'UTR	N.A.
ENST00000391812	chr19:56469370:56469710	5'UTR-5'UTR	101
ENST00000391812	chr19:56469773:56472626	5'UTR-5'UTR	64
ENST00000391812	chr19:56472666:56472863	5'UTR-5'UTR	1
ENST00000397571	chr17:78710809:78711199	CDS-CDS	3
ENST00000397608	chr7:137437677:137438064	5'UTR-5'UTR	1
ENST00000397608	chr7:137437432:137437520	5'UTR-5'UTR	6

[&]No orthologous regions found in rhesus macaque or no reads mapped to the splicing junctions.
