# Supplementary material for: Hominoid-Specific De Novo Protein-Coding Genes Originating from Long Non-Coding RNAs
Source: PLoS Genet. 2012 Sep 13;8(9):e1002942. doi: 10.1371/journal.pgen.1002942 (PMC3441637; doi:10.1371/journal.pgen.1002942)
Supplement: Table S11 — Monkey splicing junctions supported by RNA-Seq reads. (PDF) [file pgen.1002942.s022.pdf]

Table S11: Monkey splicing junctions supported by RNA-Seq reads

Ensembl ID	Position of Splicing Junction in Rhesus Macaque	Class	Junction Reads
**Class I**			
ENST00000273641	chr13:11201952:11205924	CDS-CDS	0
ENST00000273641	chr13:11206093:11206191	5’UTR-5’UTR	N.A. [&]
ENST00000308946	chr14:5466386:5466483	CDS-CDS	0
ENST00000326341	chr10:67842863:67843561	5’UTR-5’UTR	215
ENST00000370535	chrX:138982286:138984127	CDS-CDS	160
ENST00000370535	chrX:138980434:138982251	CDS-CDS	137
ENST00000399070	chr18:28262531:28263164	5’UTR-5’UTR	48
ENST00000399070	chr18:28263386:28285049	5’UTR-5’UTR	3
ENST00000400385	chr3:2904541:2906703	3’UTR-3’UTR	0
ENST00000400991	chr1:135019571:135031218	5’UTR-5’UTR	2827
ENST00000400991	chr1:135031292:135033756	5’UTR-5’UTR	3457
ENST00000400991	chr1:135019117:135019472	5’UTR-5’UTR	0
**Class II**			
ENST00000315302	chr5:174257561:174257719	5’UTR-5’UTR	12
ENST00000318659	chr2:126311025:126342815	3’UTR-3’UTR	0
ENST00000318659	N.A.	3’UTR-3’UTR	N.A.
ENST00000327903	chr10:74847751:74848825	CDS-CDS	1351
ENST00000327903	chr10:74849569:74851018	3’UTR-3’UTR	339
ENST00000370523	chr10:1956743:1973953	CDS-CDS	0
ENST00000373170	chr4:40204786:40205175	5’UTR-5’UTR	118
ENST00000377006	chr19:56340444:56342751	3’UTR-3’UTR	0
ENST00000391812	chr19:57002480:57002814	5’UTR-5’UTR	0
ENST00000391812	chr19:57002877:57004993	5’UTR-5’UTR	218
ENST00000391812	chr19:57005033:57005230	5’UTR-5’UTR	3
ENST00000397571	chr16:74276687:74277078	CDS-CDS	N.A.
ENST00000397608	chr3:174372019:174372107	5’UTR-5’UTR	47
ENST00000397608	chr3:174372264:174372648	5’UTR-5’UTR	0

[&]No orthologous regions found in rhesus macaque or no reads mapped to the splicing junctions.
